# Evaluation of PacBio sequencing for full-length bacterial 16S rRNA gene classification

**DOI:** 10.1186/s12866-016-0891-4

**Published:** 2016-11-14

**Authors:** Josef Wagner, Paul Coupland, Hilary P. Browne, Trevor D. Lawley, Suzanna C. Francis, Julian Parkhill

**Affiliations:** 1 Wellcome Trust Sanger Institute, Hinxton, CB10 1SA Cambridge, UK; 2 Tropical Epidemiology Group, London Schools of Hygiene and Tropical Medicine, London, UK

**Keywords:** PacBio sequencing, MiSeq sequencing, Capillary sequencing, Full-length bacterial 16S rRNA gene sequencing

## Abstract

**Background:**

Currently, bacterial 16S rRNA gene analyses are based on sequencing of individual variable regions of the 16S rRNA gene (Kozich, et al *Appl Environ Microbiol* 79:5112–5120, 2013).This short read approach can introduce biases. Thus, full-length bacterial 16S rRNA gene sequencing is needed to reduced biases. A new alternative for full-length bacterial 16S rRNA gene sequencing is offered by PacBio single molecule, real-time (SMRT) technology. The aim of our study was to validate PacBio P6 sequencing chemistry using three approaches: 1) sequencing the full-length bacterial 16S rRNA gene from a single bacterial species *Staphylococcus aureus* to analyze error modes and to optimize the bioinformatics pipeline; 2) sequencing the full-length bacterial 16S rRNA gene from a pool of 50 different bacterial colonies from human stool samples to compare with full-length bacterial 16S rRNA capillary sequence; and 3) sequencing the full-length bacterial 16S rRNA genes from 11 vaginal microbiome samples and compare with in *silico* selected bacterial 16S rRNA V1V2 gene region and with bacterial 16S rRNA V1V2 gene regions sequenced using the Illumina MiSeq.

**Results:**

Our optimized bioinformatics pipeline for PacBio sequence analysis was able to achieve an error rate of 0.007% on the S*taphylococcus aureus* full-length 16S rRNA gene. Capillary sequencing of the full-length bacterial 16S rRNA gene from the pool of 50 colonies from stool identified 40 bacterial species of which up to 80% could be identified by PacBio full-length bacterial 16S rRNA gene sequencing. Analysis of the human vaginal microbiome using the bacterial 16S rRNA V1V2 gene region on MiSeq generated 129 operational taxonomic units (OTUs) from which 70 species could be identified. For the PacBio, 36,000 sequences from over 58,000 raw reads could be assigned to a barcode, and the *in silico* selected bacterial 16S rRNA V1V2 gene region generated 154 OTUs grouped into 63 species, of which 62% were shared with the MiSeq dataset. The PacBio full-length bacterial 16S rRNA gene datasets generated 261 OTUs, which were grouped into 52 species, of which 54% were shared with the MiSeq dataset. Alpha diversity index reported a higher diversity in the MiSeq dataset.

**Conclusion:**

The PacBio sequencing error rate is now in the same range of the previously widely used Roche 454 sequencing platform and current MiSeq platform. Species-level microbiome analysis revealed some inconsistencies between the full-length bacterial 16S rRNA gene capillary sequencing and PacBio sequencing.

**Electronic supplementary material:**

The online version of this article (doi:10.1186/s12866-016-0891-4) contains supplementary material, which is available to authorized users.

## Background

Currently, large-scale bacterial 16S rRNA gene analyses are based on sequencing of individual variable regions of the bacterial 16S rRNA gene. Single variable regions and combinations of variable regions including V2, V3, V4, V1-2, V1-3, V2-3, V2-4, V3-4, V4-5, V3-6, and V7-9, have been all sequenced with second generation sequencing technology offered previously by Roche and more recently by Illumina technologies [1–5]. However, depending on the region sequenced, biases can be introduced [5–7].

Illumina MiSeq sequencing is currently the most widely applied platform for bacterial 16S rRNA gene amplicon sequencing. Its latest chemistry allows the sequencing of 300 bp PCR fragments in both directions with full overlap and makes it appropriate for the sequencing of single variable regions and the bacterial 16S rRNA V1-2 gene region where full sequence overlap is desired [1]. However, the short read approach by second generation sequencing introduces biases depending on which variable regions are used and can not provide effective resolution below the bacterial genus level, limiting microbial ecology studies [5, 8]. Further, a large proportion of the bacterial 16S rRNA gene records in the GenBank database labeled as environmental samples are unclassified, which is in part due to low read accuracy, potential chimeric sequences produced during PCR amplification and the low resolution of short amplicons. High throughput full-length bacterial 16S rRNA gene sequencing methodologies with reduced biases are needed.

Historically, we were able to sequence full-length bacterial 16S rRNA gene by conventional cloning and Sanger sequencing. However, this is laborious, costly, and is low throughput. A new alternative for full-length bacterial 16S rRNA gene sequencing is offered by the third generation Pacific Biosciences single molecule, real-time (SMRT) sequencing technology. The latest PacBio P6/C4 chemistry offers very long reads, where half of the reads >14,000 base pairs long and each SMRT cell yields an average of 50,000 reads from a 4 hour run (www.pacb.com/smrt-science/smrt-sequencing/). Phylogenetic profiling based on full-length bacterial 16S  rRNA gene requires high read accuracy, and this should be achieved through the use of PacBio circular consensus sequencing (CCS). In CCS, the DNA polymerase reads a ligated circular DNA template multiple times, depending on amplicon size, read length and sequencing movie length [9], effectively generating a consensus sequence from multiple reads of a single molecule.

Few studies have investigated the efficacy of PacBio 3rd generation sequencing for full-length bacterial 16S rRNA genes using CCS. Mosher and colleagues reported the first sequencing of full-length bacterial 16S rRNA gene amplicons from environmental samples (sediment and rock biofilm) using the PacBio RS SMRT sequencing platform with XL/C2 chemistry in comparison with the Roche 454 GS FLX chemistry [10]. The Roche 454 bacterial 16S rRNA V1V3 gene sequences could be grouped into 57 clusters (operational taxonomic units (OTUs)) at 97% sequence identity, whereas the PacBio bacterial 16S rRNA V1V3 gene sequences were grouped into 594 clusters (OTUs) at 97% sequence identity. The PacBio full-length bacterial 16S  rRNA gene sequences were grouped into 755 clusters (OTUs) at 97% sequence identity. The >10-fold increase in the number of OTU clusters obtained from the PacBio platform reflected the large sequencing error rate in the PacBio data at that time. One year later the same group reanalyzed two of their libraries with the updated P4/C2 chemistry and a run time of 180 minutes [11]. The PacBio RS II platform combined with P4/C2 chemistry improved sequence accuracy and number of high quality reads, compared to their initial assessment, from 80% to > 99% for the full length bacterial 16S rRNA gene sequence. In addition the number of reads obtained from the sediment sample doubled from 4563-5955 sequences to 8260-12057 sequences when using the newer chemistry.

Singer and colleagues reported the comparison of PacBio full-length bacterial 16S rRNA gene sequencing with Illumina HiSeq 2500 metagenomic shotgun sequencing and MiSeq bacterial 16S rRNA V4 gene region sequencing using a mock community as well as an environmental sample from Sakinaw Lake, British Columbia [12]. When they compared the PacBio full-length bacterial 16S rRNA gene taxonomic resolution with *in silico* generated PacBio bacterial 16S rRNA V4 gene region at various taxonomic levels, they were able to classify a higher proportion of phylum, class, family, genus and species level from the full-length bacterial 16S rRNA gene dataset compared to the in silico generated PacBio bacterial 16S rRNA V4 gene region.

The group reported minor differences between datasets from Illumina MiSeq bacterial 16S rRNA V4 gene region and PacBio full-length bacterial 16S rRNA gene in the classifiable OTUs. PacBio full-length bacterial 16S rRNA gene sequencing resolved all 23 OTUs in the mock community, whereas Illumina bacterial 16S rRNA V4 gene sequencing could not resolve closely related species.

The most comprehensive analysis of PacBio full-length bacterial 16S rRNA gene sequencing was recently published by Schloss et al. [13]. They sequenced different bacterial 16S rRNA gene regions (V4, V1V3, V3V5, V1V5, V1V6, and V1V9) from a defined mock community and natural samples from human feces, mouse feces and soil environment. Their sequence error rates reported ranged between 0.019% and 0.158% for the above bacterial 16S rRNA gene regions.

The use of full-length sequencing for bacterial 16S rRNA gene analysis would have significant advantages over current approaches. However, only a few studies have investigated the use of PacBio technology for the sequencing of the full-length 16S gene [10–12, 14]. Thus, more information is urgently needed to identify the advantages and disadvantages of PacBio CCS for bacterial full-length 16S rRNA gene sequencing.

Previous studies including the lasted study from Schloss et al. [13] compared PacBio sequencing with Illumina sequencing, both are know to be error prone. In our study we also included a pool of capillary sequenced stool colonies. Thus, the aim of our study was to validate the latest PacBio P6/C4 sequencing chemistry using three approaches: 1) sequencing the full-length bacterial 16S gene from a single bacterial species *Staphylococcus aureus* to analyze error modes and to optimize the bioinformatics pipeline for further analyses 2) sequencing the full-length bacterial 16S rRNA genes from a pool of 50 different bacterial colonies from stool to compare with full-length bacterial 16S rRNA genes capillary sequenced and 3) sequencing the full-length bacterial 16S rRNA genes from 11 vaginal microbiome samples and comparing the PacBio full-length bacterial 16S rRNA genes and *in silico* selected bacterial 16S rRNA V1V2 gene region with bacterial 16S rRNA V1V2 gene regions sequenced using the Illumina MiSeq from the same samples.

## Methods

### PacBio sequencing of *Staphylococcus aureus* 16S DNA

All polymerase chain reactions (PCR) were performed in triplicates using the New England Biolab (NEB) Q5 high-fidelity polymerase kit. Twenty ng of *Staphylococcus aureus* deoxyribonucleic acid (DNA) was PCR amplified in a total reaction volume of 50 μl together with 200 μM dNTPs, 0.5 μM forward primers (7f 5’ AGAGTTTGATYMTGGCTCAG 3’), 0.5 μM reverse primer (1510r 5’ ACGGYTACCTTGTTACGACCT 3’), and 0.25 μl Q5 Taq enzyme. Each PCR was done in triplicate with 20 cycles. Cycling conditions were as follows: Denaturation at 98 °C for 2 minutes, followed by 30 cycles of amplification (denaturation 98 °C for 30 seconds, annealing 50 °C for 30 seconds, extension 72 °C for 90 seconds) and a final extension at 72 °C for 5 minutes. PCR product was purified with 50 μl AMPure XP beads (Agencourt Bioscience) according to Illumina’s 16S metagenomic sequencing library preparation protocol pages 8-9 (Part # 15044223 Rev. B, 11/27/2013) [15]. The amplified DNA was sequenced using the PacBio SMRT sequencing technology according to the standard manufacturers conditions. SMRT bell library prep and sequencing used the currently available reagent kits Template Preparation 3.0, Polymerase Binding P6, and Sequencing Chemistry C4. Data was captured using 3-hour movies.

### PacBio sequencing of bacterial 16S rRNA gene from intestinal microbiome species and human vaginal microbiome

Faecal samples from six healthy human donors were plated by the Host-microbiota Interactions Group at the Wellcome Trust Sanger Institute and from these single colonies were picked and identified using full-length bacterial 16S rRNA gene capillary sequencing [16]. Whole genome DNA was extracted from each unique species and 50 of these were pooled in equal volumes for subsequent analysis.

For the human vaginal microbiome study, DNA from 11 cervico-vaginal lavage cell pellets from a microbicide feasibility study in Tanzania [17] were extracted using FastDNA® Spin Kit for Soil (Qbiogene, Carlsbad, CA, USA) at the London School of Hygiene and Tropical Medicine and were to the Wellcome Trust Sanger Institute. All PCR reactions were performed using the NEB Q5 high-fidelity polymerase kit, as above. Each PCR was done in triplicate with 20 cycles and using the same PCR condition as above. The bacterial full-length 16S rRNA PCR primers included the PacBio barcodes are described here (Additional file 1: Table S1). PCR primers were purchased from Integrated DNA Technology (IDT, Leuven, Belgium). PCR products were purified with AMPure XP beads, as above, and the amplified DNA was sequenced as above. For the human vaginal microbiome study, the DNA concentration was determined by Qubit quantification using Qubit 2.0 flurometer and the high sensitivity DNA reagents (Invitrogen). Samples were pooled in equimolar concentration and gel purified using The Wizard® SV Gel and PCR Clean-Up System (Promega). The library size was confirmed on a Tape station (Agilent Technologies) before PacBio SMRT sequencing as above.

### Illumina MiSeq sequencing of human vaginal microbiome DNA

All PCR reactions were performed using the New England Biolab (NEB) Q5 high-fidelity polymerase kit, as above. The DNA was amplified with Illumina adapter and indexed PCR primers using a dual-index sequencing strategy to target the bacterial V1V2 16S rRNA gene [1]. Each PCR was done in triplicate with 20 cycles with the same cycling conditions as above. The PCR reaction mix contained 200 μM dNTPs, 0.5 μM V1 forward primers (7f 5’ AGMGTTYGATYMTGGCTCAG 3’), 0.5 μM V2 reverse primer (r356 5’ GCTGCCTCCCGTAGGAGT 3’), and 0.25 μl Q5 Taq enzyme. All primers except the read 1 sequencing primer were purchased from IDT (Leuven, Belgium). The read 1 sequencing primer was LNA modified at position 1, 3, and 6 (T + AT + GGT + AATTGTAGMGTTYGATYMTGGCTCAG) and was purchased from Exiqon (Vedbaek, Denmark). PCR product was purified with AMPure XP beads (Agencourt Bioscience) the same as above. The equimolar library mix was prepared the same way as above. The library size was confirmed on a Tape stations (Agilent Technologies) before submitting for MiSeq sequencing using the 600 cycle MiSeq reagent kit V3. The library was sequenced at the Wellcome Trust Sanger Institute (Cambridge, United Kingdom).

### Bioinformatics for PacBio sequence analysis

Raw sequences were initially processed through the PacBio SMRT portal. Sequences were filtered for a minimum of 1-, 2-, 4-, and 8 passes, and a minimum predicted accuracy of 90%. Downstream bioinformatics analysis was performed using the software package Mothur (version 1.34.4) [18], Uchime [19], and ARB – A Software Environment for Sequence Data (version 5.5-org-9167) [20].

The following steps were done in Mothur: The PacBio obtained “fastq.txt” file was first processed with the “fastq.info” command, which generated a fasta and quality file. Both files were then used for amplicon size trimming to remove sequences outside the expected amplicon size (<1400 bp and >1600 bp) using the “trim.seqs” command. A second “trim.seqs” step was performed to remove any ambiguous sequences, sequences with homopolymers longer than 6 bp, and sequences with a quality window average below 35 over a rolling quality window size of 50 bp. Unique sequences were generated and aligned with sequences of the Silva reference database release 119, which is available from the Mothur homepage [21] using the default kmer searching method with the flip parameter set to true. The aligned sequences were screened using the “screen.seqs” command to remove alignment outside the expected alignment coordinates (start = 1046, end = 43116). In order to decrease the sequencing error rate, we also implemented an additional screening step using the align report from the “align.seqs” step and screened the aligned sequences, setting the minscore to 80 or 90 (minimum alignment score) and the minsim to 80 or 90 (minimum similarity score). Then the “filter.seqs” command was used to remove empty columns. The filtered sequences were pre clustered allowing 1% mismatch. The pre clustering implements a pseudo-single linkage algorithm with the goal of removing sequences that are likely due to sequencing errors. As a general rule we allow 1% difference (1 bp/100 bp of the 16S gene) for the bacterial 16S gene. Sequences were checked for chimeras using Chimera Uchime and the template option set to self. High quality filtered sequences were used for matrix generation (dist.seqs command with output = lt) and the phylip generated distance matrix was clustered into operational taxonomic units (OTU) using a cutoff of 0.10. Sequencing error was determined with Mothur using *Staphylococcus aureus* M1 as a reference sequence. High quality sequences and OTUs were classified using Silva.nr_v119.tax database, which is available from the Mothur homepage. All figures were generated in Graph Pad Prism 6 for Mac version 6.0e (GraphPad Software, La Jolla California USA, www.graphpad.com).

### Bioinformatics for MiSeq sequence analysis

A total dataset of over 300 human vaginal microbiome samples were processed. The forward and reverse fastq files were processed according to the MiSeq SOP [22] with additional adjustments: For the “make.contigs” command we set “trimoverlap = T”. No ambiguous sequences were allowed and the maximum number of homopolymers was set to six. Using the Silva bacterial database “silva.nr_v119.align” with the flip parameter set to true aligned the sequences. The “screen.seqs” command was used to remove sequences outside the expected alignment co-ordinates (start = 1148, end = 5716). The subsequent filtered sequences were de-noised by allowing three mismatches in the “pre.clustering” step. Chimeras were removed using Uchime with the dereplicate option set to “true”. Sequences were further classified using the Silva reference database “silva.nr_v119.align” and the Silva taxonomy database “silva.nr_v119.tax” and a cut off value of 80% and the un-classified sequences were removed. The original datasets were screened for any contaminations identified either in the DNA extraction controls or in negative PCR controls. Those contaminants were removed using the “remove.lineage” from the final high quality fasta file, name file and group file. The same 11 samples which were used for the PacBio sequencing were then extracted from the total sample cohort using the “get.group” command and used for the generation of a square distance matrix. The matrix was clustered with the automatic set “cluster.classic” command with following parameters: precision = 100, method = average, hard = T, sim = F. A shared file (OTU table) was generated to identify the number of OTUs at each distance for further analysis.

We also generated a subsampled datasets comparable with PacBio sequencing using the “sub.sample” command. For this we subsampled the final high quality fasta, group and name file to 27596 sequences (number of reads of the PacBio in silico selected V1V2 dataset from the low stringency post alignment screening). The subsampled datasets was again used for matrix generation and clustering as outlined above. OTUs with only one sequence (Singletons OTUs) were considered to originate from PCR errors and sequencing errors and therefore were removed from all PacBio and the MiSeq datasets using the “remove.rare” command in Mothur. The remove.rare command only takes a list and a group file, thus we used the “list.seqs” command to generate an “accnos” file from the singletons free group file. The accnos file was then used to get the singletons free fasta sequences from the fasta and name file using the “get.seqs” command. Both, the total number of OTUs and singletons free OTUs are outlined in Table 1. The singletons free OTUs were used for bacterial taxonomic profile analysis using the ARB software.

**Table 1:** Vaginal human microbiome analysis

	HQR	OTUs 97%	OTUs 98%	OTUs 99%	OTUs unique
PacBio V1V9 Minscore = 90 minsim = 90	20959	60 (48)	63 (49)	90 (62)	209 (148)
PacBio V1V9 Minscore = 80 minsim = 80	23701	181 (83)	453 (136)	1109 (158)	1316 (261)
PacBio in silico V1V2 Minscore = 90 Minsim = 90	26765	82 (60)	131 (65)	426 (113)	486 (131)
PacBio in silico V1V2 Minscore = 80 Minsim = 80	27596	160 (81)	328 (103)	729 (137)	802 (154)
MiSeq V1V2	508016	419 (213)	536 (269)	691 (316)	829 (382)
MiSeq V1V2 Subsampled to 27596	27596	130 (79)	152 (89)	168 (94)	219 (129)

*HQR* high quality reads, *V1V9* full-length bacterial 16S gene, *V1V2* bacterial 16S V1V2 gene region, *minscore* = minimum alignment score during post alignment screening, *minsim* = minimum similarity score during post alignment screening, *OTU* operational taxonomic units, *value in round brackets* number of singleton-free OTUs (OTUs with 1 sequence across all groups were removed)

### Bioinformatics for alpha diversity analysis of the human vaginal microbiome samples

A shared file (OTU table) was generated for the unique and 97% (0.03) OTU distance level from the singletons OTU free list file and group file using the “make.shared” command. For MiSeq analysis we used the subsampled datasets. The “summary.single” command was used to calculate the number of observed OTUs; “sobs”, the estimated richness index; “Chao”, the sample “coverage” index; and three different diversity indexes, namely “inverse Simpson, Shannon and Q statistic (Qstat)”.

We used these three different diversity indexes because they all differ in the mathematical method for the emphasis of taxon (OTU) richness and abundance.

The Simpsons index squares the relative abundance and the weight of rare species will thus be reduced relatively more than that of more abundant species. Thus, the Simpson index gives more weight to common or dominant species and the total species richness is down weighted relative to evenness. The Shannon index includes a log of the relative abundance of species and the weight of abundant species will thus be reduced slightly relative to more rare species in comparison to the Simpsons index. The Q statistic is a bridge between the abundance models and diversity indices and is not weighted towards very abundant or rare species [23]. The Q statistic analysis is based on measuring the “inter-quartile slope” on the cumulative species abundance curve.

## Results

In this study we: 1) evaluate the accuracy for the latest PacBio chemistry P6 using DNA from a single bacterial species (*Staphylococcus aureus*); 2) compared PacBio sequencing with capillary sequencing using a pool of 50 species isolated from human stool samples; and 3) compared PacBio sequencing and Illumina Miseq sequencing using human vaginal microbiome samples. We also evaluated the resolution power of full-length bacterial 16S rRNA gene analysis in identifying candidate species in stool colonies and vaginal microbiome samples. Table 2 shows the number of PacBio filtered CCS reads using 90% minimum predictive accuracy across the different sample sets tested. As expected we observed a steady decrease in the number of reads obtained as filtering progressed from the ≥1 pass filtering to the ≥2 pass, ≥4 pass, and finally ≥8 pass filtering (i.e. the full-length bacterial 16S rRNA gene was sequenced with at least 1x coverage, 2x coverage, 4x coverage, and finally 8X coverage). The largest numbers of sequences were lost between the ≥4 pass and ≥8 pass filtering. Using the ≥8 pass filtered datasets would reduce the number of raw reads by 35.2% (for the *Staphylococcus aureus dataset*), by 36.5% (for stool colonies dataset), and by 43.4% (for the human vaginal samples dataset) compared to the 1 pass dataset (Table 2).

**Table 2:** PacBio raw sequence reads across different sample sets

	Filtered raw sequence reads			
Samples used for sequencing	≥1 Pass	≥2 Passes	≥4 Passes	≥8 Passes
*Staphylococcus aureus*	43268	41756 (13.5%)	37136 (14.2%)	28042 (35.2%)
50 pooled stool colonies	43133	41628 (13.5%)	36839 (14.6%)	27369 (36.5%)
11 bar-coded human	68206	66674 (12.2%)	58951 (13.6%)	38620 (43.4%)
Vaginal samples				

Percent in brackets represent the proportion of sequence decrease compare to the 1 pass dataset

### *Staphylococcus aureus* analysis

**Fig. 1:**
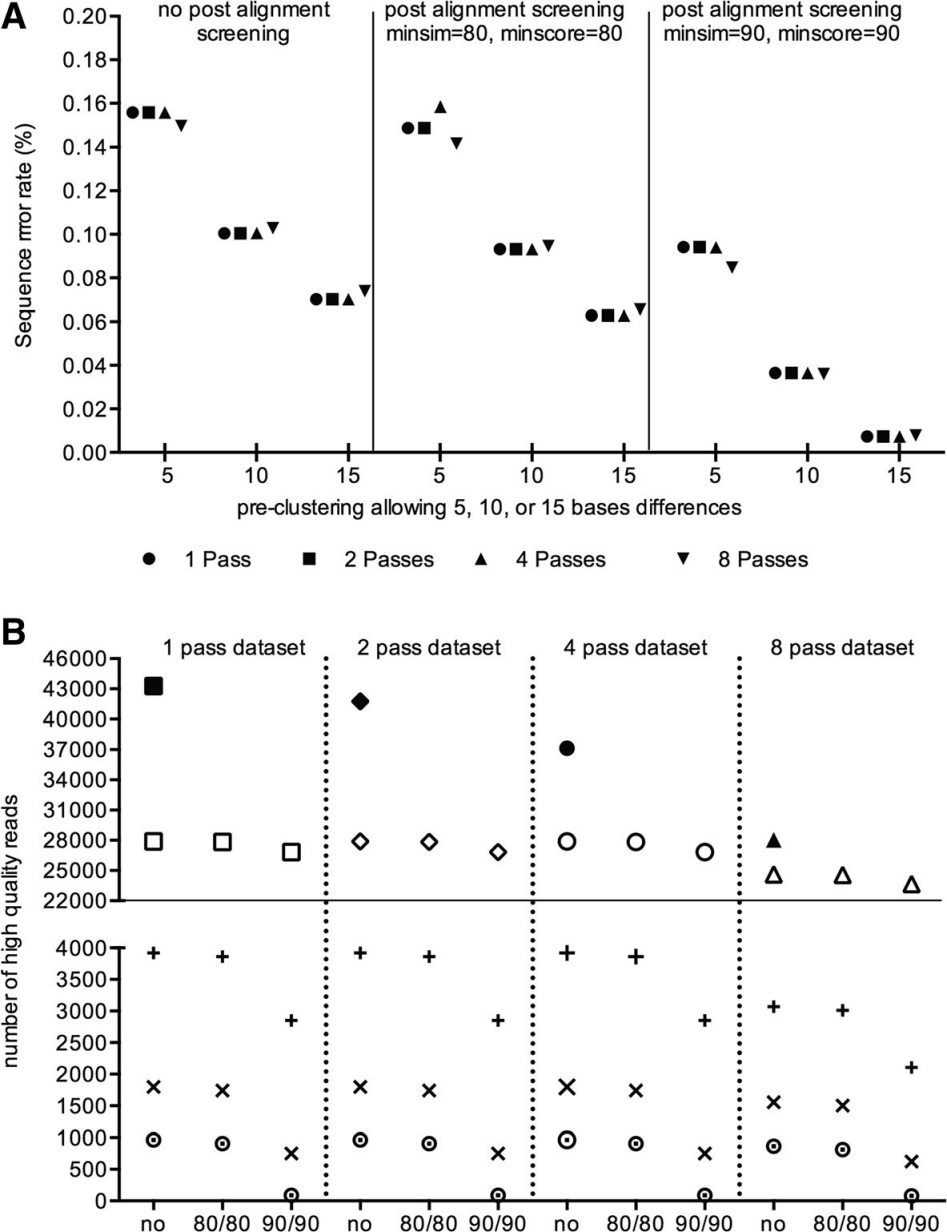
Change in sequence error rate **a** and the proportion of high quality reads that were retained when using different sequencing curation methods **b. a** The overall sequence error rates are plotted on the y-axis. The x-axis show the different allowed mismatches in the pre-clustering steps (5, 10, and 15 base pair for the full-length bacterial 16S rRNA gene) across the different screened datasets: no post alignment screening, post alignment screening using minimum sequence similarity (minsim) and minimum alignment score of (minscore) of 80%, post alignment screening using minsim and minscore of 90%. The round, square, top facing triangle and bottom facing triangle symbols denotes the 1 pass, 2 pass, 4 pass, and 8 pass datasets, respectively. **b** Top panel – Bold symbols, number of filtered raw reads across the different 1 pass, 2 pass, 4 pass, and 8 pass datasets. Top panel – Open symbols, number of high quality reads (HQRs) across the different post aligned screened datasets, i.e. no post alignment screening, post alignment screening using minimum sequence similarity and minimum alignment score of 80% (80/80), post alignment screening using minimum sequence similarity and minimum alignment score of 90% (90/90). **b** Bottom panel, + symbol = number of HQRs in the pre-clustered dataset with 5 allowed mismatches, x symbol = number of HQRs in the pre-clustered dataset with 10 allowed mismatches, *circle with dot* = number of HQRs in the pre-clustered datasets with 15 allowed mismatches

The PacBio consensus circular reads (CCS) were filtered for a minimum of 1 pass, 2 passes, 4 passes, and 8 passes using the PacBio SMRT portal. The number of raw reads, trimmed sequences (high quality reads (HQR)), OTU clusters at unique, 0.01, 0.02, 0.03, 0.04, and 0.05 distance level, and overall sequence error rates were calculated for the standard quality filtered datasets and for datasets with extra post alignment screening using a minimum alignment score of 80% or 90% and a minimum similarity score of 80% or 90% (Fig. 1 and Additional file 2: Table S2). The high number of OTUs obtained from the standard quality filtering across the different passes prompted us to implement post alignment screening in order to decrease the number of spurious OTUs and hence improve the sequence error rates. Theoretically, a single bacterial mock community should only generate one OTU at 97% sequence similarity. A 97% identity to partial-length bacterial 16S rRNA sequence is commonly accepted for species definition.

As expected, implementing the post alignment screening effectively reduces the sequence error rate (Fig. 1a). The low stringency post alignment screening (minsim = 80, minscore = 80) did not much improve the sequence error rate compared to the non-post alignment screening for either of the passes (1, 2, 4, or 8 passes) or different allowed pre-clustering mismatches (5, 10, or 15 bases). A dramatic improvement in sequence error rate was observed by increasing the allowed mismatches in the pre-clustering step. This was true for all three different post alignment screened datasets (no, minsim = 80, minscore = 80, and minsim = 90, minscore = 90). The lowest sequence error rate was 0.007% which was obtained from the high stringency post aligned screened dataset with 15 allowed mismatches in the pre-clustering step, followed by 0.035% for the same datasets but allowing for 10 mismatches in the pre-clustering step (Fig. 1a and Additional file 2: Table S2).

The improvement in sequence error rate was strongly associated with a reduction of high quality pre-clustered sequences. This was particularly pronounced in the high stringency (minsim = 90, minscore = 90) screened datasets (Fig. 1b, bottom panel).

As expected, an increase in passes (from ≥1 pass, ≥2 pass, ≥4 pass, and ≥8 pass) did decrease the number of raw reads obtained from the PacBio platform, from 43,265, to 41,756, to 37,136 and 28,042 reads, respectively (Fig. 1b top panel, bold symbols, Additional file 2: Table S2). However, the number of high quality sequences did not decrease accordingly. They were very similar across the 1 pass, 2 pass, and 4 pass datasets (open symbols in Figure B, top panel, Additional file 2: Table S2 in good aligned rows). The 8 pass dataset had the lowest number of high quality sequences which was between 24,612 and 23,653 compared to the 1 pass dataset which was between 27,903 and 26,834.

**Fig. 2:**
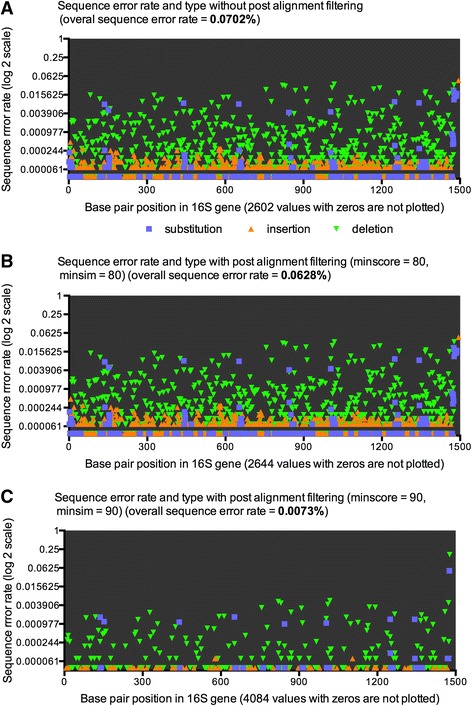
Comparing sequence error types across different alignment filtering. The different types of sequencing errors, obtained from the MOTHUR error report file, were plotted in Graph Pad Prism 6. The x-axis shows the base pair position in the full-length bacterial 16S rRNA gene and the y-axis shows the sequence error rate on a log 2 scale. The abundance of substitution errors, insertion errors, and deletion errors using standard quality filtering **a**, with low stringency post alignment filtering **b**, and high stringency post alignment filtering **c** are shown. The standard quality filtering parameters were: maximum ambiguous sequences = 0, maximum number of homo-polymers 6, sequence quality average of 35 over a sequence quality window of 50 bp. The extra post alignment screening parameters were: post alignment screening using minimum sequence similarity and minimum alignment score of 80% **b** and post alignment screening using minimum sequence similarity and minimum alignment score of 90% **c**

We plotted the different sequence error types from the 4-pass dataset with 15 allowed mismatches in the pre-clustering step, obtained from the “seq.error” step in Mothur in Graph Pad. Figure 2 shows that the largest proportion of sequence errors were deletions. The high stringency post alignment screening, using a minimum of 90% search score and a minimum of 90% similarity score to template sequences reduced the sequence error rate from 0.0702% to 0.0073% compared to the no post aligned screened dataset, which also resulted in a substantial reduction of all sequence error types, mostly deletion and insertion errors (Fig. 2a, c).

The low stringency post alignment screening using a minimum of 80% search score and a minimum of 80% similarity score had little effect in the overall reduction of the sequence error (Fig. 2b) compared to the standard quality filtered datasets with no post alignment screening (Fig. 2a). The slightly observed increase of the sequence error rate in the 8 pass dataset is a result of the lower total number sequences in this datasets. We also identified that substitution errors were not evenly distributed across the full-length bacterial 16S  rRNA gene but were detected at hotspots across the full-length bacterial 16 s rRNA gene (Fig. 2). All 27,901 high quality sequences of the no-post aligned screened 4 pass datasets with 15 allowed mismatches had a total of 25,323 sequence errors. Of these, 1,383 (5.46%) were insertions, 22,350 (88.26%) were deletions, and 1,590 (6.28%) were substitutions. The proportions of error types were very similar for the high stringency post aligned screened 4 pass datasets. The 26,832 high quality sequences had 1,554 sequence errors of which 89 (5.73%) were insertions, 1,312 (84.43%) were deletions, and 153 (9.85%) were substitutions.

The result of this “test of principle” using DNA from a single bacterial species was used to validate and optimize the bioinformatics pipeline for PacBio sequences.

Our quality filtering steps including (1) trimming by length (<1400 bp and >1600 bp), (2) removing homo polymers longer than 6 bp, (3) removing all sequences with ambiguous base pairs, (4) using a quality average score of 35 over a rolling quality window of 50 bp, all of which produces a very consistent number of high quality reads across the ≥1 pass, ≥2 pass, and ≥4 pass datasets. The ≥8 pass filtering did not decrease the sequence error rate any further, but further reduced the number of high quality reads. Therefore, we used the ≥4 pass filtering for the stool sample analysis and human vaginal microbiome sample analysis.

### Pooled stool microbiome analysis

The PacBio consensus circular reads were filtered for a minimum of 4 passes using the PacBio SMRT portal. A total of 20,118 high quality reads (54.6% of raw reads) were obtained using standard quality filtering. Using the low stringency post alignment screening (minscore = 80, minsim = 80) and high stringency post alignment screening (minscore = 90, minsim = 90) decreased the number of high quality reads by another 888, and 4863 reads, respectively. As expected we observed a higher number of OTUs in the low stringency post alignment screened datasets across the 97% (129 OTUs versus 33 OTUs), 98% (545 OTUs versus 36 OTUs) and unique OTU clusters (1513 OTUs versus 81 OTUs) (Table 3). The higher number of OTUs seen in the low stringency screened datasets compared to the high stringency screened datasets is most likely a result of the high number of deletion errors present in these datasets, as seen in Fig. 2b and c.

**Table 3:** Stool sample analysis

			Capillary Sequencing	PacBio	PacBio	PacBio	PacBio	PacBio	PacBio
				97%	98%	unique	97%	98%	unique
				OTU	OTU	OTU	OTU	OTU	OTU
				90/90	90/90	90/90	80/80	80/80	80/80
		No of OTUs	NA	33	36	81	129	545	1513
		No of species	40	32	33	33	39	46	48
		Shared with Capillary	NA	27	28	28	31	31	32
		Shared in %		67.50%	70%	70%	77.50%	77.50%	80%
Phyla	Genera	Species							
Actinobacteria	Bifidobacterium	adolscent	yes	yes	yes	yes	yes	yes	yes
Actinobacteria	Bifidobacterium	bifidum	yes	yes	yes	yes	yes	yes	yes
Actinobacteria	Bifidobacterium	pseudoca	yes	yes	yes	yes	yes	yes	yes
Actinobacteria	Collinsella	aerofaciens	yes	yes	yes	yes	yes	yes	yes
Bacterium	Bacterium	R 2	yes	yes	yes	yes	yes	yes	yes
Bacteroidetes	Bacteroides	uniformis	yes	yes	yes	yes	yes	yes	yes
Firmicutes	Clostridium	baratti	yes	yes	yes	yes	yes	yes	yes
Firmicutes	Clostridium	bartlettii	yes	yes	yes	yes	yes	yes	yes
Firmicutes	Clostridium	clostridiofo	yes	yes	yes	yes	yes	yes	yes
Firmicutes	Clostridium	disporicum	yes	yes	yes	yes	yes	yes	yes
Firmicutes	Clostridium	hathewayi	yes	yes	yes	yes	yes	yes	yes
Firmicutes	Clostridium	leptum	yes	yes	yes	yes	yes	yes	yes
Firmicutes	Clostridium	ramosum	yes	yes	yes	yes	yes	yes	yes
Firmicutes	Clostridium	aff. innocuum CM970	yes	yes	yes	yes	yes	yes	yes
Firmicutes	Coprococcus	comes	yes	yes	yes	yes	yes	yes	yes
Firmicutes	Butyricicoccus	pullicaecorum	yes	yes	yes	yes	yes	yes	yes
Firmicutes	Dorea	formicigenerans	yes	yes	yes	yes	yes	yes	yes
Firmicutes	Eubacterium	cylindroides	yes	yes	yes	yes	yes	yes	yes
Firmicutes	Eubacterium	eligens	yes	yes	yes	yes	yes	yes	yes
Firmicutes	Flavonifractor	plautii	yes	yes	yes	yes	yes	yes	yes
Firmicutes	Megasphaera	elsdenii	yes	yes	yes	yes	yes	yes	yes
Firmicutes	Mitsuokella	jalaludinii	yes	yes	yes	yes	yes	yes	yes
Firmicutes	Roseburia	faecis	yes	yes	yes	yes	yes	yes	yes
Firmicutes	Ruminococcus	gnavus	yes	yes	yes	yes	yes	yes	yes
Bacteroidetes	Parabacteroides	distasoni	yes	yes	yes	yes	yes	yes	yes
Firmicutes	Clostridium	symbiosum	yes	yes	yes	yes	yes	yes	yes
Firmicutes	Catenibacterium	mitsuokai	yes	yes	yes	yes	yes	yes	yes
Firmicutes	Roseburia	hominis	yes		yes	yes	yes	yes	yes
Bacterium	Bacterium	ic1340	yes				yes	yes	yes
Bacterium	Bacterium	New Zealand 4	yes				yes	yes	yes
Bacteroidetes	Alistipes	onderdonkii	yes				yes	yes	yes
Firmicutes	Ruminococcus	bromii	yes						yes
Bacteroidetes	Bacteroides	coprocola	yes						
Bacteroidetes	Prevotella	copri	yes						
Firmicutes	Clostridium	lituseburense	yes						
Firmicutes	Clostridium	xylanolyticus	yes						
Firmicutes	Coprococcus	eutactus	yes						
Firmicutes	Ruminococcus	albus	yes						
Firmicutes	Ruminococcus	flavefacien	yes						
Firmicutes	Turicibacter	sanguinis	yes						
Bacteroidetes	Alistipes	indistinctus		yes	yes	yes	yes	yes	yes
Firmicutes	Clostridium	stramisolven		yes	yes	yes	yes	yes	yes
Firmicutes	Eubacterium	Tenue		yes	yes	yes	yes	yes	yes
Firmicutes	Faecalibacterium	prausnitzi		yes	yes	yes	yes	yes	yes
Bacteroidetes	Bacteriodes	vulgatus		yes	yes	yes	yes	yes	
Bacteroidetes	Bacteroides	plebeius					yes	yes	yes
Firmicutes	Mitsuokella	multacida					yes	yes	yes
Firmicutes	Ruminococcus	torques					yes	yes	yes
Firmicutes	Christensenella	minuta				yes		yes	yes
Firmicutes	Clostridium	aminovalericum				yes		yes	yes
Firmicutes	Clostridium	bolteae						yes	yes
Firmicutes	Clostridium	disporicum						yes	yes
Firmicutes	Clostridium	spiroforme						yes	yes
Firmicutes	Blautia	coccoides						yes	yes
Firmicutes	Coprobacillus	cateniformis						yes	yes
Firmicutes	Lactobacillus	salivarius							yes
Firmicutes	Catabacter	hongkongensis							yes

*Abbreviations*: *90/90* minimum alignment and similarity score of 90% during post alignment screening, *80/80* minimum alignment and similarity score of 80% during post alignment screening, *No* number, *OTU* operational taxonomic units

Next, we profiled the predicted species using full-length bacterial 16S rRNA capillary sequence data from the pooled 50 stool colonies using the software package ARB and a customized version of the SILVA SSURef database (release 119) that was generated by removing environmental and uncultured taxa. This was compared with the species profile of the PacBio post alignment screened datasets from the 97%, 98% and unique OTUs cluster (Table 3). Note that the number of species identified is lower than the number of OTUs as ARB will often assign the same species identifier to more than one OTU.

Forty species were identified from the pool of 50 capillary sequenced stool colonies. These were four Actinobacteria species, five Bacteroidetes species, three Bacterium (unclassified), and 28 Firmicutes species (Table 3). 27 species (67.5%) were shared between capillary sequences and all six different PacBio filtered datasets, i.e. low and high stringency post alignment screening at 97%, 98% and unique OTU distance level (Table 3). Four additional species were shared with the capillary sequences and the PacBio low stringency post aligned screened dataset for all three OTU distance levels. One Firmicutes species (Ruminococcus bromii) was only shared between capillary sequences and the unique distance level from the low stringency post aligned screened datasets. This brings to a total of 80% shared species between the low post alignment screened datasets (unique distance level) and capillary sequences.

However, if we accept the higher number of shared species from the low stringency dataset, we would also have to accept a higher rate of false positive identified species. Between eight to 16 additional false positive species were identified from the low stringency post aligned screened dataset compared to three to four for the high stringency post aligned screened dataset. The higher number of OTUs from the standard quality filtering without post alignment screening, 2009 OTUs for the unique OTU distance level, 1001 OTUs for 98% OTU distance level, and 533 OTUs for the 97% OTU distance level, did not identify additional false positive species (data not shown). Eight species (two Bacteroidetes, and six Firmicutes) were only classified by capillary sequencing. This highlights that 20% of species (8/40) from the pooled stool colonies could not be classified by PacBio sequencing.

### Human vaginal microbiome analysis

Eleven human vaginal microbiome samples were sequenced with PacBio P6 chemistry and with Illumina MiSeq sequencing using the 600-cycle kit, which enabled the full-overlap paired-end sequencing of the bacterial 16S rRNA V1V2 gene region. The PacBio samples were barcoded with standard PacBio barcodes as outlined in Additional file 1: Table S1. For further analysis we used the 4 pass filtered datasets. The PacBio SMRT portal barcode filtering was able to assign 29,074 sequences to the eleven barcodes. We were able to increase the number of barcoded sequences by using MOTHUR software and the “fastq.info” function, which allows a user-defined barcode list and adjustable mismatches in the barcode sequence. Allowing four mismatches in the barcode sequence generated the same number of barcoded sequences as the PacBio SMRT portal (n = 29,074/58,951 raw sequences). The number of barcoded sequences could be increased to 34791, 35552, and 36098 reads by allowing one, two, and three mismatches in the barcode, respectively (this is because allowing 4 mismatches meant that some barcodes could not be uniquely assigned). For further analysis we used the filtered datasets with 2 allowed mismatches in the barcode. The MiSeq samples were part of a larger bacterial vaginosis study and were indexed with standard MiSeq indexing as outlined in the materials and methods section. The overall sequence error rate in the MiSeq data set was 0.00902%.

The total number of high quality reads and OTUs at the unique, 99%, 98% and 97% distance levels are shown in Table 1. The numbers of high quality reads in individual samples are shown in Additional file 3: Table S4. For PacBio we analyzed the full-length bacterial 16S rRNA gene and the *in silico* selected bacterial 16S rRNA V1V2 gene region, using the low stringency and high stringency post aligned screened datasets. This was compared with the MiSeq full-overlap paired-end sequenced bacterial 16S rRNA V1V2 gene region of the same sample cohort (Table 1). For PacBio, we observed a very large number of OTUs in the low stringency post aligned screened datasets. This was most obvious for the full-length Bacterial 16S rRNA gene at unique (1316 OTUs), 99% (1109 OTUs), and 98% (453 OTUs) distance level, compared to 209 OTUs, 90 OTUs, and 63 OTUs for the same distance level, respectively, using the high stringency post aligned screened datasets. The largest proportions of the additional OTUs in the low stringency datasets were singleton OTUs. The differences between the PacBio *in silico* selected bacterial 16S rRNA V1V2 gene region in regards to the observed number of OTUs between the two different post alignment screened datasets was much smaller. The difference was even smaller after removing the singleton OTUs (Table 1). We analyzed the MiSeq dataset with all sequences (*n* = 508,016; non-subsampled) and with a subsampled datasets of 27,596 sequences which was equivalent to the number of PacBio *in silico* selected bacterial 16S rRNA V1V2 gene region dataset from the low stringency post alignment screening. As expected the number of OTUs was higher by analyzing the non-subsampled MiSeq bacterial 16S rRNA V1V2 gene sequences. The number of singletons free OTUs in the subsampled MiSeq datasets was similar to the number of singletons free OTUs in the PacBio *in silico* selected bacterial 16S rRNA V1V2 gene datasets (Table 1).

### Alpha diversity analysis of the human vaginal microbiome samples

**Fig. 3:**
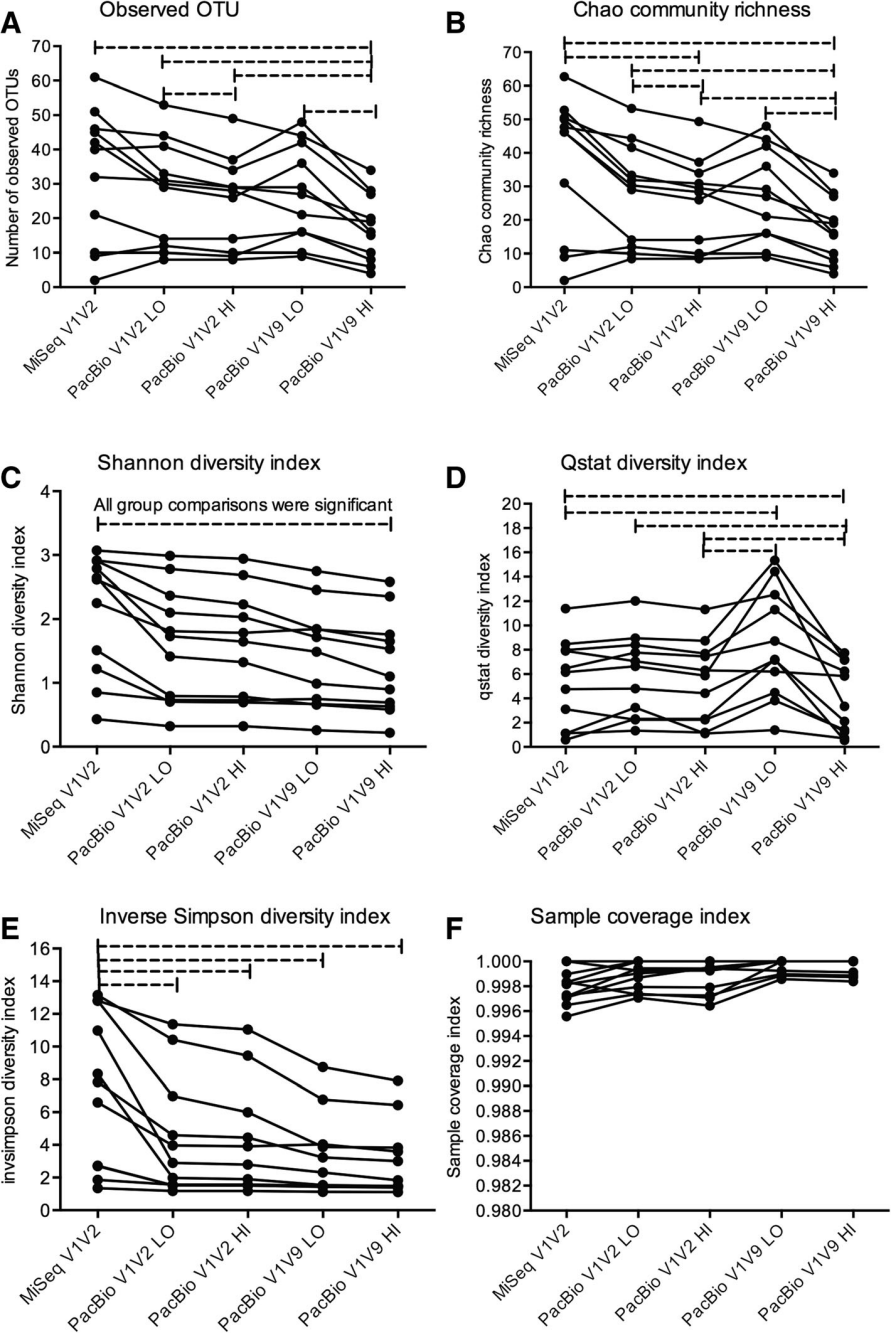
Alpha diversity analysis using unique OTU distance. Several different alpha diversity indexes: number of observed OTUs **a**; Chao community richness **b**; Shannon diversity index **c**; Q statistic – Qstat diversity index **d**; inverse Simpson diversity index **e**; and sample coverage index **f** calculated in Mothur are shown for five different datasets analyzed. Lines connect the same samples in each dataset. Statistical analysis was performed in Graph Pad using the One-way ANOVA for between group comparisons. Abbreviations: OTU = operational taxonomy unit, MiSeq = Illumina MiSeq sequencing platform, V1V2 = bacterial 16S rRNA gene region V1 to V2, V1V9 = bacterial full-length 16S rRNA gene, LO = PacBio datasets with low stringency post alignment screening using a minimum alignment score of 80% and minimum alignment similarity score of 80%. HI = PacBio datasets with high stringency post alignment screening using a minimum alignment score of 90% and a minimum alignment similarity score of 90%. Dashed line = significant difference at 95% confidence interval (*P* < 0.05)

**Fig. 4:**
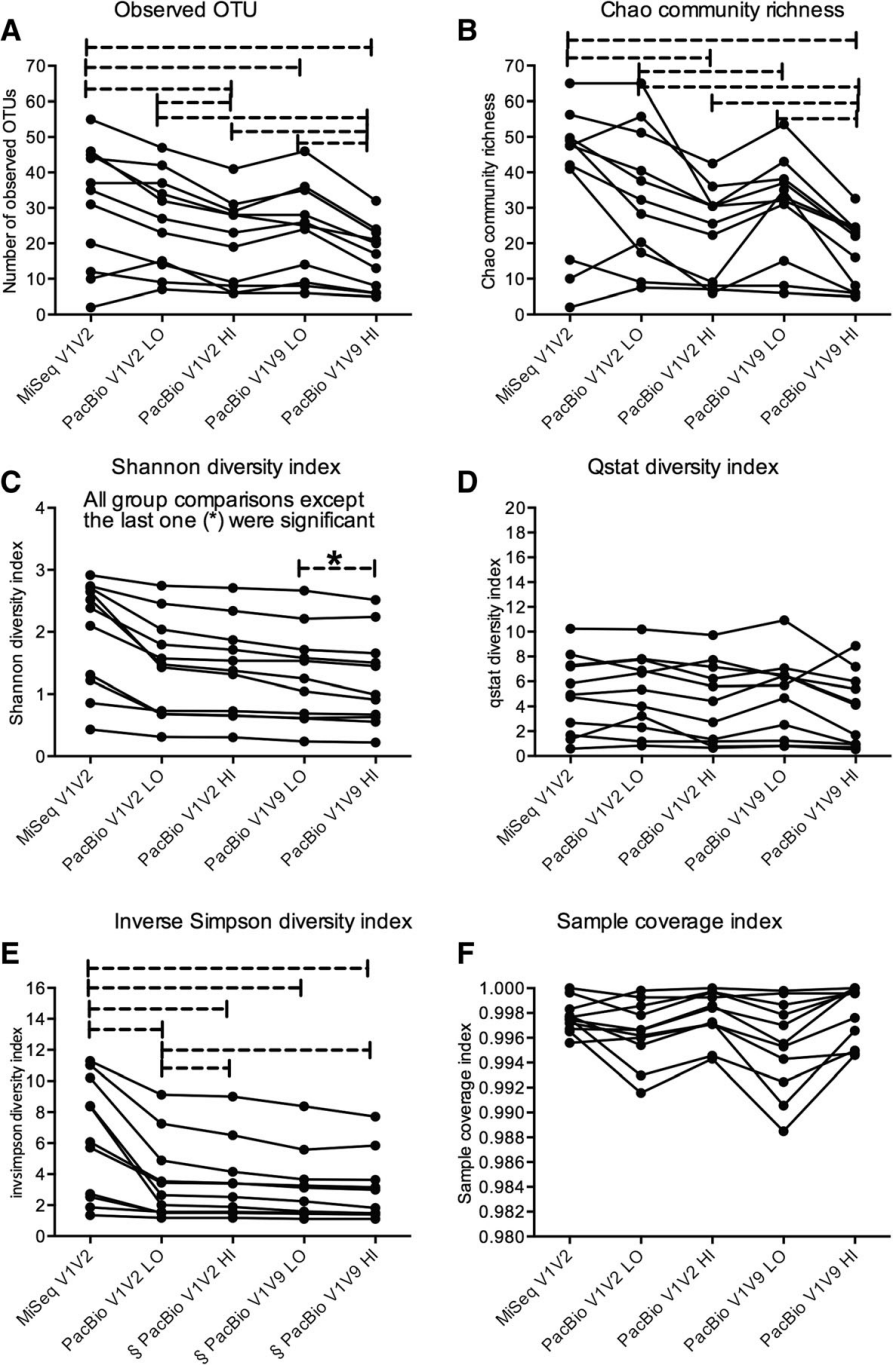
Alpha diversity analysis using 97% (0.03) OTU distance. Several different alpha diversity indexes: number of observed OTUs **a**; Chao community richness **b**; Shannon diversity index **c**; Q statistic – Qstat diversity index **d**; inverse Simpson diversity index **e**; and sample coverage index **f** calculated in Mothur are shown for five different datasets analyzed. Lines connect the same samples in each detection method. Statistical analysis was performed in Graph Pad using the One-way ANOVA for between group comparisons. Abbreviations: OTU = operational taxonomy unit, MiSeq = Illumina MiSeq sequencing platform, V1V2 = bacterial 16S rRNA gene region V1 to V2, V1V9 = bacterial full-length 16S rRNA gene, LO = PacBio datasets with low stringency post alignment screening using a minimum alignment score of 80% and minimum alignment similarity score of 80%. HI = PacBio datasets with high stringency post alignment screening using a minimum alignment score of 90% and a minimum alignment similarity score of 90%. Dashed line = significant difference at 95% confidence interval (*P* < 0.05). § = These datasets did not pass the normality test

Alpha diversity analysis was performed using One-Way ANOVA. The alpha diversity analysis of the unique OTU distance level is shown in Fig. 3 and the 97% OTU distance level is shown in Fig. 4. Dashed lines in Figs. 3 and 4 represent significant differences between the different datasets at 95% confidence interval (*p* < 0.05).

To test whether the datasets used for alpha diversity analysis followed a Gaussian distribution, we tested each dataset using the D’Agostino-Person omnibus normality test and the Shapiro-Wilk normality test. All datasets except the dataset used for the inverse Simpson diversity index at 97% (0.03) OTU distance level passed the normality test (data not shown).

The number of observed 97% OTUs was significantly higher in the MiSeq bacterial 16S rRNA V1V2 gene region dataset compared to PacBio full-length bacterial 16S rRNA gene dataset and PacBio in-silico bacterial 16S rRNA V1V2 gene region, high stringency post aligned screened dataset (Fig. 4a). For both distance levels (unique OTUs and 97% OTUs), we observed a significantly larger number of OTUs in the low stringency aligned screened PacBio datasets compare to the high stringency post aligned screened PacBio datasets (Figs. 3a and 4a). The Chao community richness estimation resulted in a similar number of OTUs compared to the observed OTUs (Fig. 3a and b for unique OTU distance and Fig. 4a and b for 97% OTU distance). We also observed similar significant differences for the Chao richness estimators and observed OTU between the different datasets. The Shannon diversity index was significantly different between all group comparisons except for the comparison of the low and high stringency screened PacBio full-length bacteria 16S  rRNA gene datasets at 97% OTU level (Figs. 3c and 4c). This was in contrast to the Qstat diversity index which revealed significant differences between the MiSeq bacterial 16S rRNA V1V2 gene region dataset and PacBio full-length bacterial 16S rRNA gene dataset for the unique OTUs (Fig. 3d) and no significant differences between groups using the 97% OTUs (Fig. 4d). The inverse Simpson diversity index was significantly higher in the MiSeq bacterial 16S rRNA V1V2 gene region data set compared to any of the PacBio datasets at the unique and 97% OTU distance level (Figs. 3e and 4e). Three out of five datasets used for the inverse Simpson diversity analysis at 97% distanced level (marked with “§” in Fig. 4e) did not pass the normality test. The same dataset was tested by the non-parametric Kruskal-Wallis test, which was not significant. The sampling coverage index was above 99.5% for all unique OTU datasets (Fig. 3f) and slightly less (min 98.8%) for all 97% OTU datasets (Fig. 4f).

### Bacterial species profiling

We also compared the bacterial taxonomic profile between the PacBio full-length bacterial 16S rRNA gene, PacBio *in silico* selected bacterial 16S rRNA V1V2 gene region and the MiSeq bacterial 16S rRNA V1V2 1gene region. For this we profiled the representative sequences of the singleton-free unique OTUs from the vaginal sequence dataset using the software package ARB and the customized version of the SILVA SSURef database (release 119). Seventy species from 129 OTUs were identified using the MiSeq bacterial 16S rRNA V1V2 gene region protocol (Table 4). When compared with low stringency post aligned screened PacBio *in silico* selected bacterial 16S rRNA V1V2 gene region dataset and PacBio full-length bacterial 16S rRNA gene dataset, we detected 39 shared species (58%) and 28 shared species (54%), respectively. The number of shared species decreased to 26 (37%) using the PacBio high stringency post aligned screened dataset.

**Table 4:** Vaginal microbiome species profiling

	Singleton-free unique OTU and no. of species
MiSeq V1V2	*129 OTUs = 70 species*
Subsampled to 27596	
PacBio in silico V1V2	*154 OTUs = 63 species*
Minscore = 80 Minsim = 80	(39 species (58%) shared with MiSeq V1V2)
PacBio in silico V1V2	*131 OTUs = 48 species*
Minscore = 90 Minsim = 90	(26 species (37%) shared with MiSeq V1V2)
PacBio V1V9	*261 OTUs = 52 species*
Minscore = 80 minsim = 80	(28 species (40%) shared with MiSeq V1V2)
	(32 species (62%) shared with PacBio in silico V1V2, minscore = 80, minsim = 80)
PacBio V1V9	*148 OTUs = 48 species*
Minscore = 90 minsim = 90	(26 species (37%) shared with MiSeq V1V2)
	(48 species (100%) shared with PacBio in silico V1V2, minscore = 90, minsim = 90)

*Abbreviations*: *minscore* minimum alignment score during post alignment screening, *minsim* minimum similarity score during post alignment screening, *OTU* operational taxonomic units, *MiSeq* Illumina MiSeq sequencing, *V1V2* bacterial 16S rRNA V1V2 gene region, *V1V9* full-length bacterial 16S rRNA gene

## Discussion

This study aimed to validate the value of full-length bacterial 16S rRNA gene sequencing using PacBio RS II platform and to compare with capillary sequencing and short-read Illumina MiSeq sequencing for bacterial species classification.

We used the PacBio full-length bacterial 16S rRNA gene sequence data of a *Staphylococcus aureus* lab strain for bioinformatics pipeline validation and for pipeline optimization. For our PacBio sequence quality filtering we introduced two different post alignments screening with an 80% minimum alignment and similarity score (low stringency screening) or 90% minimum alignment and similarity score (high stringency screening). The implementation of a post alignment screening did effectively decrease the number of pre-clustered sequences. This was particular dominant for high stringency screening for all three different pre-clustering parameters (i.e. allowing 5, 10, or 15 bases mismatches) (Fig. 1b, bottom panel).

This resulted in a dramatic decrease in the number of OTUs, particularly at 98% (0.02) and 97% (0.03) distance level (Additional file 2).

This large decrease in OTUs, was most likely a consequence of removing large numbers of singleton OTUs originating from PacBio sequencing errors. Our implemented quality filtering steps removed low quality reads very consistently from the four different PacBio filtered ≥1 pass, ≥2 pass, ≥4 pass and ≥8 pass datasets. Interestingly, the sequence quality filtering resulted in very similar numbers of high quality reads from all four different filtered datasets despite large variations in the number of raw reads between the ≥1 pass (43268) and ≥8 pass (28042) datasets (Fig. 1b, top panel). This emphasizes that a good quality filtering step is of great importance and that an increase in the minimum number of passes for PacBio CCS reads did not generate a larger number of high quality reads after using our applied quality filtering parameters. We further analyzed the effect of different post alignment screening and different allowed mismatches during the pre-clustering step on overall sequence error rates and sequence error types. As expected the sequence error rate decreased depending on the allowed mismatches during the pre-cluster step. In the no post aligned screened dataset, the overall sequence error rate decreased from 0.15%, to 0.10%, and to 0.07% allowing 5, 10, or 15 bases mismatches during the pre-clustering (Fig. 1a). Schloss et al reported an overall sequence error rate of 0.027% allowing 15 bases mismatches during the pre-clustering [13]. The use of a different mock community and different laboratory/sequencing facility might have contributed to the different outcomes. Low stringency post alignment screening did not much improve the sequence error rate, but the high stringency post alignment screening has a dramatic improvement in the overall sequence error rate. We achieved a sequence error rate of 0.0365% and 0.00732% by allowing 10 and 15 mismatches, respectively, in the pre-clustering step. This was achieved for the 1 pass, 2 pass, and 4 pass dataset. The 0.0073% sequence error rate was smaller compared to the 0.00902% MiSeq error in this study. Within a given dataset and the same allowed pre-clustered mismatches, the number of passes did not improve the sequence error rate: i.e. the sequence error rate was very similar for the ≥1 pass, ≥2 pass, ≥4 pass, and ≥8 pass datasets (Fig. 1a and Additional file 2: Table S2). This was in contrast to the Schloss study, which reported a reduced error rate by 8.48 to 37.08% with at least 10-fold coverage (10 passes) compared to those with less coverage.

We have identified different proportions of sequence error types compared to the Schloss study. Our highest error types were deletions, which accounted for over 80% of all errors. The most abundant error types in the Schloss study were substitutions, which accounted for 50.9% of all errors, followed by 31.2% for insertions and 17.9% for deletions. Other groups have reported insertions and deletions as the most abundance error types in PacBio sequence data [12, 24, 25]. Singer et al [12] also reported that substitutions errors are more difficult to correct than insertion and deletion errors. We could confirm the difficulty of removal of substitution errors, which were still present at the different hotspots after applying the high stringency post alignment screening which otherwise reduced the overall sequence error rate to an excellent range of 0.0073%.

We used the pooled stool clone sequence dataset to investigate the effect of low and high stringency post alignment screening on species classification by comparing 97% (0.03), 98% (0.02), and unique OTU distance level from the 4 pass PacBio dataset. If we still consider the Sanger capillary sequencing of the full-length bacterial 16S as the gold standard for species identification, then the discrepancy in species detection between PacBio sequence data and capillary sequencing indicates that the sequencing error rates in PacBio consensus circular reads can lead to additional classification of false positive species. The larger number of shared species between capillary sequence data and the low stringent screened sequence data comes at the expense of identifying a larger proportion of false positive species. The larger number of false positive species in the low stringency post alignment screened dataset is a reflection of the higher number of OTUs obtained using the low stringency screening parameters (Table 3). We could not identify a bias regarding different phyla and genera classification between capillary and PacBio full-length bacterial 16S rRNA gene in the pooled stool community. Different species of the same genus were identified by capillary sequencing only, which were not detected by PacBio sequencing and vice versa (Table 3). This highlights that species identification using bacterial 16S rRNA gene is a difficult process and should be conducted and concluded with great care.

In our human vaginal microbiome analysis we pooled 11 barcoded samples in one PacBio run and the coverage index showed good sampling coverage across all 11 samples. The similarity between species detected by MiSeq sequence data and PacBio sequence data was less consistent, despite a similar number of singletons free OTUs detected by the two platforms (not considering the non subsampled MiSeq dataset) (Table 1). Interestingly, the largest numbers of species were identified from the MiSeq bacterial 16S rRNA V1V2 gene region datasets, despite containing the lowest number of OTUs compared to any of the PacBio datasets (Table 4). From the PacBio low stringency screened in silico bacterial 16S rRNA V1V2 gene region dataset, 39/63 species (58%) were shared with the 70 MiSeq species, whereas from the high stringent screened in silico bacterial 16S rRNA V1V2 gene region dataset 26/48 species (37%) were shared with the 70 MiSeq species. The smaller number of OTUs and consequently smaller proportion of MiSeq shared species from the PacBio high stringency post aligned screened datasets (37% shared species) suggests that high stringency screening removed additional true positive OTUs and hence species from the dataset. On the other hand, low stringency post alignment screening resulted in an additional detection of 24 species compared to 22 species from the high stringency post alignment screening, which were not detected in the MiSeq dataset. The same pattern of additional false positive species using low stringency screening was observed in the stool colony study.

The increased species diversity from the PacBio *in silico* selected bacterial 16S rRNA V1V2 gene region and MiSeq bacterial 16S rRNA V1V2 gene region datasets reflects that analysis of partial bacterial 16S rRNA gene regions may overestimate bacterial diversity compare to the full-length bacterial 16S rRNA gene. A previous study reported a similar discrepancy in the number of OTUs between partial bacterial 16S rRNA gene regions and full length bacterial 16S rRNA gene [26].

Our observed discrepancy between PacBio full-length bacterial 16S rRNA gene dataset and shorter *in silico* PacBio bacterial 16S rRNA V1V2 gene region dataset is in similar accordance with a study conducted by Singer et al. [12]. In their Sakinaw Lake community dataset they could classify 74.5% species from the full-length bacterial 16S rRNA gene dataset and 49.4% of species from the in silico bacterial 16S rRNA V4 gene region dataset.

The fact that the forward PCR primer for the full-length bacterial 16S rRNA gene differed by two nucleotides from the PCR primer for the bacterial 16S rRNA V1V2 gene region (see sections 2.1 and 2.3) and the obvious different reverse PCR primers may also contributed to different taxonomic profiling. It has been recently reported that PCR primer choice influences bacterial 16S rRNA gene-based profiling, and that this was particularly dominant in the detection of *Bifidobacterium* in the human infant gut microbiome with slightly different 27f forward primer composition [27].

Koren et al. [28] demonstrated that the clustering of enterotypes across different body habitats is difficult to establish and to interpret. The same group also compared the efficiency of enterotype clustering using different bacterial 16S rRNA gene regions on fecal samples from the human microbiome project. Depending on the clustering method used they detected different outcomes for bacterial 16S rRNA V1V3 gene region and V3V5 gene region. No studies are available yet, which have investigated the enterotype clustering across different body habitats using full-length bacterial 16S rRNA gene data. However, using full-length bacterial 16S rRNA gene data might provide a good method for consistent enterotype clustering for multimodal distributions of sample abundances in the case of vaginal sequence data.

## Conclusions

In this study we compared (1) capillary full-length bacterial 16S rRNA gene sequencing with PacBio full-length bacterial 16S rRNA gene sequencing using a pool of stool colonies, and (2) PacBio full-length bacterial 16S rRNA gene sequencing and PacBio *in silico* generate bacterial 16S rRNA V1V2 gene region with MiSeq bacterial 16S rRNA V1V2 gene region using human vaginal samples.

Our implemented post alignment screening dramatically improved the sequence error rate. However, high stringency post alignment screening can remove true positive species, whereas low stringency quality filtering can result in a higher number of false positive species. Thus, a fine balance in sequence quality filtering is necessary to achieve the best outcome in using PacBio technology for full-length bacterial 16S rRNA gene sequencing. The taxonomic profiling effectiveness between different bacterial 16S rRNA gene regions and full-length bacterial 16S rRNA gene needs further validation, to determine whether the full-length bacterial 16S rRNA gene will provide better bacterial classification across different sample types. A true advantage of sequencing the full-length bacterial 16S rRNA gene would be the possible global comparison of microbiome studies, which currently can only be done with great difficulty due to different bacterial 16S rRNA gene regions used in different studies.

Current bacterial 16S rRNA gene studies can include several hundreds to thousands of samples, which can be readily analyzed by high throughput MiSeq indexing using up to several hundreds of samples per library. Depending on bimodal (e.g., gut) or multimodal (e.g., vagina) distribution of sample abundances, we would not recommend to pool more than 10 samples for good data differentiation on the current PacBio platform. However, the latest model of the PacBio machine “the Sequel System” should be able to deliver about seven times more reads then the current available PacBio instrument (http://www.pacb.com/products-and-services/pacbio-systems/sequel/). This would make the use of PacBio for full-length bacterial 16S rRNA sequencing more attractive for larger datasets.

## Additional files

Additional file 1: Table S1. Outlines the barcoded PacBio PCR-primers for the full-length bacterial 16S rRNA gene. (DOC 36 kb)
Additional file 2: Table S2. Outlines raw reads, high quality trimmed sequences, high quality trimmed unique sequences, number of OTUs and overall sequence error rates for detailed Staphylococcus aureus analysis, across different parameters for post alignment screening (none, low (minsim = 80, minscore = 80), and high (minsim = 90, minscore = 90)) and different allowed mismatches during the pre-clustering step (5, 10, or 15). (DOC 100 kb)
Additional file 3: Table S4. Shows the numbers of high quality reads in individual vaginal samples across different sequencing platform analysis methods. (DOC 45 kb)
Additional file 4: Shows the capillary sequence data from 50 stool colonies, which were pooled for PacBio sequencing. (DOC 112 kb)

## Abbreviations

CCS: Circular consensus sequencing
DNA: Deoxyribonucleic acid
dNTPs: Deoxyribose nucleoside triphosphate
OTUs: Operational taxonomic units
PacBio: Pacific biosciences
PCR: Polymerase chain reaction
SMRT: Single molecule, real-time sequencing
