# Supplementary material for: Evaluation of PacBio sequencing for full-length bacterial 16S rRNA gene classification
Source: BMC Microbiol. 2016 Nov 14;16:274. doi: 10.1186/s12866-016-0891-4 (PMC5109829; doi:10.1186/s12866-016-0891-4)
Supplement: Additional file 1: Table S1. — Outlines the barcoded PacBio PCR-primers for the full-length bacterial 16S rRNA gene. (DOC 36 kb) [file 12866_2016_891_MOESM1_ESM.doc]

**Supplementary Table 1.** Barcoded PCR-primers for PacBio sequencing (full length (V1V9) bacterial 16s rRNA gene)

PRIMER NAME BARCODE SEQUENCE PRIMER SEQUENCE

BACT16s_v1_0001_Forward TCAGACGATGCGTCATAGMGTTYGATYMTGGCTCAG

BACT16s_v1_0002_Forward CTATACATGACTCTGCAGMGTTYGATYMTGGCTCAG

BACT16s_v1_0003_Forward TACTAGAGTAGCACTCAGMGTTYGATYMTGGCTCAG

BACT16s_v1_0004_Forward TGTGTATCAGTACATGAGMGTTYGATYMTGGCTCAG

BACT16s_v1_0005_Forward ACACGCATGACACACTAGMGTTYGATYMTGGCTCAG

BACT16s_v1_0006_Forward GATCTCTACTATATGCAGMGTTYGATYMTGGCTCAG

BACT16s_v1_0007_Forward ACAGTCTATACTGCTGAGMGTTYGATYMTGGCTCAG

BACT16s_v1_0008_Forward ATGATGTGCTACATCTAGMGTTYGATYMTGGCTCAG

BACT16s_v1_0009_Forward CTGCGTGCTCTACGACAGMGTTYGATYMTGGCTCAG

BACT16s_v1_0010_Forward GCGCGATACGATGACTAGMGTTYGATYMTGGCTCAG

BACT16s_v1_0011_Forward CGCGCTCAGCTGATCGAGMGTTYGATYMTGGCTCAG

BACT16s_v1_0012_Forward GCGCACGCACTACAGAAGMGTTYGATYMTGGCTCAG

BACT16s_v1_0013_Forward ACACTGACGTCGCGACAGMGTTYGATYMTGGCTCAG

BACT16s_v1_0014_Forward CGTCTATATACGTATAAGMGTTYGATYMTGGCTCAG

BACT16s_v1_0015_Forward ATAGAGACTCAGAGCTAGMGTTYGATYMTGGCTCAG

BACT16s_v1_0016_Forward TAGATGCGAGAGTAGAAGMGTTYGATYMTGGCTCAG

BACT16s_v1_0017_Forward CATAGCGACTATCGTGAGMGTTYGATYMTGGCTCAG

BACT16s_v1_0018_Forward CATCACTACGCTAGATAGMGTTYGATYMTGGCTCAG

BACT16s_v1_0019_Forward CGCATCTGTGCATGCAAGMGTTYGATYMTGGCTCAG

BACT16s_v1_0020_Forward TATGTGATCGTCTCTCAGMGTTYGATYMTGGCTCAG

BACT16s_v9_0001_Reverse ATGACGCATCGTCTGAACGGYTACCTTGTTACGACTT

BACT16s_v9_0002_Reverse GCAGAGTCATGTATAGACGGYTACCTTGTTACGACTT

BACT16s_v9_0003_Reverse GAGTGCTACTCTAGTAACGGYTACCTTGTTACGACTT

BACT16s_v9_0004_Reverse CATGTACTGATACACAACGGYTACCTTGTTACGACTT

BACT16s_v9_0005_Reverse AGTGTGTCATGCGTGTACGGYTACCTTGTTACGACTT

BACT16s_v9_0006_Reverse GCATATAGTAGAGATCACGGYTACCTTGTTACGACTT

BACT16s_v9_0007_Reverse CAGCAGTATAGACTGTACGGYTACCTTGTTACGACTT

BACT16s_v9_0008_Reverse AGATGTAGCACATCATACGGYTACCTTGTTACGACTT

BACT16s_v9_0009_Reverse GTCGTAGAGCACGCAGACGGYTACCTTGTTACGACTT

BACT16s_v9_0010_Reverse AGTCATCGTATCGCGCACGGYTACCTTGTTACGACTT

BACT16s_v9_0011_Reverse CGATCAGCTGAGCGCGACGGYTACCTTGTTACGACTT

BACT16s_v9_0012_Reverse TCTGTAGTGCGTGCGCACGGYTACCTTGTTACGACTT

BACT16s_v9_0013_Reverse GTCGCGACGTCAGTGTACGGYTACCTTGTTACGACTT

BACT16s_v9_0014_Reverse TATACGTATATAGACGACGGYTACCTTGTTACGACTT

BACT16s_v9_0015_Reverse AGCTCTGAGTCTCTATACGGYTACCTTGTTACGACTT

BACT16s_v9_0016_Reverse TCTACTCTCGCATCTAACGGYTACCTTGTTACGACTT

BACT16s_v9_0017_Reverse CACGATAGTCGCTATGACGGYTACCTTGTTACGACTT

BACT16s_v9_0018_Reverse ATCTAGCGTAGTGATGACGGYTACCTTGTTACGACTT

BACT16s_v9_0019_Reverse TGCATGCACAGATGCGACGGYTACCTTGTTACGACTT

BACT16s_v9_0020_Reverse GAGAGACGATCACATAACGGYTACCTTGTTACGACTT

The grey shadow sequence represents the barcode for each primer
