# Supplementary material for: Evaluation of PacBio sequencing for full-length bacterial 16S rRNA gene classification
Source: BMC Microbiol. 2016 Nov 14;16:274. doi: 10.1186/s12866-016-0891-4 (PMC5109829; doi:10.1186/s12866-016-0891-4)
Supplement: Additional file 2: Table S2. — Outlines raw reads, high quality trimmed sequences, high quality trimmed unique sequences, number of OTUs and overall sequence error rates for detailed Staphylococcus aureus analysis, across different parameters for post alignment screening (none, low (minsim = 80, minscore = 80), and high (minsim = 90, minscore = 90)) and different allowed mismatches during the pre-clustering step (5, 10, or 15). (DOC 100 kb) [file 12866_2016_891_MOESM2_ESM.doc]

**Supplementary Table 2:** Sequence reads, number of OTUs and overall sequence error rates for detailed *Staphylococcus aureus* analysis

| **1 pass dataset** | | |  | | | | | | | |
| --- | --- | --- | --- | --- | --- | --- | --- | --- | --- | --- |
| Total raw sequences | 43268 | |  | | | | | | | |
| Trimmed.sequences | 28340 | |  | | | | | | | |
| Trimmed unique sequences | 21749 | |  | | | | | | | |
| Minimum alignment score | none | none | | none | 80 | 80 | 80 | 90 | 90 | 90 |
| Minimum similarity score | none | none | | none | 80 | 80 | 80 | 90 | 90 | 90 |
| Pre.cluster differences | 5 | 10 | | 15 | 5 | 10 | 15 | 5 | 10 | 15 |
| Good aligned unique  Good aligned (total) | 12115 (27903) | 12115 (27903) | | 12115 (27903) | 12059 (27847) | 12059 (27847 | 12059 (27847) | 11046 (26834) | 11046 (26834) | 11046 (26834) |
| Pre.cluster | 3920 | 1799 | | 964 | 3864 | 1743 | 908 | 2851 | 752 | 89 |
| OTUs unique | 3920 | 1799 | | 964 | 3864 | 1743 | 908 | 2851 | 752 | 89 |
| 0.01 | 1394 | 1387 | |  | 1338 | 1331 |  | 415 | 412 |  |
| 0.02 | 371 | 395 | | 406 | 315 | 339 | 350 | 3 | 3 | 8 |
| **0.03** | **95** | **108** | | **123** | **43** | **57** | **72** | **2** | **2** | **2** |
| 0.04 | 23 | 30 | | 34 | 1 | 4 | 5 | 1 | 1 | 1 |
| 0.05 | 2 | 2 | | 3 |  | 1 | 1 |  |  |  |
| Overall sequence error rate %: | **0.15597** | **0.10053** | | **0.07022** | **0.14882** | **0.09325** | **0.06287** | **0.09410** | **0.03649** | **0.00731** |
| **2 passes dataset** | | |  | | | | | | | |
| Total raw sequences | 41756 | |  | | | | | | | |
| Trimmed.sequences | 28340 | |  | | | | | | | |
| Trimmed unique sequences | 21749 | |  | | | | | | | |
| Minimum alignment score | none | none | | none | 80 | 80 | 80 | 90 | 90 | 90 |
| Minimum similarity score | none | none | | none | 80 | 80 | 80 | 90 | 90 | 90 |
| Pre.cluster differences | 5 | 10 | | 15 | 5 | 10 | 15 | 5 | 10 | 15 |
| Good aligned unique  Good aligned (total) | 12115 (27903) | 12115 (27903) | | 12115 (27903) | 12059 (27847) | 12059 (27847) | 12059 (27847) | 11046 (26834) | 11046 (26834) | 11046 (26834) |
| % Of high total HQRs | 66.8239295 | 66.8239295 | | 66.8239295 | 66.68981703 | 66.68981703 | 66.68981703 | 64.26381837 | 64.26381837 | 64.26381837 |
| Pre.cluster | 3920 | 1799 | | 964 | 3864 | 1743 | 908 | 2851 | 752 | 89 |
| OTUs unique | 3920 | 1799 | | 964 | 3864 | 1743 | 908 | 2851 | 752 | 89 |
| 0.01 | 1394 | 1387 | |  | 1338 | 1331 |  | 415 | 412 |  |
| 0.02 | 371 | 395 | | 406 | 315 | 339 | 350 | 3 | 3 | 8 |
| **0.03** | **95** | **108** | | **123** | **43** | **57** | **72** | **2** | **2** | **2** |
| 0.04 | 23 | 30 | | 34 | 1 | 4 | 5 | 1 | 1 | 1 |
| 0.05 | 2 | 2 | | 3 |  | 1 | 1 |  |  |  |
| Overall sequence error rate %: | **0.15599** | **0.10053** | | **0.07022** | **0.14882** | **0.09325** | **0.06287** | **0.09410** | **0.03649** | **0.00731** |
| **4 passes dataset** | | |  | | | | | | | |
| Total raw sequences | 37136 | |  | | | | | | | |
| Trimmed.sequences | 28336 | |  | | | | | | | |
| Trimmed unique sequences | 21745 | |  | | | | | | | |
| Minimum alignment score | none | none | | none | 80 | 80 | 80 | 90 | 90 | 90 |
| Minimum similarity score | none | none | | none | 80 | 80 | 80 | 90 | 90 | 90 |
| Pre.cluster differences | 5 | 10 | | 15 | 5 | 10 | 15 | 5 | 10 | 15 |
| Good aligned unique  Good aligned (total) | 12113 (27901) | 12113 (27901) | | 12113 (27901) | 12057 (27845) | 12057 (27845) | 12057 (27845) | 11044 (26832) | 11044 (26832) | 11044 (26832) |
| Pre.cluster | 3920 | 1799 | | 964 | 3864 | 1743 | 908 | 2851 | 752 | 89 |
| OTUs unique | 3920 | 1799 | | 964 | 3864 | 1743 | 908 | 2851 | 752 | 89 |
| 0.01 | 1392 | 1387 | |  | 1336 | 1331 |  | 422 | 412 |  |
| 0.02 | 369 | 395 | | 406 | 313 | 339 | 350 | 3 | 3 | 8 |
| **0.03** | **95** | **108** | | **123** | **43** | **57** | **72** | **2** | **2** | **2** |
| 0.04 | 23 | 30 | | 34 | 1 | 4 | 5 | 1 | 1 | 1 |
| 0.05 | 2 | 2 | | 3 |  | 1 | 1 |  |  |  |
| Overall sequence error rate %: | **0.15599** | **0.10054** | | **0.07022** | **0.1586** | **0.09325** | **0.06288** | **0.09409** | **0.03649** | **0.00731** |
| **8 passes dataset** | | |  | | | | | | | |
| Total raw sequences | 28042 | |  | | | | | | | |
| Trimmed.sequences | 24973 | |  | | | | | | | |
| Trimmed unique sequences | 18669 | |  | | | | | | | |
| Minimum alignment score | none | none | | none | 80 | 80 | 80 | 90 | 90 | 90 |
| Minimum similarity score | none | none | | none | 80 | 80 | 80 | 90 | 90 | 90 |
| Pre.cluster differences | 5 | 10 | | 15 | 5 | 10 | 15 | 5 | 10 | 15 |
| Good aligned unique  Good aligned (total) | 9615 (24612) | 9615 (24612) | | 9615 (24612) | 9559 (24556) | 9559 (24556) | 9559 (24556) | 8656 (23653) | 8656 (23653) | 8656 (23653) |
| Pre.cluster | 3067 | 1558 | | 867 | 3011 | 1502 | 811 | 2108 | 622 | 79 |
| OTUs unique | 3067 | 1558 | | 867 | 3011 | 1502 | 811 | 2108 | 622 | 79 |
| 0.01 | 1189 | 1189 | |  | 1133 | 1133 |  | 318 | 311 |  |
| 0.02 | 338 | 365 | | 370 | 282 | 309 | 314 | 2 | 3 | 6 |
| **0.03** | **92** | **104** | | **116** | **40** | **53** | **65** |  | **2** | **2** |
| 0.04 | 22 | 28 | | 34 | 2 | 4 | 7 | 1 | 1 | 1 |
| 0.05 | 2 | 2 | | 3 | 1 | 1 | 1 |  |  |  |
| Overall sequence error rate %: | **0.14981** | **0.10288** | | **0.07392** | **0.14166** | **0.09463** | **0.06559** | **0.08478** | **0.03599** | **0.00778** |
