# Supplementary material for: Evaluation of PacBio sequencing for full-length bacterial 16S rRNA gene classification
Source: BMC Microbiol. 2016 Nov 14;16:274. doi: 10.1186/s12866-016-0891-4 (PMC5109829; doi:10.1186/s12866-016-0891-4)
Supplement: Additional file 3: Table S4. — Shows the numbers of high quality reads in individual vaginal samples across different sequencing platform analysis methods. (DOC 45 kb) [file 12866_2016_891_MOESM3_ESM.doc]

**Supplementary Table 4.** Numbers of high quality reads in individual vaginal samples across different analysis methods

| Sample Names | PacBio V1V9 | PacBio V1V9 | PacBio in silico V1V2 | PacBio in silico V1V2 | MiSeq V1V2 | MiSeq V1V2 |
| --- | --- | --- | --- | --- | --- | --- |
|  | 90/90 | 80/80 | 90/90 | 80/80 | Subsampled | Not subsampled |
|  | No. seqs | No. seqs | No. seqs | No. seqs | No. seqs | No. seqs |
| BBY15B | 796 | 929 | 1103 | 1140 | 2416 | 44398 |
| BBY15C | 2603 | 2967 | 3186 | 3216 | 2827 | 51504 |
| BBY15E | 1303 | 1789 | 2165 | 2380 | 2429 | 45300 |
| BBY15F | 1252 | 1488 | 1718 | 1756 | 2039 | 36453 |
| BBY15G | 3078 | 3263 | 3466 | 3498 | 2992 | 55494 |
| BBY15I | 878 | 1059 | 1461 | 1526 | 2227 | 41495 |
| BBY16L | 4470 | 4665 | 5024 | 5043 | 2941 | 56088 |
| BBY16M | 741 | 869 | 1054 | 1186 | 2002 | 36091 |
| BBY16N | 2379 | 2496 | 2697 | 2707 | 2444 | 44898 |
| BBY16X | 2307 | 2675 | 3127 | 3237 | 2729 | 49183 |
| BBY16Y | 1140 | 1403 | 1742 | 1828 | 2499 | 47112 |

V1V9 = full-length bacterial 16S rRNA gene, V1V2 = bacterial 16S rRNA V1V2 gene region, 90/90 = post alignment screening using minimum sequence similarity and minimum alignment score of 90%, 80/80 = post alignment screening using minimum sequence similarity and minimum alignment score of 80%, nseqs = number of sequences
