# Supplementary material for: Evaluation of PacBio sequencing for full-length bacterial 16S rRNA gene classification
Source: BMC Microbiol. 2016 Nov 14;16:274. doi: 10.1186/s12866-016-0891-4 (PMC5109829; doi:10.1186/s12866-016-0891-4)
Supplement: Additional file 4: — Shows the capillary sequence data from 50 stool colonies, which were pooled for PacBio sequencing. (DOC 112 kb) [file 12866_2016_891_MOESM4_ESM.doc]

>H1_16

AGTGGCAGACGGGTGAGTAACGCGTAGACAACCTGCCGTAAAGATGGGGACAACAGTTCGAAAGGACTGCTAATACCGAA

TGTTGTAGAGTTTCCGCATGGGAATCCTACTAAAGGTGGCCTCTACTTGTAAGCTATCGCTTTACGATGGGTCTGCGTCT

GATTAGCTAGTTGGTGGGGTAACGGCCTACCAAGGCGACGATCAGTAGCCGGTCTGAGAGGATGAACGGCCACATTGGAA

CTGAGACACGGTCCAGACTCCTACGGGAGGCAGCAGTGGGGAATCTTCCGCAATGGGCGCAAGCCTGACGGAGCAACGCC

GCGTGAGTGAAGAAGGGTTTCGACTCGTAAAGCTCTGTTGTCGGGGACGAATGTGGAGATGGTGAATAACCATTTTCAAT

GACGGTACCTGACGAGGAAGCCACGGCTAACTACGTGCCAGCAGCCGCGGTAATACGTAGGTGGCGAGCGTTGTCCGGAA

TTATTGGGCGTAAAGGGAGCGCAGGCGGGAAGGTAAGTCTATCTTAAAAGTGCGGGGCTCAACCCCGTGAGGGGATGGAA

ACTATCTTTCTTGAGTGCAGGAGAGGAAAGCGGAATTCCTAGTGTAGCGGTGAAATGCGTAGATATTAGGAGGAACACCA

GTGGCGAAGGCGGCTTTCTGGACTGTAACTGACGCTGAGGCTCGAAAGCGTGGGGAGCGAACAGGATTAGATACCCTGGT

AGTCCACGCCGTAAACGATGAATGCTAGGTGTAGGAGGTATCGACCCCTCCTGTGCCGGAGTTAACGCAATAAGCATTCC

GCCTGGGGAGTACGGCCGCAAGGCTGAAACTCAAAGGAATTGACGGGGGCCCGCACAAGCGGTGGAGTATGTGGTTTAAT

TCGACGCAACGCGAAGAACCTTACCAGGGCTTGACATTGAGTGAAAGGACTAGAGATAGTCCCCTCTCTTCGGAGACACG

AAAACAGGTGGTGCATGGCTGTCGTCAGCTCGTGTCGTGAGATGTTGGGTTAAGTCCCGCAACGAGCGCAACCCCTATCC

TTTGTTGCCAGCACGCAATGGTGGGAACTCAAAGGAGACTGCCGCGGACAACGCGGAGGAAGGCGGGGATGACGTCAAGT

CATCATGCCCCTTATGTCCTGGGCTACACACGTACTACAATGGGATGGACAGAGAGCAGCGAAGCCGCGAGGCCAAGCGA

ACCCCATAAACCATCTCCCAGTTCGGATTGCAGGCTGCAACCCGCCTGCATGAAGTTGGAATCGCTAGTAATCGCAGGTC

AGCATACTGCGGTGAATACGTTCCCGGGCCTTGTACACACCGCCCGTCACACCACGGAAGTCATTCACACCCGAAGCCGG

CAGGCTAACCGCAAGGAGGCAGCCGTCTAAGGTGGGGGCGATGACTGGGGTGAAGTCGT

>H1_36

AGTGGCGGACGGGTGAGTAACGCGTGGGTAACCTGCCTCATACAGGGGGATAACAGTTGGAAACGACTGCTAATACCGCA

TAAGCGCACAGGATTGCATGATCCAGTGTGAAAAACTCCGGTGGTATGAGATGGACCCGCGTCTGATTAGCCAGTTGGCG

GGGTAACGGCCCACCAAAGCGACGATCAGTAGCCGACCTGAGAGGGTGACCGGCCACATTGGGACTGAGACACGGCCCAA

ACTCCTACGGGAGGCAGCAGTGGGGAATATTGCACAATGGGGGAAACCCTGATGCAGCGACGCCGCGTGAGCGAAGAAGT

ATTTCGGTATGTAAAGCTCTATCAGCAGGGAAGAAGAATGACGGTACCTGACTAAGAAGCACCGGCTAAATACGTGCCAG

CAGCCGCGGTAATACGTATGGTGCAAGCGTTATCCGGATTTACTGGGTGTAAAGGGAGCGCAGGCGGTACGGCAAGTCTG

ATGTGAAATCCCGGGGCTCAACCCCGGTACTGCATTGGAAACTGTCGGACTAGAGTGTCGGAGGGGTAAGTGGAATTCCT

AGTGTAGCGGTGAAATGCGTAGATATTAGGAGGAACACCAGTGGCGAAGGCGGCTTACTGGACGATTACTGACGCTGAGG

CTCGAAAGCGTGGGGAGCAAACAGGATTAGATACCCTGGTAGTCCACGCCGTAAACGATGAATACTAGGTGTCGGGGAGC

ATTGCTCTTCGGTGCCGCAGCAAACGCAATAAGTATTCCACCTGGGGAGTACGTTCGCAAGAATGAAACTCAAAGGAATT

GACGGGGACCCGCACAAGCGGTGGAGCATGTGGTTTAATTCGAAGCAACGCGAAGAACCTTACCAAGTCTTGACATCCCA

CTGACAGAGTATGTAATGTACTTTCTCTTCGGAGCAGTGGTGACAGGTGGTGCATGGTTGTCGTCAGCTCGTGTCGTGAG

ATGTTGGGTTAAGTCCCGCAACGAGCGCAACCCCTATTCTTAGTAGCCAGCGGTTCGGCCGGGCACTCTAGGGAGACTGC

CAGGGATAACCTGGAGGAAGGTGGGGATGACGTCAAATCATCATGCCCCTTATGACTTGGGCTACACACGTGCTACAATG

GCGTAAACAAAGGGAAGCAATCCCGCGAGGGGGAGCAAATCTCAAAAATAACGTCTCAGTTCGGACTGTAGTCTGCAACT

CGACTACACGAAGCTGGAATCGCTAGTAATCGCGAATCAGAATGTCGCGGTGAATACGTTCCCGGGTCTTGTACACACCG

CCCGTCACACCATGGGAGTTGGTAATGCCCGAAGTCAGTGACCCAACCGCAAGGAGGGAGCTGCCGAAGGCAGGACTGAT

AACTGGGGTGAAGTCGT

>H2_9

CGGCTACCTTGTTACGACTTCACCCCCCTCACCCTCCACACCTTCGGCGCCTCCCCCCTCTCGGTTGGGCCGGCGACTTC

GGGTGCAGACGACTCGGGTGGTGTGACGGGCGGTGTGTACAAGGCCCGGGAACGCATTCACCGCGGCATGCTGATCCGCG

ATTACTAGCAACTCCGACTTCATGGGGGCGGGTTGCAGCCCCCAATCCGAACTGGGGCCGGCTTTCCGGGATCCGCTCCC

CCTCGCGGGGTGGCATCCCTCTGTACCGGCCATTGTAGCACGTGTGCAGCCCAGGGCATAAGGGGCATGATGACTTGACG

TCGTCCCCGCCCTCCTCCGCCTTGACGGCGGCGGTCCCGCGTGGGTTCCCGGCATCACCCGATGGCAACACGCGGCGGGG

GTTGCGCTCGTTGCGGGACTTAACCCAACATCTCACGACACGAGCTGACGACAGCCATGCACCACCTGTATGGGCTCCTC

TCGGCCACGGGGTCTCCCCCGCTTCACCCATATGTCAAGCCCTGGTAAGGTTCTTCGCGTTGCTTCGAATTAAGCCACAT

GCTCCGCTGCTTGTGCGGGCCCCCGTCAATTCCTTTGAGTTTTAGCCTTGCGGCCGTACTCCCCAGGCGGGACGCTTAAT

GCGTTGGCTGCGGCACGGGGGGATCGTCCCCCCACACCTAGCGTCCATCGTTTACGGCTGGGACTACCAGGGTATCTAAT

CCTGTTCGCTCCCCCAGCTTTCGCGCCTCAGCGTCGGTCTCGGCCCAGAGGGCCGCCTTCGCCACCGGTGTTCCACCCGA

TATCTGCGCATTCCACCGCTACACCGGGTGTTCCACCCTCCCCTACCGGACCCAAGCCGCGGAGGTTCCGGGGGCTTCGG

GGGGTTGAGCCCCCCGCTTCGACCCCCGGCCTGCCGGGCCGCCTACGCGCGCTTTACGCCCAATGAATCCGGATAACGCT

CGCCCCCTACGTATTACCGCGGCTGCTGGCACGTAGTTAGCCGGGGCTTCTTCTGCAGGTACAGTCTTGACTCTTCCCTG

CTGAAAGCGGTTTACGACCCGAAGGCCTCCGTCCCGCACGCGGCGTCGCTGCGTCAGGGTTCCCCCCATTGCGCAAGATT

CCCCACTGCTGCCTCCCGTAGGAGTCTGGGCCGTGTCTCAGTCCCAATCTGGCCGGTCGGTCTCTCAACCCGGCTACCCG

TTGTCGGCACGGTGGGCCGTCACCCCGCCGTCTACCTGATGGGCCGCGGAGCCATCCCCTCCCGTCGGGGCTTTAGCCCG

GGCGCCATGCGGCGCCCGGGGGTATCCGGTATTACCCGTCCTTTCGGGCGGCTATCCCGGGGGAGGGGGCAGGTTCTCCA

CGTGTTACTCAGCCGTTCGCCACTCGCTTCTATCCGAAGATAGGTGCCGTTCGAC

>H1_22

AGTGGCGGACGGGTGAGTAACGCGTGGGTAACCTGCCTCATACAGGGGAATAACAGTTGGAAACGGCTGCTAAAACCGCA

TAAGCGCACGGTATCGCATGATACAGTGTGAAAAACTCCGGTGGTATGAGATGGACCCGCGTCTGATTAGCTAGTTGGTG

AGGTAACGGCCCACCAAGGCAACGATCAGTAGCCGGCCTGAGAGGGTGAACGGCCACATTGGGACTGAGACACGGCCCAA

ACTCCTACGGGAGGCAGCAGTGGGGAATATTGCACAATGGGGGAAACCCTGATGCAGCGACGCCGCGTGAGTGATGAAGT

ATTTCGGTATGTAAAGCTCTATCAGCAGGGAAGATAATGACGGTACCTGACTAAGAAGCACCGGCTAAATACGTGCCAGC

AGCCGCGGTAATACGTATGGTGCAAGCGTTATCCGGATTTACTGGGTGTAAAGGGTGCGTAGGTGGTGAGACAAGTCTGA

AGTGAAAATCCGGGGCTTAACCCCGGAACTGCTTTGGAAACTGCCTGACTAGAGTACAGGAGAGGTAAGTGGAATTCCTA

GTGTAGCGGTGAAATGCGTAGATATTAGGAGGAACACCAGTGGCGAAGGCGACTTACTGGACTGCTACTGACACTGAGGC

ACGAAAGCGTGGGGAGCAAACAGGATTAGATACCCTGGTAGTCCACGCCGTAAACGATGAATACTAGGTGTCGGGGCCCA

AAGGGCTTCGGTGCCGCAGCCAACGCAATAAGTATTCCACCTGGGGAGTACGTTCGCAAGAATGAAACTCAAAGGAATTG

ACGGGGACCCGCACAAGCGGTGGAGCATGTGGTTTAATTCGAAGCAACGCGAAGAACCTTACCAAGTCTTGACATCCTGC

TGACCGTTCCTTAATCGGAACTTTCCTTCGGGACAGCAGAGACAGGTGGTGCATGGTTGTCGTCAGCTCGTGTCGTGAGA

TGTTGGGTTAAGTCCCGCAACGAGCGCAACCCCTATTTCCAGTAGCCAGCAGTCAGATGGGCACTCTGGAGAGACTGCCG

GGGATAACCCGGAGGAAGGTGGGGATGACGTCAAATCATCATGCCCCTTATGATTTGGGCTACACACGTGCTACAATGGC

AGTTACAAAGAGAAGCGAAACTGTGAAGTGGAGCAAACCTCAAAAAGGCTGTCTCAGTTCGGATTGTAGTCTGCAACTCG

ACTACATGAAGCTGGAATCGCTAGTAATCGCAGATCAGAATGCTGCGGTGAATACGTTCCCGGGTCTTGTACACACCGCC

CGTCACACCATGGGAGTCGGAAACGCCCGAAGTCAGTGACCCAACCGCAAGGAGGGAGCTGCCGAAGGCAGGTTCGATAA

CTGGGGTGAAGTCGT

>H2_25

CTGGGGGATAACAGTTAGAAATGACTGCTAATACCGCATAAGCGCACAGGGCCGCATGGTCTGGTGTGAAAAACTCCGGT

GGTGTAAGATGGACCCGCGTCTGATTAGGTAGTTGGTGGGGTAACGGCCCACCAAGCCGACGATCAGTAGCCGACCTGAG

AGGGTGACCGGCCACATTGGGACTGAGACACGGCCCAAACTCCTACGGGAGGCAGCAGTGGGGAATATTGGACAATGGGC

GAAAGCCTGATCCAGCGACGCCGCGTGAGTGAAGAAGTATTTCGGTATGTAAAGCTCTATCAGCAGGGAAGAAAATGACG

GTACCTGACTAAGAAGCCCCGGCTAACTACGTGCCAGCAGCCGCGGTAATACGTAGGGGGCAAGCGTTATCCGGATTTAC

TGGGTGTAAAGGGAGCGTAGACGGTTAAGCAAGTCTGAAGTGAAAGCCCGGGGCTCAACCCCGGTACTGCTTTGGAAACT

GTTTGACTTGAGTGCAGGAGAGGTAAGTGGAATTCCTAGTGTAGCGGTGAAATGCGTAGATATTAGGAGGAACACCAGTG

GCGAAGGCGGCTTACTGGACTGTAACTGACGTTGAGGCTCGAAAGCGTGGGGAGCAAACAGGATTAGATACCCTGGTAGT

CCACGCCGTAAACGATGAATACTAGGTGTCGGGGGACAAAGTCCTTCGGTGCCGCCGCTAACGCAATAAGTATTCCACCT

GGGGAGTACGTTCGCAAGAATGAAACTCAAAGGAATTGACGGGGACCCGCACAAGCGGTGGAGCATGTGGTTTAATTCGA

AGCAACGCGAAGAACCTTACCAAGTCTTGACATCCCATTGAAAACCCTTTAACCGGGGTCCCTCTTCGGAGCAATGGAGA

CAGGTGGTGCATGGTTGTCGTCAGCTCGTGTCGTGAGATGTTGGGTTAAGTCCCGCAACGAGCGCAACCCTTATCCTTAG

TAGCCAGCAAGTAATGTTGGGCACTCTGGGGAGACTGCCAGGGATAACCTGGAGGAAGGTGGGGATGACGTCAAATCATC

ATGCCCCTTATGATTTGGGCTACACACGTGCTACAATGGCGTAAACAAAGGGAAGCAAAGGAGCGATCTGGAGCAAACCC

CAAAAATAACGTCTCAGTTCGGATTGCAGGCTGCAACTCGCCTGCATGAAGCTGGAATCGCTAGTAATCGCGAATCAGAA

TGTCGCGGTGAATACGTTCCCGGGTCTTGTACACACCGCCCGTCACACCATGGGAGTTGGTAACGCCCGAAGTCAGTGAC

CCAACCGTAAGGAGGGAGCTGCCGAAGGCGGGACTGATAACTGGGGTGAAGTCGTAACAAGGTAACCG

>H2_31

TTACGACTTCACCCCAATCATCAACCCCACCTTCGACTGCTGCCTCCTAATAGGTTAGCTCACAGGCTTCGGGTGTTATC

GACTCTCATGGTGTGACGGGCGGTGTGTACAAGGCCCGGGAACGTATTCACCGCGGCATTCTGATCCGCGATTACTAGCA

ATTCCAACTTCATGCAGGCGAGTTGCAGCCTGCAATCTGAACTGAGACCGAGTTTTGTGATTCGCTCCACCTCACGGTTT

CGCTGCACTTTGTTATCGGCCATTGTAGTACGTGTGTAGCCCTGGGCATAAGGGGCATGATGATTTGACGTCGTCCCCAC

CTTCCTCCGATTTGTCACCGGCAGTCTCGCCAGAGTGCCCAACTTAATGATGGCAACTGACAATAGGGGTTGCGCTCGTT

GCGGGACTTAACCCAACATCTCACGACACGAGCTGACGACAACCATGCACCACCTGTCACCTGATTCCCGAAGGCACTCA

AGTATCTCTACTCGATTCCAGGGATGTCAAGCCCAGGTAAGGTTCTTCGCGTTGCTTCGAATTAAACCACATACTCCACT

GCTTGTGCGGGCCCCCGTCAATTCCTTTGAGTTTCAACCTTGCGGTCGTACTCCCCAGGTGGGATACTTATTGTGTTAAC

TGCGGCACAGAAGGGGTCGATACCTCCTACACCTAGTATCCATCGTTTACGGTGTGGACTACCAGGGTATCTAATCCTGT

TTGCTCCCCACACTTTCGCGCCTCAGCGTCAGTTGTCGTCCAGAAAGCCGCCTTCGCCACTGGTGTTCCTCCTAATATCT

ACGCATTTCACCGCTACACTAGGAATTCCGCTTTCCTCTCCGATACTCAAGGCTGTCAGTTTCAATCGCAGCCCCGGGGT

TAAGCCCCGGTATTTCACGACTGACTTGACAGTCCGCCTACGCGCCCTTTACACCCAGTAAATCCGGACAACGCTTGTCA

CCTACGTATTACCGCGGCTGCTGGCACGTAGTTAGCCGTGACTTTCTCACAAGGTACCGTCACTGTCTTCTTCCCTTGCG

ACAACAGTTTACAATCCGAAGACCTTCTTCCTGCACGCGGCGTTGCTGCGTCAGGGTTGCCCCCATTGCGCAATATTCCC

CACTGCTGCCTCCCGTAGGAGTCTGGGCCGTGTCTCAGTCCCAATGCGGCCGATCAACCTCTCAGTTCGGCTACAGATCG

TTGACTTGGTGAGCCGTTACCTCACCAACTATCTAATCTGACGCGAGTCCATCCATAAGCGATAAATCTTTCATAATCAA

GTCATGCAACTTAACTACAATATGCGGTATTAGTCCACGTTTCCATGGATTATCCCCCTCTTATGGGCAGGTTACTCACG

CGTTACTCGCCCGTCCGCCACTTTCCTCTTTGAACGTCACTCCGAAAAGATCTGTTCAAAGATTCTCGTCGACT

>H3_2

AGTCGAGCGAGTGATCTCCTTCGGGAGTGAAGCTAGCGGCGGACGGGTGAGTAACACGTGGGCAACCTGCCTCATAGAGG

GGAATAGCCTTCCGAAAGGAAGATTAATACCGCATAAGATTGTAGCTTCGCATGAAGTAGCAATTAAAGGAGCAATCCGC

TATGAGATGGGCCCGCGGCGCATTAGCTAGTTGGTGAGGTAACGGCTCACCAAGGCGACGATGCGTAGCCGACCTGAGAG

GGTGATCGGCCACATTGGGACTGAGACACGGCCCAGACTCCTACGGGAGGCAGCAGTGGGGAATATTGCACAATGGGGGA

AACCCTGATGCAGCAACGCCGCGTGAGTGATGACGGCCTTCGGGTTGTAAAGCTCTGTCTTCAGGGACGATAATGACGGT

ACCTGAGGAGGAAGCCACGGCTAACTACGTGCCAGCAGCCGCGGTAATACGTAGGTGGCGAGCGTTGTCCGGATTTACTG

GGCGTAAAGGGAGCGTAGGCGGACTTTTAAGTGAGATGTGAAATACCCGGGCTCAACTTGGGTGCTGCATTTCAAACTGG

AAGTCTAGAGTGCAGGAGAGGAGAATGGAATTCCTAGTGTAGCGGTGAAATGCGTAGAGATTAGGAAGAACACCAGTGGC

GAAGGCGATTCTCTGGACTGTAACTGACGCTGAGGCTCGAAAGCGTGGGGAGCAAACAGGATTAGATACCCTGGTAGTCC

ACGCCGTAAACGATGAATACTAGGTGTAGGGGTTGTCATGACCTCTGTGCCGCCGCTAACGCATTAAGTATTCCGCCTGG

GGAGTACGGTCGCAAGATTAAAACTCAAAGGAATTGACGGGGGCCCGCACAAGCAGCGGAGCATGTGGTTTAATTCGAAG

CAACGCGAAGAACCTTACCTAGACTTGACATCTCCTGCATTACCCTTAATCGGGGAAGTTCCTTCGGGAACAGGAAGACA

GGTGGTGCATGGTTGTCGTCAGCTCGTGTCGTGAGATGTTGGGTTAAGTCCCGCAACGAGCGCAACCCTTATTGTTAGTT

GCTACCATTTAGTTGAGCACTCTAGCGAGACTGCCCGGGTTAACCGGGAGGAAGGTGGGGATGACGTCAAATCATCATGC

CCCTTATGTCTAGGGCTACACACGTGCTACAATGGCAAGTACAAAGAGAAGCAAGACCGCGAGGTGGAGCAAAACTCAAA

AACTTGTCTCAGTTCGGATTGTAGGCTGAAACTCGCCTACATGAAGCTGGAGTTGCTAGTAATCGCGAATCAGCATGTCG

CGGTGAATACGTTCCCGGGCCTTGTACACACCGCCCGTCACACCATGAGAGTTGGCAATACCCAAAGTGCGTGATCTGAC

TCGCAAGAGAGGAAGCGCCCTAAGGTAGGGTCAGCGATTGGGGTGAAGTCGTAACAAGGTAACCGTAGAT

>H3_7

AGTCGACGGGATCCAGGAGCTTGCTCCTGGGTGAGAGTGGCGAACGGGTGAGTAATGCGTGACCGACCTGCCCCATACAC

CGGAATAGCTCCTGGAAACGGGTGGTAATGCCGGATGCTCCAGTTGACCGCATGGTCCTCTGGGAAAGATTTTTCGGTAT

GGGATGGGGTCGCGTCCTATCAGCTTGATGGCGGGGTAACGGCCCACCATGGCTTCGACGGGTAGCCGGCCTGAGAGGGC

GACCGGCCACATTGTGACTGAGATACGGCCCACACTCCTACGGGAGGCAGCAGTGGGGAATATTGCACAATGGGCGCAAG

CCTGATGCAGCGACGCCGCGTGCGGGATGACGGCCTTCGGGTTGTAAACCGCTTTTGACTGGGAGCAAGCCCTTCGGGGT

GAGTGTACCTTTCTAATAAGCACCGGCTAACTACGTGCCAGCAGCCGCGGTAATACGTATGGTGCAAGCGTTATCCGGAA

TTATTGGGCGTAAAGGGCTCGTATGCGGTTCGTCGCGTCCGGTGTGAAAGTCCATCGCTTAACGGTGGATCCGCGCCGGG

TACAGGCGGGCTTGAGTGCGGTAGGGGAGACTGTAATTCTCGGTGTAACGGTGGAATGTGTAGATATCGGGAAGAACACC

AATGGCGAAGGCAGGTCTCTGGGCCGTCACTGACGCTGAGGAGCGAAAGCGTGGGGAGCGAACAGGATTAGATACCCTGG

TAGTCCACGCCGTAAACGGTGGATGCTGGATGTGGGGACCATTCCACGGTCTCCGTGTCGGAGCCAACGCGTTAAGCATC

CCGCCTGGGGAGTACGGCCGCAAGGCTAAAACTCAAAGAAATTGACGGGGGCCCGCACAAGCGGCGGAGCATGCGGATTA

ATTCGATGCAACGCGAAGAACCTTACCTGGGCTTGACATGTTCCCGACAGCCCCAGAGATGGGGCCTCCCTTCGGGGCGG

GTTCACAGGTGGTGCATGGTCGTCGTCAGCTCGTGTCGTGAGATGTTGGGTTAAGTCCCGCAACGAGCGCAACCCTCGCC

CTGTGTTGCCAGCACGTCGTGGTGGGAACTCACGGGGGACCGCCGGGGTCAACTCGGAGGAAGGTGGGGATGACGTCAGA

TCATCATGCCCCTTACGTCCAGGGCTTCACGCATGCTACAATGGCCGGTACAACGGGATGCGACACCGCGAGGTGGAGCG

GATCCCTTAAAACCGGTCTCAGTTCGGATTGGAGTCTGCAACCCGACTCCATGAAGGCGGAGTCGCTAGTAATCGCGGAT

CAGCAACGCCGCGGTGAATGCGTTCCCGGGCCTTGTACACACCGCCCGTCAAGTCATGAAAGTGGGTAGCACCCGAAGCC

GGTGGCCCAACCTTGGGG

>H3_8

GTCGAACGAAGCACTCTATTTGATTTTCTTCGGAAATGAAGATTTTGTGACTGAGTGGCGGACGGGTGAGTAACGCGTGG

GTAACCTGCCTCATACAGGGGGATAACAGTTGGAAACGACTGCTAATACCGCATAAGCGCACAGGATCGCATGATCCGGT

GTGAAAAACTCCGGTGGTATGAGATGGACCCGCGTCTGATTAGCCAGTTGGCAGGGTAACGGCCTACCAAAGCGACGATC

AGTAGCCGACCTGAGAGGGTGACCGGCCACATTGGGACTGAGACACGGCCCAAACTCCTACGGGAGGCAGCAGTGGGGAA

TATTGCACAATGGGGGAAACCCTGATGCAGCGACGCCGCGTGAGCGAAGAAGTATTTCGGTATGTAAAGCTCTATCAGCA

GGGAAGAAGAATGACGGTACCTGACTAAGAAGCACCGGCTAAATACGTGCCAGCAGCCGCGGTAATACGTATGGTGCAAG

CGTTATCCGGATTTACTGGGTGTAAAGGGAGCGCAGGCGGTGCGGCAAGTCTGATGTGAAAGCCCGGGGCTCAACCCCGG

TACTGCATTGGAAACTGTCGTACTAGAGTGTCGGAGGGGTAAGTGGAATTCCTAGTGTAGCGGTGAAATGCGTAGATATT

AGGAGGAACACCAGTGGCGAAGGCGGCTTACTGGACGATAACTGACGCTGAGGCTCGAAAGCGTGGGGAGCAAACAGGAT

TAGATACCCTGGTAGTCCACGCCGTAAACGATGAATACTAGGTGTCGGGGAGCATTGCTCTTCGGTGCCGCAGCAAACGC

AATAAGTATTCCACCTGGGGAGTACGTTCGCAAGAATGAAACTCAAAGGAATTGACGGGGACCCGCACAAGCGGTGGAGC

ATGTGGTTTAATTCGAAGCAACGCGAAGAACCTTACCAAGTCTTGACATCCCGATGACAGAGTATGTAATGTACTTTCTC

TTCGGAGCATCGGTGACAGGTGGTGCATGGTTGTCGTCAGCTCGTGTCGTGAGATGTTGGGTTAAGTCCCGCAACGAGCG

CAACCCCTGTTCTTAGTAGCCAGCGGTTCGGCCGGGCACTCTAGGGAGACTGCCAGGGATAACCTGGAGGAAGGCGGGGA

TGACGTCAAATCATCATGCCCCTTATGACTTGGGCTACACACGTGCTACAATGGCGTAAACAAAGGGAAGCGGAGCCGTG

AGGCCGAGCAAATCTCAAAAATAACGTCTCAGTTCGGACTGTAGTCTGCAACCCGACTACACGAAGCTGGAATCGCTAGT

AATCGCAGATCAGAATGCTGCGGTGAATACGTTCCCGGGTCTTGTACACACCGCCCGTCACACCATGGGAGTTGGAAATG

CCCGAAGTCAGTGACCCAACCGCAAGGAGGGAGCTGCCGAAGGCAGGTTCGATAACTGGGGTGAAGTCGTA

>H3_28

AGTCGACGCTTCCATATGTGAAGAGTGGCGAACGGGTGAGTAATACATAAGTAACCTGGCCTTTACAGGGGGATAACTAT

TGGAAACGATAGCTAAGACCGCATAGGTGTCATAACCGCATGGAGATGATATGAAATATGCTACGGCATAGGTAGAGGAT

GGACTTATGGCGCATTAGCTAGTTGGAGGGGTAACGGCCCACCAAGGCGACGATGCGTAGCCGACCTGAGAGGGTGACCG

GCCACACTGGGACTGAGACACGGCCCAGACTCCTACGGGAGGCAGCAGTAGGGAATTTTCGGCAATGGGGGAAACCCTGA

CCGAGCAACGCCGCGTGAAGGAAGAAGTAATTCGTTATGTAAACTTCTGTCATAGAGGAAGAACGGTGCGTGTAGGGAAT

GACATGCAAGTGACGGTACTCTATAAGAAAGCCACGGCTAACTACGTGCCAGCAGCCGCGGTAATACGTAGGTGGCGAGC

GTTATCCGGAATTATTGGGCGTAAAGAGGGAGCAGGCGGCACTAAGGGTCTGTGGTGAAAGATCGAAGCTTAACTTCGGT

AAGCCATGGAAACCGTAGAGCTAGAGTGTGTGAGAGGATCGTGGAATTCCATGTGTAGCGGTGAAATGCGTAGATATATG

GAGGAACACCAGTGGCGAAGGCGACGATCTGGCGCATAACTGACGCTCAGTCCCGAAAGCGTGGGGAGCAAATAGGATTA

GATACCCTAGTAGTCCACGCCGTAAACGATGAGTACTAAGTGTTGGGAGTCAAATCTCAGTGCTGCAGTTAACGCAATAA

GTACTCCGCCTGAGTAGTACGTTCGCAAGAATGAAACTCAAAGGAATTGACGGGGGCCCGCACAAGCGGTGGAGCATGTG

GTTTAATTCGAAGCAACGCGAAGAACCTTACCAGGTCTTGACATCGATCTAAAGGCTCCAGAGATGGAGAGATAGCTATA

GAGAAGACAGGTGGTGCATGGTTGTCGTCAGCTCGTGTCGTGAGATGTTGGGTTAAGTCCCGCAACGAGCGCAACCCCTG

TTGCCAGTTGCCAGCATTAAGTTGGGGACTCTGGCGAGACTGCCGGTGACAAGCCGGAGGAAGGCGGGGATGACGTCAAA

TCATCATGCCCCTTATGACCTGGGCTACACACGTGCTACAATGGACAGAGCAGAGGGAAGCGAAGCCGCGAGGTGGAGCG

AAACCCAGAAAACTGTTCTCAGTTCGGACTGCAGTCTGCAACTCGACTGCACGAAGTTGGAATCGCTAGTAATCGCGAAT

CAGCATGTCGCGGTGAATACGTTCTCGGGCCTTGTACACACCGCCCGTCACACCATGAGAGTCGGTAACACCCGAAGCCG

GTGGC

>H3_29

AGTCGACGCTTCCTTCGGTGAAGAGTGGCGAACNGGGTGAGTAATACATAAGTAACCTGGCATCTACAGGGGGATAACTG

ATGGAAACGTCAGCTAAGACCGCATAGGTGTAGAGATCGCATGAACTCTATATGAAAAGTGCTACGGGACTGGTAGATGA

TGGACTTATGGCGCATTAGCTTGTTGGTAGGGTAACGGCCTACCAAGGCGACGATGCGTAGCCGACCTGAGAGGGTGACC

GGCCACACTGGGACTGAGACACGGCCCAGACTCCTACGGGAGGCAGCAGTAGGGAATTTTCGGCAATGGGGGAAACCCTG

ACCGAGCAACGCCGCGTGAAGGAAGAAGTAATTCGTTATGTAAACTTCTGTCATAGAGGAAGAACGGTGGATATAGGGAA

TGATATCCAAGTGACGGTACTCTATAAGAAAGCCACGGCTAACTACGTGCCAGCAGCCGCGGTAATACGTAGGTGGCGAG

CGTTATCCGGAATTATTGGGCGTAAAGAGGGAGCAGGCGGCACTAAGGGTCTGTGGTGAAAGATCGAAGCTTAACTTCGG

TAAGCCATGGAAACCGTAGAGCTAGAGTGTGTGAGAGGATCGTGGAATTCCATGTGTAGCGGTGAAATGCGTAGATATAT

GGAGGAACACCAGTGGCGAAGGCGACGATCTGGCGCATAACTGACGCTCAGTCCCGAAAGCGTGGGGAGCAAATAGGATT

AGATACCCTAGTAGTCCACGCCGTAAACGATGAGTACTAAGTGTTGGGTGTCAAAGCTCAGTGCTGCAGTTAACGCAATA

AGTACTCCGCCTGAGTAGTACGTTCGCAAGAATGAAACTCAAAGGAATTGACGGGGGCCCGCACAAGCGGTGGAGCATGT

GGTTTAATTCGAAGCAACGCGAAGAACCTTACCAGGTCTTGACATCGATCTAAAGGCTCCAGAGATGGAGAGATAGCTAT

AGAGAAGACAGGTGGTGCATGGTTGTCGTCAGCTCGTGTCGTGAGATGTTGGGTTAAGTCCCGCAACGAGCGCAACCCCT

GTTGCCAGTTGCCAGCATTAAGTTGGGGACTCTGGCGAGACTGCCGGTGACAAGCCGGAGGAAGGCGGGGATGACGTCAA

ATCATCATGCCCCTTATGACCTGGGCTACACACGTGCTACAATGGACAGAGCAGAGGGAAGCGAAGCCGCGAGGTGGAGC

GAAACCCATAAAACTGTTCTCAGTTCGGACTGCAGTCTGCAACTCGACTGCACGAAGATGGAATCGCTAGTAATCGCGAA

TCAGCATGTCGCGGTGAATACGTTCTCGGGCCTTGTACACACCGCCCGTCACACCATGAGAGTCGGTAACACCCGAAGCC

GGTGGCCTAACCGCAAGGAAGGAGCTGTCTAAGGTGGGACTGATGATTGGGGTGAAGTCGTAACAGGGTAACCG

>H3_38

AGTCGACGGGATCCATCGGGCTTTGCTTGGTGGTGAGAGTGGCGAACGGGTGAGTAATGCGTGACCGACCTGCCCCATGC

TCCGGAATAGCTCCTGGAAACGGGTGGTAATGCCGGATGTTCCACATGATCGCATGTGATTGTGGGAAAGATTCTATCGG

CGTGGGATGGGGTCGCGTCCTATCAGCTTGTTGGTGAGGTAACGGCTCACCAAGGCTTCGACGGGTAGCCGGCCTGAGAG

GGCGACCGGCCACATTGGGACTGAGATACGGCCCAGACTCCTACGGGAGGCAGCAGTGGGGAATATTGCACAATGGGCGC

AAGCCTGATGCAGCGACGCCGCGTGAGGGATGGAGGCCTTCGGGTTGTAAACCTCTTTTGTTTGGGAGCAAGCCTTCGGG

TGAGTGTACCTTTCGAATAAGCGCCGGCTAACTACGTGCCAGCAGCCGCGGTAATACGTAGGGCGCAAGCGTTATCCGGA

TTTATTGGGCGTAAAGGGCTCGTAGGCGGCTCGTCGCGTCCGGTGTGAAAGTCCATCGCTTAACGGTGGATCTGCGCCGG

GTACGGGCGGGCTGGAGTGCGGTAGGGGAGACTGGAATTCCCGGTGTAACGGTGGAATGTGTAGATATCGGGAAGAACAC

CGATGGCGAAGGCAGGTCTCTGGGCCGTCACTGACGCTGAGGAGCGAAAGCGTGGGGAGCGAACAGGATTAGATACCCTG

GTAGTCCACGCCGTAAACGGTGGACGCTGGATGTGGGGCACGTTCCACGTGTTCCGTGTCGGAGCTAACGCGTTAAGCGT

CCCGCCTGGGGAGTACGGCCGCAAGGCTAAAACTCAAAGAAATTGACGGGGGCCCGCACAAGCGGCGGAGCATGCGGATT

AATTCGATGCAACGCGAAGAACCTTACCTGGGCTTGACATGTTCCCGACGACGCCAGAGATGGCGTTTCCCTTCGGGGCG

GGTTCACAGGTGGTGCATGGTCGTCGTCAGCTCGTGTCGTGAGATGTTGGGTTAAGTCCCGCAACGAGCGCAACCCTCGC

CCCGTGTTGCCAGCACGTTATGGTGGGAACTCACGGGGGACCGCCGGGGTTAACTCGGAGGAAGGTGGGGATGACGTCAG

ATCATCATGCCCCTTACGTCCAGGGCTTCACGCATGCTACAATGGCCGGTACAGCGGGATGCGACATGGCGACATGGAGC

GGATCCCTGAAAACCGGTCTCAGTTCGGATCGGAGCCTGCAACCCGGCTCCGTGAAGGCGGAGTCGCTAGTAATCGCGGA

TCAGCAACGCCGCGGTGAATGCGTTCCCGGGCCTTGTACACACCGCCCGTCAAGTCATGAAAGTGGGCAGCACCCGAAGC

CGGTGGCCTAACCCCTTGTGGGATGAG

>H3_48

AACGCGTGGGTAACCTGCCTTGTACTGGGGGACAACAGTTGGAAACGACTGCTAATACCGCATAAGCGCACAGCTTCGCA

TGAAGCAGTGTGAAAAACTCCGGTGGTACAAGATGGACCCGCGTCTGATTAGCTGGTTGGTGAGGTAACGGCCCACCAAG

GCGACGATCAGTAGCCGGCCTGAGAGGGTGAACGGCCACATTGGGACTGAGACACGGCCCAAACTCCTACGGGAGGCAGC

AGTGGGGAATATTGCACAATGGGGGAAACCCTGATGCAGCAACGCCGCGTGAGTGAAGAAGTATTTCGGTATGTAAAGCT

CTATCAGCAGGAAAGAAAATGACGGTACCTGACTAAGAAGCCCCGGCTAACTACGTGCCAGCAGCCGCGGTAATACGTAG

GGGGCAAGCGTTATCCGGATTTACTGGGTGTAAAGGGAGCGTAGACGGTTTTGCAAGTCTGAAGTGAAAGCCCGGGGCTT

AACCCCGGGACTGCTTTGGAAACTGTAGGACTAGAGTGCAGGAGAGGTAAGTGGAATTCCTAGTGTAGCGGTGAAATGCG

TAGATATTAGGAGGAACACCAGTGGCGAAGGCGGCTTACTGGACTGTAACTGACGTTGAGGCTCGAAAGCGTGGGGAGCA

AACAGGATTAGATACCCTGGTAGTCCACGCCGTAAACGATGATTACTAGGTGTTGGTGGGTACGACCCATCGGTGCCGCA

GCAAACGCAATAAGTAATCCACCTGGGGAGTACGTTCGCAAGAATGAAACTCAAAGGAATTGACGGGGACCCGCACAAGC

GGTGGAGCATGTGGTTTAATTCGAAGCAACGCGAAGAACCTTACCTGGTCTTGACATCCCTATGAATAACGGGCAATGCC

GTTAGTACTTCGGTACATAGGAGACAGGTGGTGCATGGTTGTCGTCAGCTCGTGTCGTGAGATGTTGGGTTAAGTCCCGC

AACGAGCGCAACCCTTATCTTTAGTAGCCAGCAGTAAGATGGGCACTCTAGAGAGACTGCCGGGGATAACCCGGAGGAAG

GTGGGGATGACGTCAAATCATCATGCCCCTTATGACCAGGGCTACACACGTGCTACAATGGCGTAAACAAAGAGAAGCGA

AGTCGTGAGGCAGAGCGAATCTCAAAAATAACGTCTCAGTTCGGATTGTAGTCTGCAACTCGACTACATGAAGCTGGAAT

CGCTAGTAATCGCAGATCAGAATGCTGCGGTGAATACGTTCCCGGGTCTTGTACACACCGCCCGTCACACCATGGGAGTC

GGAAATGCCCGAAGTCGGTGACCTAACCGAAAGGAAGGAGCCGCCGAAGGCAGGTCTGATAACTGGGGTGAAGTCG

>H3_49

AGTCGACGGAGATGCGATGTGAGCGAGAGGTGCTTGCACTGATCAATCTTTTCGTATCTTAGTGGCGGACGGGTGAGTAA

CGCGTGGGTAACCTGCCTTATACCGGGGGATAACACTTAGAAATAGGTGCTAATACCGCATAAGCGCACGGTGTCGCATG

ACACAGTGTGAAAAACTCCGGTGGTATAAGATGGACCCGCGTCTGATTAGCCAGTTGGCAGGGTAACGGCCTACCAAAGC

GACGATCAGTAGCCGGCCTGAGAGGGTGAACGGCCACATTGGGACTGAGACACGGCCCAAACTCCTACGGGAGGCAGCAG

TGGGGAATATTGCACAATGGGGGAAACCCTGATGCAGCGACGCCGCGTGAGTGAAGAAGTATTTCGGTATGTAAAGCTCT

ATCAGCAGGGAAGAAGAAATGACGGTACCTGACTAAGAAGCCCCGGCTAACTACGTGCCAGCAGCCGCGGTAATACGTAG

GGGGCAAGCGTTATCCGGATTTACTGGGTGTAAAGGGAGCGTAGACGGTGAAGCAAGTCTGAAGTGAAAGGTTGGGGCTC

AACCCCGAAACTGCTTTGGAAACTGTTTAACTGGAGTACAGGAGAGGTAAGTGGAATTCCTAGTGTAGCGGTGAAATGCG

TAGATATTAGGAGGAACACCAGTGGCGAAGGCGGCTTACTGGACTGTAACTGACGTTGAGGCTCGAAAGCGTGGGGAGCA

AACAGGATTAGATACCCTGGTAGTCCACGCCGTAAACGATGATTACTAGGTGTTGGTGGATATGGATCCATCGGTGCCGC

AGCAAACGCAATAAGTAATCCACCTGGGGAGTACGTTCGCAAGAATGAAACTCAAAGGAATTGACGGGGACCCGCACAAG

CGGTGGAGCATGTGGTTTAATTCGAAGCAACGCGAAGAACCTTACCTGATCTTGACATCCCTATGAATACAGGGTAATGC

CTGTAGTACTTCGGTACATAGGAGACAGGTGGTGCATGGTTGTCGTCAGCTCGTGTCGTGAGATGTTGGGTTAAGTCCCG

CAACGAGCGCAACCCCTATCTTTAGTAGCCAGCAGTAAGATGGGCACTCTAGAGAGACTGCCGGGGATAACCCGGAGGAA

GGTGGGGATGACGTCAAATCATCATGCCCCTTATGACCAGGGCTACACACGTGCTACAATGGCGTAAACAGAGGGAAGCG

AAGTGGTGACATGGAGCAAATCCCAAAAATAACGTCCCAGTTCGGATTGCAGGCTGCAACTCGCCTGCATGAAGCTGGAA

TCGCTAGTAATCGCAGATCAGAATGCTGCGGTGAATACGTTCCCGGGTCTTGTACACACCGCCCGTCACACCATGGGAGT

AGGTAATGCCCGAAGTCGGTGACCTAACCGCAAGGAAGGAGCCGCCGAAGGCAGGACTTATAACTGGGGTGAAGTCGTAA

CAAGGTAACCGT

>H3_19

CCGGTGGTTATAGATGGACATGCGTTGTATTAGCTAGTTGGTGAGGTAACGGCTCACCAAGGCAACGATACATAGGGGGA

CTGAGAGGTTAACCCCCCACACTGGTACTGAGACACGGACCAGACTCCTACGGGAGGCAGCAGTGAGGAATATTGGTCAA

TGGACGCAAGTCTGAACCAGCCATGCCGCGTGCAGGAAGACGGCTCTATGAGTTGTAAACTGCTTTTGTACGAGGGTAAA

CTCACCTACGTGTAGGTGACTGAAAGTATCGTACGAATAAGGATCGGCTAACTCCGTGCCAGCAGCCGCGGTAATACGGA

GGATTCAAGCGTTATCCGGATTTATTGGGTTTAAAGGGTGCGTAGGCGGTTTGATAAGTTAGAGGTGAAATCCCGGGGCT

TAACTCCGGAACTGCCTCTAATACTGTTAGACTAGAGAGTAGTTGCGGTAGGCGGAATGTATGGTGTAGCGGTGAAATGC

TTAGAGATCATACAGAACACCGATTGCGAAGGCAGCTTACCAAACTATATCTGACGTTGAGGCACGAAAGCGTGGGGAGC

AAACAGGATTAGATACCCTGGTAGTCCACGCAGTAAACGATGATAACTCGTTGTCGGCGATACACAGTCGGTGACTAAGC

GAAAGCGATAAGTTATCCACCTGGGGAGTACGTTCGCAAGAATGAAACTCAAAGGAATTGACGGGGGCCCGCACAAGCGG

AGGAACATGTGGTTTAATTCGATGATACGCGAGGAACCTTACCCGGGCTTGAAAGTTACTGACGATTCTGGAAACAGGAT

TTCCCTTTGGGGCAGGAAACTAGGTGCTGCATGGTTGTCGTCAGCTCGTGCCGTGAGGTGTCGGGTTAAGTCCCATAACG

AGCGCAACCCCTACCGTTAGTTGCCATCAGGTCAAGCTGGGCACTCTGGCGGGACTGCCGGTGTAAGCCGAGAGGAAGGT

GGGGATGACGTCAAATCAGCACGGCCCTTACGTCCGGGGCTACACACGTGTTACAATGGTAGGTACAGAGGGTCGCTACT

CCGTGAGGAGATGCCAATCTCGAAAGCCTATCTCAGTTCGGATTGGAGGCTGAAACCCGCCTCCATGAAGTTGGATTCGC

TAGTAATCGCGCATCAGCCATGGCGCGGTGAATACGTTCCCGGGCCTTGTACACACCGCCCGTCAAGCCATGGAAGCTGG

GGGTGCCTGAAGTTCGTGACCGCAAGGAGCGACCTAGGGCAAAACCGGTAACTGGGGCTAAGTCGTAAC

>H3_40

AGTCGACGGGATCCATCAGGCTTTGCTTGGTGGTGAGAGTGGCGAACGGGTGAGTAATGCGTGACCGACCTGCCCCATAC

ACCGGAATAGCTCCTGGAAACGGGTGGTAATGCCGGATGCTCCGACTCCTCGCATGGGGTGTCGGGAAAGATTTCATCGG

TATGGGATGGGGTCGCGTCCTATCAGGTAGTCGGCGGGGTAACGGCCCACCGAGCCTACGACGGGTAGCCGGCCTGAGAG

GGCGACCGGCCACATTGGGACTGAGATACGGCCCAGACTCCTACGGGAGGCAGCAGTGGGGAATATTGCACAATGGGCGC

AAGCCTGATGCAGCGACGCCGCGTGCGGGATGACGGCCTTCGGGTTGTAAACCGCTTTTGATCGGGAGCAAGCCTTCGGG

TGAGTGTACCTTTCGAATAAGCACCGGCTAACTACGTGCCAGCAGCCGCGGTAATACGTAGGGTGCAAGCGTTATCCGGA

ATTATTGGGCGTAAAGGGCTCGTAGGCGGTTCGTCGCGTCCGGTGTGAAAGTCCATCGCTTAACGGTGGATCTGCGCCGG

GTACGGGCGGGCTGGAGTGCGGTAGGGGAGACTGGAATTCCCGGTGTAACGGTGGAATGTGTAGATATCGGGAAGAACAC

CAATGGCGAAGGCAGGTCTCTGGGCCGTTACTGACGCTGAGGAGCGAAAGCGTGGGGAGCGAACAGGATTAGATACCCTG

GTAGTCCACGCCGTAAACGGTGGATGCTGGATGTGGGGCCCGTTCCACGGGTTCCGTGTCGGAGCTAACGCGTTAAGCAT

CCCGCCTGGGGAGTACGGCCGCAAGGCTAAAACTCAAAGAAATTGACGGGGGCCCGCACAAGCGGCGGAGCATGCGGATT

AATTCGATGCAACGCGAAGAACCTTACCTGGGCTTGACATGTTCCCGACAGCCGTAGAGATATGGCCTCCCTTCGGGGCG

GGTTCACAGGTGGTGCATGGTCGTCGTCAGCTCGTGTCGTGAGATGTTGGGTTAAGTCCCGCAACGAGCGCAACCCTCGC

CCTGTGTTGCCAGCACGTCATGGTGGGAACTCACGGGGGACCGCCGGGGTCAACTCGGAGGAAGGTGGGGATGACGTCAG

ATCATCATGCCCCTTACGTCCAGGGCTTCACGCATGCTACAATGGCCGGTACAACGGGATGCGACACGGCGACGTGGAGC

GGATCCCTGAAAACCGGTCTCAGTTCGGATTGGAGTCTGCAACCCGACTCCATGAAGGCGGAGTCGCTAGTAATCGCGGA

TCAGCAACGCCGCGGTGAATGCGTTCCCGGGCCTTGTACACACCGCCCGTCAAGTCATGAAAGTGGGTAGCACCCGAAGC

CGGTGGCCTACCCTTGGGATGAG

>H4_22

TCTTCGGAAGTGAGCGGCGGACGGGTGAGTAACGCGTGGGTAACCTGCCCTATACACACGGATAACGTACCGAAAGGTAC

GCTAATACGAGATGATATATTTTTATCGCATGGTAGAAATATCAAAGCTCCGGCGGTATAGGATGGACCCGCGTCTGATT

AGCTAGTTGGTAAGGTAACGGCTTACCAAGGCGACGATCAGTAGCCGACCTGAGAGGGTGATCGGCCACATTGGAACTGA

GACACGGTCCAAACTCCTACGGGAGGCAGCAGTGGGGAATATTGCACAATGGGCGAAAGCCTGATGCAGCAACGCCGCGT

GAGCGATGAAGGCCTTCGGGTCGTAAAGCTCTGTCCTCAAGGAAGATAATGACGGTACTTGAGGAGGAAGCCCCGGCTAA

CTACGTGCCAGCAGCCGCGGTAATACGTAGGGGGCTAGCGTTATCCGGAATTACTGGGCGTAAAGGGTGCGTAGGTGGTT

TCTTAAGTCAGAGGTGAAAGGCTACGGCTCAACCGTAGTAAGCCTTTGAAACTGGGAAACTTGAGTGCAGGAGAGGAGAG

TAGAATTCCTAGTGTAGCGGTGAAATGCGTAGATATTAGGAGGAATACCAGTTGCGAAGGCGGCTCTCTGGACTGTAACT

GACACTGAGGCACGAAAGCGTGGGGAGCAAACAGGATTAGATACCCTGGTAGTCCACGCCGTAAACGATGAGTACTAGCT

GTCGGAGGTTACCCCCTTCGGTGGCGCAGCTAACGCATTAAGTACTCCGCCTGGGAAGTACGCTCGCAAGAGTGAAACTC

AAAGGAATTGACGGGGACCCGCACAAGTAGCGGAGCATGTGGTTTAATTCGAAGCAACGCGAAGAACCTTACCTAAGCTT

GACATCCTTTTGACCTCTCCCTAATCGGAGATTTCCCTTCGGGGACAGAAGTGACAGGTGGTGCATGGTTGTCGTCAGCT

CGTGTCGTGAGATGTTGGGTTAAGTCCCGCAACGAGCGCAACCCTTGCCTTTAGTTGCCAGCATTAAGTTGGGCACTCTA

GAGGGACTGCCAGGGATAACCTGGAGGAAGGTGGGGATGACGTCAAATCATCATGCCCCTTATGCTTAGGGCTACACACG

TGCTACAATGGGTGGTACAGAGGGCAGCCAAGTCGTGAGGCGGAGCTAATCCCTTAAAGCCATTCTCAGTTCGGATTGTA

GGCTGAAACTCGCCTACATGAAGCTGGAGTTACTAGTAATCGCAGATCAGAATGCTGCGGTGAATGCGTTCCCGGGTCTT

GTACACACCGCCCGTCACACCATGGGAGTTGGGGGCGCCCGAAGCCGGTTAGCTAACCTTTTAGGAAGCGGCCGTCGAAG

GTGAAACCAATAACTGGGGTGAAGTCGT

>H4_3

GTATCCAACCTTCCGTTTACTCAGGGATAGCCTTTCGAAAGAAAGATTAATACCTGATAGTATGGTAAGATTGCATGATA

ATACCATTAAAGATTCATCGGTAAACGATGGGGATGCGTTCCATTAGGTAGTAGGCGGGGTAACGGCCCACCTAGCCGAC

GATGGATAGGGGTTCTGAGAGGAAGGTCCCCCACATTGGAACTGAGACACGGTCCAAACTCCTACGGGAGGCAGCAGTGA

GGAATATTGGTCAATGGGCGAGAGCCTGAACCAGCCAAGTAGCGTGAAGGATGAAGGTTCTATGGATTGTAAACTTCTTT

TATAAGGGAATAAAGTGCTTTACGTGTAGAGTTTTGTATGTACCTTATGAATAAGCATCGGCTAACTCCGTGCCAGCAGC

CGCGGTAATACGGAGGATGCGAGCGTTATCCGGATTTATTGGGTTTAAAGGGAGCGTAGACGGGATGTTAAGTCAGCTGT

GAAAGTTTGGGGCTCAACCTTAAAATTGCAGTTGAAACTGGCGTTCTTGAGTGCGGTAGAGGCAGGCGGAATTCGTGGTG

TAGCGGTGAAATGCTTAGATATCACGAAGAACCCCGATTGCGAAGGCAGCTTGCTGGAGCGTAACTGACGTTGATGCTCG

AAAGTGTGGGTATCAAACAGGATTAGATACCCTGGTAGTCCACACGGTAAACGATGGATACTCGCTGTTGGCGATATACG

GTCAGCGGCCAAGCGAAAGCATTAAGTATCCCACCTGGGGAGTACGCCGGCAACGGTGAAACTCAAAGGAATTGACGGGG

GCCCGCACAAGCGGAGGAACATGTGGTTTAATTCGATGATACGCGAGGAACCTTACCCGGGCTTAAATTATGCATGAATG

ATCTGGAGACAGATCAGCCGCAAGGCATGTATGAAGGTGCTGCATGGTTGTCGTCAGCTCGTGCCGTGAGGTGTCGGCTT

AAGTGCCATAACGAGCGCAACCCTTTCTGCCAGTTACTAACAGGCAATGCTGAGGACTCTGGCGGTACTGCCATCGTAAG

ATGTGAGGAAGGTGGGGATGACGTCAAATCAGCACGGCCCTTACGTCCGGGGCTACACACGTGTTACAATGGGGGGTACA

GAAGGCAGCTTACCGGCGACGGTTGGCCAATCCCTAAAGCCCCTCTCAGTTCGGACTGGAGTCTGCAACCCGACTCCACG

AAGCTGGATTCGCTAGTAATCGCGCATCAGCCACGGCGCGGTGAATACGTTCCCGGGCCTTGTACACACCGCCCGTCAAG

CCATGAAAGCCGGGAGTACCTGAAGTGCGTAACCGCGAGGAGCGCCCTAGGGTAACACTGGTAATTGGGGCTAAGTCGT

>H4_4

GTGGAACTATTAAAGAATTTCGGTCATCGATGGGGATGCGTTCCATTAGGTTGTTGGCGGGGTAACGGCCCACCAAGCCT

TCGATGGATAGGGGTTCTGAGAGGAAGGTCCCCCACATTGGAACTGAGACACGGTCCAAACTCCTACGGGAGGCAGCAGT

GAGGAATATTGGTCAATGGACGAGAGTCTGAACCAGCCAAGTAGCGTGAAGGATGACTGCCCTATGGGTTGTAAACTTCT

TTTATACGGGAATAAAGTGAGGCACGTGTGCCTTTTTGTATGTACCGTATGAATAAGGATCGGCTAACTCCGTGCCAGCA

GCCGCGGTAATACGGAGGATCCGAGCGTTATCCGGATTTATTGGGTTTAAAGGGAGCGTAGGCGGACGCTTAAGTCAGTT

GTGAAAGTTTGCGGCTCAACCGTAAAATTGCAGTTGATACTGGGTGTCTTGAGTACAGTAGAGGCAGGCGGAATTCGTGG

TGTAGCGGTGAAATGCTTAGATATCACGAAGAACTCCGATTGCGAAGGCAGCTTGCTGGACTGTAACTGACGCTGATGCT

CGAAAGTGTGGGTATCAAACAGGATTAGATACCCTGGTAGTCCACACAGTAAACGATGAATACTCGCTGTTTGCGATATA

CAGTAAGCGGCCAAGCGAAAGCGTTAAGTATTCCACCTGGGGAGTACGCCGGCAACGGTGAAACTCAAAGGAATTGACGG

GGGCCCGCACAAGCGGAGGAACATGTGGTTTAATTCGATGATACGCGAGGAACCTTACCCGGGCTTGAATTGCAACTGAA

TGATGTGGAGACATGTCAGCCGCAAGGCAGTTGTGAAGGTGCTGCATGGTTGTCGTCAGCTCGTGCCGTGAGGTGTCGGC

TTAAGTGCCATAACGAGCGCAACCCTTATCGATAGTTACCATCAGGTTATGCTGGGGACTCTGTCGAGACTGCCGTCGTA

AGATGTGAGGAAGGTGGGGATGACGTCAAATCAGCACGGCCCTTACGTCCGGGGCTACACACGTGTTACAATGGGGGGTA

CAGAAGGCAGCTACACGGCGACGTGATGCTAATCCCGAAAGCCTCTCTCAGTTCGGATTGGAGTCTGCAACCCGACTCCA

TGAAGCTGGATTCGCTAGTAATCGCGCATCAGCCACGGCGCGGTGAATACGTTCCCGGGCCTTGTACACACCGCCCGTCA

AGCCATGAAAGCCGGGGGTACCTGAAGTGCGTAACCGCGAGGAGCGCCCTAGGGTAAAACTGGTGATTGGGGCTAAGTC

>H4_5

GGGGCAGCATGAACTTAGCTTGCTAAGTTCGATGGCGACCGGCGCACCGTTGAGTAACGCGTATCCAACCTTCCGTACAC

TCAGGAATAGCCTTTCGAAAGAAAGATTAATACCTGATGGTATGATGGGATTGCATGAAATCATCATTAAAGATTCATCG

GTGTACGATGGGGATGCGTTCCATTAGATAGTAGGCGGGGTAACGGCCCACCTAGTCGACGATGGATAGGGGTTCTGAGA

GGAAGGTCCCCCACATTGGAACTGAGACACGGTCCAAACTCCTACGGGAGGCAGCAGTGAGGAATATTGGTCAATGGGCG

CGAGCCTGAACCAGCCAAGTAGCGTGAAGGATGAAGGTCCTACGGATTGTAAACTTCTTTTATAAGGGAATAAAGTCACC

CACGTGTGGGTGTTTGTATGTACCTTATGAATAAGCATCGGCTAACTCCGTGCCAGCAGCCGCGGTAATACGGAGGATGC

GAGCGTTATCCGGATTTATTGGGTTTAAAGGGAGCGTAGACGGGTCGTTAAGTCAGCTGTGAAAGTTCGGGGCTCAACCT

TGAAATTGCAGTTGATACTGGCGTCCTTGAGTACGGTTGAGGCAGGCGGAATTCGTGGTGTAGCGGTGAAATGCTTAGAT

ATCACGAAGAACCCCGATTGCGAAGGCAGCCTGCTAAACCGCCACTGACGTTGAGGCTCGAAAGTGTGGGTATCAAACAG

GATTAGATACCCTGGTAGTCCACACGGTAAACGATGGATACTCGCTGTTGGCGATAGACTGTCAGCGGCTTAGCGAAAGC

GTTAAGTATCCCACCTGGGGAGTACGCCGGCAACGGTGAAACTCAAAGGAATTGACGGGGGCCCGCACAAGCGGAGGAAC

ATGTGGTTTAATTCGATGATACGCGAGGAACCTTACCCGGGCTTGAATTGCAGACGAATTGCTTGGAAACAGGCAAGCCG

CAAGGCGTCTGTGAAGGTGCTGCATGGTTGTCGTCAGCTCGTGCCGTGAGGTGTCGGCTTAAGTGCCATAACGAGCGCAA

CCCTCGTGTCCAGTTGCTAGCAGGTAGTGCTGAGGACTCTGGACAGACTGCCATCGTAAGATGTGAGGAAGGTGGGGATG

ACGTCAAATCAGCACGGCCCTTACGTCCGGGGCTACACACGTGTTACAATGGGGGGTACAGCAGGCAGCTACCGGGCGAC

CGGATGCCAATCCCGAAAGCCTCTCTCAGTTCGGACTGGAGTCTGCAACCCGACTCCACGAAGCTGGATTCGCTAGTAAT

CGCGCATCAGCCACGGCGCGGTGAATACGTTCCCGGGCCTTGTACACACCGCCCGTCAAGCCATGAAAGCCGGGGGTACC

TGAAGTGCGTAACCGCAAGGAGCGCCCTAGGGTAAAACTGGTAATT

>H4_10

GGAAGCGAGCGGCGAACGGCTGAGTAACACGTGACCAACCTGCCCTGCGCACGGGGACAGCCGCGGGAAACCGCGGGTGA

TACCCGGTGGCCCCCTGGGACCGCATGGCCCCAGGGGCATAGCTCCGGCGGCGCGGGATGGGGTCGCGGCCCATCAGGTA

GTTGGTAGGGTGACGGCCTACCAAGCCGACTACGGGTAGCCGGGTTGAGAGACCGACCGGCCAGATTGGGACTGAGACAC

GGCCCAGACTCCTACGGGAGGCAGCAGTGGGGAATCTTGCGCAATGGGGGGAACCCTGACGCAGCGACGCCGCGTGCGGG

ATGGAGGCCTTCGGGTCGTGAACCGCTTTCAGCAGGGACGAGACTGACGGTACCTGCAGAAGAAGCCCCGGCTAACTACG

TGCCAGCAGCCGCGGTAATACGTAGGGGGCGAGCGTTATCCGGATTCATTGGGCGTAAAGCGCGCGTAGGCGGCCGCGCA

GGCGGGGGGTCAAATCCCGGGGCTCAACCCCGGGCCGCCCCCCGAACCGCGCGGCTTGGGTCCGGCAGGGGAGGGTGGAA

CGCCCGGTGTAGCGGTGGAATGCGCAGATATCGGGCGGAACACCGGTGGCGAAGGCGGCCCTCTGGGCCGGCACCGACGC

TGAGGCGCGAGAGCCGGGGGAGCGAACAGGATTAGATACCCTGGTAGTCCCGGCCGTAAACGATGGACGCTAGGTGTGGG

GACATATGAGTCTCCGTGCCGCAGCCAACGCATTAAGCGTCCCGCCTGGGGAGTACGGCCGCAAGGCTAAAACTCAAAGG

AATTGACGGGGGCCCGCACAAGCAGCGGAGCATGTGGCTTAATTCGAAGCAACGCGAAGAACCTTACCCGCGCTTGACAT

GGCGGTGAAGCGGCGGAGACGCCGTGGCCGAGAGGAGCCGCCACAGGTGGTGCATGGCTGTCGTCAGCTCGTGTCGTGAG

ATGTTGGGTTAAGTCCCGCAACGAGCGCAACCCCCGCCGCGTGTTGCCATCGGGTGATGCCGGGAACCCACGCGGGACCG

CCGCCGCCAAGGCGGAGGAGGGCGGGGACGACGTCAAGTCATCATGCCCCTTATGCGCGGGGCTGCACACGTGCTACAAT

GGCAGGTACAGAGGGTTGCCACCCCGCGAGGGGGAGCGGATCCCGAAAGCCTGCCCCAGTTCGGACCGGGGGCTGCAACC

CGCCCCCGCGAAGTCGGAGTTGCTAGTAATCGCGGATCAGCATGCCGCGGTGAATGCGTTCCCGGGCCTTGTACACACCG

CCCGTCACACCACCCGAGTCGTCTGCACCCGAAGCCGCCGGCCCAACCTCGTGAGGGAGGCGTCGAAGGTGTGGAGGGCG

AGGGGGGTGAAGTCGTAACA

>H4_16

GGTAGCTAAAACCGGATAGGTATACAGAGCGCATGCTCAGTATATTAAAGCGCCCATCAAGGCGTGAACATGGATGGACC

TGCGGCGCATTAGCTAGTTGGTGAGGTAACGGCCCACCAAGGCGATGATGCGTAGCCGGCCTGAGAGGGTAAACGGCCAC

ATTGGGACTGAGACACGGCCCAAACTCCTACGGGAGGCAGCAGTAGGGAATTTTCGTCAATGGGGGAAACCCTGAACGAG

CAATGCCGCGTGAGTGAAGAAGGTCTTCGGATCGTAAAGCTCTGTTGTAAGTGAAGAACGGCTCATAGAGGAAATGCTAT

GGGAGTGACGGTAGCTTACCAGAAAGCCACGGCTAACTACGTGCCAGCAGCCGCGGTAATACGTAGGTGGCAAGCGTTAT

CCGGAATCATTGGGCGTAAAGGGTGCGTAGGTGGCGTACTAAGTCTGTAGTAAAAGGCAATGGCTCAACCATTGTAAGCT

ATGGAAACTGGTATGCTGGAGTGCAGAAGAGGGCGATGGAATTCCATGTGTAGCGGTAAAATGCGTAGATATATGGAGGA

ACACCAGTGGCGAAGGCGGTCGCCTGGTCTGTAACTGACACTGAGGCACGAAAGCGTGGGGAGCAAATAGGATTAGATAC

CCTAGTAGTCCACGCCGTAAACGATGAGAACTAAGTGTTGGAGGAATTCAGTGCTGCAGTTAACGCAATAAGTTCTCCGC

CTGGGGAGTATGCACGCAAGTGTGAAACTCAAAGGAATTGACGGGGGCCCGCACAAGCGGTGGAGTATGTGGTTTAATTC

GAAGCAACGCGAAGAACCTTACCAGGCCTTGACATGGAAACAAATACCCTAGAGATAGGGGGATAATTATGGATCACACA

GGTGGTGCATGGTTGTCGTCAGCTCGTGTCGTGAGATGTTGGGTTAAGTCCCGCAACGAGCGCAACCCTTGTCGCATGTT

ACCAGCATCAAGTTGGGGACTCATGCGAGACTGCCGGTGACAAACCGGAGGAAGGTGGGGATGACGTCAAATCATCATGC

CCCTTATGGCCTGGGCTACACACGTACTACAATGGCGACCACAAAGAGCAGCGACACAGTGATGTGAAGCGAATCTCATA

AAGGTCGTCTCAGTTCGGATTGAAGTCTGCAACTCGACTTCATGAAGTCGGAATCGCTAGTAATCGCAGATCAGCATGCT

GCGGTGAATACGTTCTCGGGCCTTGTACACACCGCCCGTCAAACCATGGGAGTCAGTAATACCCGAAGCCGGTGGCATAA

CCGTAAGGAGTGAGCCGTCGAAGGTAGGACCGATGACTGGGGTTAAGTCGTAACAGGGTAACC

>H4_17

ACGCGTAAGCAACCTGCCCTCCGGATGGGGACAACAGCTGGAAACGGCTGCTAATACCGAATACGTTTCCATTGCCGCAT

GGCAGTGGGAAGAAAGGTGGCCTCTGAATATGCTACCGCCGGGGGAGGGGCTTGCGTCTGATTAGCTAGTTGGAGGGGTA

ACGGCCCACCAAGGCGACGATCAGTAGCCGGTCTGAGAGGATGAACGGCCACATTGGAACTGAGACACGGTCCAGACTCC

TACGGGAGGCAGCAGTGGGGAATCTTCCGCAATGGGCGAAAGCCTGACGGAGCAACGCCGCGTGAGCGAAGACGGCCTTC

GGGTTGTAAAGCTCTGTTATACGGGACGAACGGCTAGTGTGCCAATACCACATTAGAATGACGGTACCGTAAGAGAAAGC

CACGGCTAACTACGTGCCAGCAGCCGCGGTAATACGTAGGTGGCAAGCGTTGTCCGGAATTATTGGGCGTAAAGGGCGCG

CAGGCGGTTTCATAAGTCTGTCTTAAAAGTGCGGGGCTTAACCCCGTGAGGGGACGGAAACTGTGAGACTGGAGTGTCGG

AGAGGAAAGCGGAATTCCTAGTGTAGCGGTGAAATGCGTAGATATTAGGAGGAACACCAGTGGCGAAAGCGGCTTTCTGG

ACGACAACTGACGCTGAGGCGCGAAAGCCAGGGGAGCGAACGGGATTAGATACCCCGGTAGTCCTGGCCGTAAACGATGG

ATACTAGGTGTAGGGGGTATCGACCCCTCCTGTGCCGGAGTTAACGCAATAAGTATCCCGCCTGGGGAGTACGGCCGCAA

GGCTGAAACTCAAAGGAATTGACGGGGGCCCGCACAAGCGGTGGAGTATGTGGTTTAATTCGACGCAACGCGAAGAACCT

TACCAAGCCTTGACATTGAGTGCTATCCTCAGAGATGAGGAGTTCTTCTTCGGAAGACGCGAAAACAGGTGGTGCACGGC

TGTCGTCAGCTCGTGTCGTGAGATGTTGGGTTAAGTCCCGCAACGAGCGCAACCCCTATCTTCTGTTGCCAGCGCGTCAT

GGCGGGGACTCAGGAGAGACTGCCGCAGACAATGCGGAGGAAGGCGGGGATGACGTCAAGTCATCATGCCCCTTATGGCT

TGGGCTACACACGTACTACAATGGCTCTTAATAGAGGGAAGCGAAGGAGCGATCCGGAGCAAACCCCAAAAACAGAGTCC

CAGTTCGGATTGCAGGCTGCAACCCGCCTGCATGAAGCAGGAATCGCTAGTAATCGCAGGTCAGCATACTGCGGTGAATA

CGTTCCCGGGCCTTGTACACACCGCCCGTCACACCACGAAAGTCATTCACACCCGAAGCCGGTGAGGTAACCGTAAGGAG

CCAGCCGTCGAAGGTGGGGGCGATGATTGGGGTGAAGTCGTAA

>H4_30

TGATTGACTGAGTGGCGGACGGGTGAGTAACGCGTGGATAACCTGCCTCACACTGGGGGATAACAGTTAGAAATGACTGC

TAATACCGCATAAGCGCACAGTGCCGCATGGCAGTGTGTGAAAAACTCCGGTGGTGTGAGATGGATCCGCGTCTGATTAG

CCAGTTGGCGGGGTAACGGCCCACCAAAGCGACGATCAGTAGCCGACCTGAGAGGGTGACCGGCCACATTGGGACTGAGA

CACGGCCCAAACTCCTACGGGAGGCAGCAGTGGGGAATATTGCACAATGGGCGAAAGCCTGATGCAGCGACGCCGCGTGA

GTGAAGAAGTATTTCGGTATGTAAAGCTCTATCAGCAGGGAAGAAAATGACGGTACCTGACTAAGAAGCCCCGGCTAACT

ACGTGCCAGCAGCCGCGGTAATACGTAGGGGGCAAGCGTTATCCGGATTTACTGGGTGTAAAGGGAGCGTAGACGGCGAA

GCAAGTCTGAAGTGAAAACCCAGGGCTCAACCCTGGGACTGCTTTGGAAACTGTTTTGCTAGAGTGTCGGAGAGGTAAGT

GGAATTCCTAGTGTAGCGGTGAAATGCGTAGATATTAGGAGGAACACCAGTGGCGAAGGCGGCTTACTGGACGATAACTG

ACGTTGAGGCTCGAAAGCGTGGGGAGCAAACAGGATTAGATACCCTGGTAGTCCACGCCGTAAACGATGAATGCTAGGTG

TTGGGGGGCAAAGCCCTTCGGTGCCGCCGCAAACGCAGTAAGCATTCCACCTGGGGAGTACGTTCGCAAGAATGAAACTC

AAAGGAATTGACGGGGACCCGCACAAGCGGTGGAGCATGTGGTTTAATTCGAAGCAACGCGAAGAACCTTACCAAGTCTT

GACATCCCCCTGACGGGCCGGTAACGCGGCCTTTCCTTCGGGACAGGGGAGACAGGTGGTGCATGGTTGTCGTCAGCTCG

TGTCGTGAGATGTTGGGTTAAGTCCCGCAACGAGCGCAACCCTTATCCTTAGTAGCCAGCACGTGAAGGTGGGCACTCTA

GGGAGACTGCCAGGGATAACCTGGAGGAAGGTGGGGATGACGTCAAATCATCATGCCCCTTATGATTTGGGCTACACACG

TGCTACAATGGCGTAAACAAAGGGAAGCGAGACAGTGATGTGGAGCAAATCCCAAAAATAACGTCCCAGTTCGGACTGTA

GTCTGCAACCCGACTACACGAAGCTGGAATCGCTAGTAATCGCGAATCAGAATGTCGCGGTGAATACGTTCCCGGGTCTT

GTACACACCGCCCGTCACACCATGGGAGTCAGCAACGCCCGAAGTCAGTGACCCAACCGAAAGGAGGGAGCTGCCGAAGG

CGGGGCAGGTAACTGGGGTGAAGTCGTA

>H4_33

CTTCGGATGAAGACTTTTGTGACTGAGCGGCGGACGGGTGAGTAACGCGTGGGTAACCTGCCTCATACAGGGGGATAACA

GTTAGAAATGGCTGCTAATACCGCATAAGACCACAGTACTGCATGGTACAGTGGTAAAAACTCCGGTGGTATGAGATGGA

CCCGCGTCTGATTAGGTAGTTGGTGAGGTAACGGCCCACCAAGCCGACGATCAGTAGCCGACCTGAGAGGGTGACCGGCC

ACATTGGGACTGAGACACGGCCCAGACTCCTACGGGAGGCAGCAGTGGGGAATATTGCACAATGGGCGAAAGCCTGATGC

AGCGACGCCGCGTGAAGGATGAAGTATTTCGGTATGTAAACTTCTATCAGCAGGGAAGAAAATGACGGTACCTGACTAAG

AAGCCCCGGCTAACTACGTGCCAGCAGCCGCGGTAATACGTAGGGGGCAAGCGTTATCCGGATTTACTGGGTGTAAAGGG

AGCGTAGACGGCTGTGCAAGTCTGAAGTGAAAGGCATGGGCTCAACCTGTGGACTGCTTTGGAAACTGTGCAGCTAGAGT

GTCGGAGAGGTAAGTGGAATTCCTAGTGTAGCGGTGAAATGCGTAGATATTAGGAGGAACACCAGTGGCGAAGGCGGCTT

ACTGGACGATGACTGACGTTGAGGCTCGAAAGCGTGGGGAGCAAACAGGATTAGATACCCTGGTAGTCCACGCCGTAAAC

GATGACTGCTAGGTGTCGGGTAGCAAAGCTATTCGGTGCCGCAGCTAACGCAATAAGCAGTCCACCTGGGGAGTACGTTC

GCAAGAATGAAACTCAAAGGAATTGACGGGGACCCGCACAAGCGGTGGAGCATGTGGTTTAATTCGAAGCAACGCGAAGA

ACCTTACCTGATCTTGACATCCCGATGACCGCTTCGTAATGGAAGCTTTTCTTCGGAACATCGGTGACAGGTGGTGCATG

GTTGTCGTCAGCTCGTGTCGTGAGATGTTGGGTTAAGTCCCGCAACGAGCGCAACCCTTATCTTCAGTAGCCAGCATTTA

GGATGGGCACTCTGGAGAGACTGCCAGGGATAACCTGGAGGAAGGTGGGGATGACGTCAAATCATCATGCCCCTTATGAC

CAGGGCTACACACGTGCTACAATGGCGTAAACAAAGGGAAGCAGAGCCGCGAGGCCGAGCAAATCTCAAAAATAACGTCT

CAGTTCGGATTGTAGTCTGCAACTCGACTACATGAAGCTGGAATCGCTAGTAATCGCAGATCAGAATGCTGCGGTGAATA

CGTTCCCGGGTCTTGTACACACCGCCCGTCACACCATGGGAGTCAGTAACGCCCGAAGTCAGTGACCCAACCGAAAGGAG

GGAGCTGCCGAAGGTGGGACCGATAACTGGGGTGAAGTCGTAACAAGGTAACCG

>H4_42

GACGGGTGAGTAACGCGTGGGTAACCTGCCTTGTACTGGGGGATAGCAGCTGGAAACGGCTGGTAATACCGCATAAGCGC

ACAATGTTGCATGACATGGTGTGAAAAACTCCGGTGGTATAAGATGGACCCGCGTCTGATTAGCTAGTTGGTGAGATAAC

AGCCCACCAAGGCGACGATCAGTAGCCGACCTGAGAGGGTGACCGGCCACATTGGGACTGAGACACGGCCCAGACTCCTA

CGGGAGGCAGCAGTGGGGAATATTGCACAATGGAGGAAACTCTGATGCAGCGACGCCGCGTGAGTGAAGAAGTAATTCGT

TATGTAAAGCTCTATCAGCAGGGAAGATAGTGACGGTACCTGACTAAGAAGCTCCGGCTAAATACGTGCCAGCAGCCGCG

GTAATACGTATGGAGCAAGCGTTATCCGGATTTACTGGGTGTAAAGGGAGTGTAGGTGGCCATGCAAGTCAGAAGTGAAA

ATCCGGGGCTCAACCCCGGAACTGCTTTTGAAACTGTAAGGCTAGAGTGCAGGAGGGGTGAGTGGAATTCCTAGTGTAGC

GGTGAAATGCGTAGATATTAGGAGGAACACCAGTGGCGAAGGCGGCTCACTGGACTGTAACTGACACTGAGGCTCGAAAG

CGTGGGGAGCAAACAGGATTAGATACCCTGGTAGTCCACGCCGTAAACGATGAATACTAGGTGTCGGGGCCCAAAAGGGC

TTCGGTGCCGCAGCAAACGCAATAAGTATTCCACCTGGGGAGTACGTTCGCAAGAATGAAACTCAAAGGAATTGACGGGG

ACCCGCACAAGCGGTGGAGCATGTGGTTTAATTCGAAGCAACGCGAAGAACCTTACCAAGTCTTGACATCCCACTGACCG

GACAGTAATGTGTCCTTTCCTTCGGGACAGTGGAGACAGGTGGTGCATGGTTGTCGTCAGCTCGTGTCGTGAGATGTTGG

GTTAAGTCCCGCAACGAGCGCAACCCCTATCCTTAGTAGCCAGCAGTAAGATGGGCACTCTAGGGAGACTGCCAGGGATA

ACCTGGAGGAAGGTGGGGATGACGTCAAATCATCATGCCCCTTATGACTTGGGCTACACACGTGCTACAATGGCGTAAAC

AAAGTGAAGCGAAGTCGTGAGGCCAAGCAAATCACAAAAATAACGTCTCAGTTCGGATTGTAGTCTGCAACTCGACTACA

TGAAGCTGGAATCGCTAGTAATCGCAGATCAGAATGCTGCGGTGAATACGTTCCCGGGTCTTGTACACACCGCCCGTCAC

ACCATGGGAGTCGAAAATGCCCGAAGTCGGTGACCTAACGAAAGAAGGAGCCGCCGAAGGCAGGTTTGATAACTGGGGTG

AAGTCGTAACAAGGTAACCG

>H4_59

AGTCGACGGAGCACCTTGACTGAGGTTTCGGCCAAATGATAGGAATGCTTAGTGGCGGACTGGTGAGTAACGCGTGAGGA

ACCTGCCTTCCAGAGGGGGACAACAGTTGGAAACGACTGCTAATACCGCATGACGCATGACCGGGGCATCCCGGGCATGT

CAAAGATTTTATCGCTGGAAGATGGCCTCGCGTCTGATTAGCTAGATGGTGGGGTAACGGCCCACCATGGCGACGATCAG

TAGCCGGACTGAGAGGTTGACCGGCCACATTGGGACTGAGATACGGCCCAGACTCCTACGGGAGGCAGCAGTGGGGAATA

TTGGGCAATGGACGCAAGTCTGACCCAGCAACGCCGCGTGAAGGAAGAAGGCTTTCGGGTTGTAAACTTCTTTTGTCAGG

GAAGAGTAGAAGACGGTACCTGACGAATAAGCCACGGCTAACTACGTGCCAGCAGCCGCGGTAATACGTAGGTGGCAAGC

GTTGTCCGGATTTACTGGGTGTAAAGGGCGTGCAGCCGGGCCGGCAAGTCAGATGTGAAATCTGGAGGCTTAACCTCCAA

ACTGCATTTGAAACTGTAGGTCTTGAGTACCGGAGAGGTTATCGGAATTCCTTGTGTAGCGGTGAAATGCGTAGATATAA

GGAAGAACACCAGTGGCGAAGGCGGATAACTGGACGGCAACTGACGGTGAGGCGCGAAAGCGTGGGGAGCAAACAGGATT

AGATACCCTGGTAGTCCACGCTGTAAACGATGGATACTAGGTGTGCGGGGACTGACCCCCTGCGTGCCGCAGTTAACACA

ATAAGTATCCCACCTGGGGAGTACGATCGCAAGGTTGAAACTCAAAGGAATTGACGGGGGCCCGCACAAGCGGTGGATTA

TGTGGTTTAATTCGAAGCAACGCGAAGAACCTTACCAGGGCTTGACATCCTACTAACGAAGTAGAGATACATTAGGTGCC

CTTCGGGGAAAGTAGAGACAGGTGGTGCATGGTTGTCGTCAGCTCGTGTCGTGAGATGTTGGGTTAAGTCCCGCAACGAG

CGCAACCCCTATTGTTAGTTGCTACGCAAGAGCACTCTAGCGAGACTGCCGTTGACAAAACGGAGGAAGGTGGGGACGAC

GTCAAATCATCATGCCCCTTATGTCCTGGGCTACACACGTAATACAATGGCGGTCAACAGAGGGAGGCAAAGCCGCGAGG

CAGAGCAAACCCCCAAAAGCCGTCCCAGTTCGGATCGCAGGCTGCAACCCGCCTGCGTGAAGTCGGAATCGCTAGTAATC

GCGGATCAGCATGCCGCGGTGAATACGTTCCCGGGCCTTGTACACACCGCCCGTCACACCATGAGAGTCGGGAACACCCG

AAGTCCGTAGCCTAACCGCAAGGAGGGCGCGGCCGAAGGTGGGTTCGATAATTGGGGTGAAGTCGTAC

>H4_64

AGTCGACGGACACATCCGACGGAATAGCTTGCTAGGAAGATGGATGTTGTTAGTGGCGGACGGGTGAGTAACACGTGAGC

AACCTACCTCAGAGTGGGGGACAACAGTTGGAAACGACTGCTAATACCGCATAAGATGGCAGGGTCGCATGGCCTGGTCA

TAAAAGGAGCAATTCGCTCTGAGATGGGCTCGCGTCTGATTAGCTAGTTGGTGAGGTAACGGCTCACCAAGGCAACGATC

AGTAGCCGGACTGAGAGGTTGAACGGCCACATTGGGACTGAGACACGGCCCAGACTCCTACGGGAGGCAGCAGTGGGGAA

TATTGCACAATGGGGGAAACCCTGATGCAGCGACGCCGCGTGAGGGAAGACGGTTTTCGGATTGTAAACCTCTGTCTTGT

GGGACGATAGTGACGGTACCACAGGAGGAAGCCATGGCTAACTACGTGCCAGCAGCCGCGGTAATACGTAGATGGCGAGC

GTTGTCCGGAATTACTGGGTGTAAAGGGAGTGTAGGCGGGCTGGTAAGTTGAATGTGAAACCTTCGGGCTCAACCCGGAG

CGTGCGTTCAAAACTGCTGGTCTTGAGTGAAGTAGAGGCAGGCGGAATTCCCGGTGTAGCGGTGGAATGCGTAGATATCG

GGAGGAACACCAGTGGCGAAGGCGGCCTGCTGGGCTTTTACTGACGCTGAGGCTCGAAAGCATGGGTAGCAAACAGGATT

AGATACCCTGGTAGTCCATGCCGTAAACGATGATTACTAGGTGTGGGGGGATTGACCCCCTCCGTGCCGGAGTTAACACA

ATAAGTAATCCACCTGGGGAGTACGACCGCAAGGTTGAAACTCAAAGGAATTGACGGGGGCCCGCACAAGCAGTGGAGTA

TGTGGTTTAATTCGAAGCAACGCGAAAAACCTTACCAGGTCTTGACATCCATCGCCAGGCTAAGAGATTAGCTGTTCCCT

CCGGGGACGATGAGACAGGTGGTGCATGGTTGTCGTCAGCTCGTGTCGTGAGATGTTGGGTTAAGTCCCGCAACGAGCGC

AACCCTTACTATTAGTTGCTACGCAAGAGCACTCTAATGGGACTGCCGTTGACAAAACGGAGGAAGGTGGGGATGACGTC

AAATCATCATGCCCCTTATGACCTGGGCTACACACGTACTACAATGGCCGTTAACAGAGAGCAGCGATACCGCGAGGTGG

AGCGAATCTAGAAAAACGGTCTCAGTTCGGATTGCAGGCTGAAACTCGCCTGCATGAAGTCGGAATTGCTAGTAATCGCG

GATCAGCATGCCGCGGTGAATACGTTCCCGGGCCTTGTACACACCGCCCGTCACACCATGAGAGCCGGTAACACCCGAAG

TCAGTAGCCTAACCGCAAGGAGGGCGCTGCCGAAGGTGGGGCTGGTAATTGGGGTGAAGTCGTAAC_

>H1_15

AGCGGCGGACGGGTGAGTAACGCGTGGGTAACCTGCCCTGTACACACGGATAACATACCGAAAGGTATGCTAATACGGGA

TAACATAAGAAATTCGCATGTTTTTCTTATCAAAGCTCCGGCGGTACAGGATGGACCCGCGTCTGATTAGCTAGTTGGTG

AGGTAACGGCTCACCAAGGCGACGATCAGTAGCCGACCTGAGAGGGTGATCGGCCACATTGGAACTGAGACACGGTCCAA

ACTCCTACGGGAGGCAGCAGTGGGGAATATTGCACAATGGGCGAAAGCCTGATGCAGCAACGCCGCGTGAGCGATGAAGG

CCTTCGGGTCGTAAAGCTCTGTCCTCAAGGAAGATAATGACGGTACTTGAGGAGGAAGCCCCGGCTAACTACGTGCCAGC

AGCCGCGGTAATACGTAGGGGGCTAGCGTTATCCGGATTTACTGGGCGTAAAGGGTGCGTAGGCGGTCTTTTAAGTCAGG

AGTGAAAGGCTACGGCTCAACCGTAGTAAGCTCTTGAAACTGGAGGACTTGAGTGCAGGAGAGGAGAGTGGAATTCCTAG

TGTAGCGGTGAAATGCGTAGATATTAGGAGGAACACCAGTAGCGAAGGCGGCTCTCTGGACTGTAACTGACGCTGAGGCA

CGAAAGCGTGGGGAGCAAACAGGATTAGATACCCTGGTAGTCCACGCCGTAAACGATGAGTACTAGCTGTCGGAGGTTAC

CCCCTTCGGTGGCGCAGCTAACGCATTAAGTACTCCGCCTGGGGAGTACGCTCGCAAGAGTGAAACTCAAAGGAATTGAC

GGGGACCCGCACAAGTAGCGGAGCATGTGGTTTAATTCGAAGCAACGCGAAGAACCTTACCTAAGCTTGACATCCTTTTG

ACCGATGCCTAATCGCATCTTTCCCTTCGGGGACAGAAGTGACAGGTGGTGCATGGTTGTCGTCAGCTCGTGTCGTGAGA

TGTTGGGTTAAGTCCCGCAACGAGCGCAACCCTTGCCTTTAGTTGCCATCATTAAGTTGGGCACTCTAGAGGGACTGCCA

GGGATAACCTGGAGGAAGGTGGGGATGACGTCAAATCATCATGCCCCTTATGCTTAGGGCTACACACGTGCTACAATGGG

TGGTACAGAGGGCAGCGAAGTCGTGAGGCCAAGCTAATCCCTTAAAGCCATTCTCAGTTCGGATTGTAGGCTGAAACTCG

CCTACATGAAGCTGGAGTTACTAGTAATCGCAGATCAGAATGCTGCGGTGAATGCGTTCCCGGGTCTTGTACACACCGCC

CGTCACACCATGGGAGTTGGGGGCGCCCGAAGCCGGCTAGCTAACCTTTTGGAAGCGGTCGTCGAAGGTGAAACCAATAA

CTGGGGTGAAGTCGT

>H5_6

AGTCGAGGGGCAGCCAGGTAGCATACCGGGTGGCGACCGGCGCACGGGTGAGTAACGCGTATGCAACTTACCTATCAGAG

GGGGATAACCCGGCGAAAGTCGGACTAATACCGCATGAAGCAGGGGCCCCGCATGGGGATATTTGCTAAAGATTCATCGC

TGATAGATAGGCATGCGTTCCATTAGGCAGTTGGCGGGGTAACGGCCCACCAAACCGACGATGGATAGGGGTTCTGAGAG

GAAGGTCCCCCACATTGGTACTGAGACACGGACCAAACTCCTACGGGAGGCAGCAGTGAGGAATATTGGTCAATGGGCGT

AAGCCTGAACCAGCCAAGTCGCGTGAGGGATGAAGGCTCTATGGGTCGTAAACCTCTTTTATAAGGGAATAAAGTGCGGG

ACGTGTCCCGTTTTGTATGTACCTTATGAATAAGGATCGGCTAACTCCGTGCCAGCAGCCGCGGTAATACGGAGGATCCG

AGCGTTATCCGGATTTATTGGGTTTAAAGGGTGCGTAGGCGGCCTTTTAAGTCAGCGGTGAAAGTCTGTGGCTCAACCAT

AGAATTGCCGTTGAAACTGGGGGGCTTGAGTATGTTTGAGGCAGGCGGAATGCGTGGTGTAGCGGTGAAATGCTTAGATA

TCACGCAGAACCCCGATTGCGAAGGCAGCCTGCCAAGCCATGACTGACGCTGATGCACGAAAGCGTGGGGATCAAACAGG

ATTAGATACCCTGGTAGTCCACGCAGTAAACGATGATCACTAGCTGTTTGCGATACAGTGTAAGCGGCACAGCGAAAGCG

TTAAGTGATCCACCTGGGGAGTACGCCGGCAACGGTGAAACTCAAAGGAATTGACGGGGGCCCGCACAAGCGGAGGAACA

TGTGGTTTAATTCGATGATACGCGAGGAACCTTACCCGGGTTTGAACGCATTTGGACCGATCTGGAAACAGATCTTCTAG

CAATAGCGATTTGCGAGGTGCTGCATGGTTGTCGTCAGCTCGTGCCGTGAGGTGTCGGCTTAAGTGCCATAACGAGCGCA

ACCCTTGCCACTAGTTACTAACAGGTGATGCTGAGGACTCTGGTGGGACTGCCAGCGTAAGCTGCGAGGAAGGCGGGGAT

GACGTCAAATCAGCACGGCCCTTACATCCGGGGCGACACACGTGTTACAATGGCGTGGACAAAGGGAAGCCACCTGGCGA

CAGGGAGCGAATCCCCAAACCACGTCTCAGTTCGGATCGGAGTCTGCAACCCGACTCCGTGAAGCTGGATTCGCTAGTAA

TCGCGCATCAGCCATGGCGCGGTGAATACGTTCCCGGGCCTTGTACACACCGCCCGTCAAGCCATGGGAGCCGGGGGTAC

CTGAAGTCCGTAACCGCGAGGATCGGCCTAGGGTAAAACTGGTGACTGGGGCTAAGTCGT

>H5_13

CCTGCCCTGTACACACGGATAACATACCGAAAGGTATGCTAATACGGGATAACATAAGAAATTCGCATGTTTTTCTTATC

AAAGCTCCGGCGGTACAGGATGGACCCGCGTCTGATTAGCTAGTTGGTGAGGTAACGGCTCACCAAGGCGACGATCAGTA

GCCGACCTGAGAGGGTGATCGGCCACATTGGAACTGAGACACGGTCCAAACTCCTACGGGAGGCAGCAGTGGGGAATATT

GCACAATGGGCGAAAGCCTGATGCAGCAACGCCGCGTGAGCGATGAAGGCCTTCGGGTCGTAAAGCTCTGTCCTCAAGGA

AGATAATGACGGTACTTGAGGAGGAAGCCCCGGCTAACTACGTGCCAGCAGCCGCGGTAATACGTAGGGGGCTAGCGTTA

TCCGGATTTACTGGGCGTAAAGGGTGCGTAGGCGGTCTTTTAAGTCAGGAGTGAAAGGCTACGGCTCAACCGTAGTAAGC

TCTTGAAACTGGAGGACTTGAGTGCAGGAGAGGAGAGTGGAATTCCTAGTGTAGCGGTGAAATGCGTAGATATTAGGAGG

AACACCAGTAGCGAAGGCGGCTCTCTGGACTGTAACTGACGCTGAGGCACGAAAGCGTGGGGAGCAAACAGGATTAGATA

CCCTGGTAGTCCACGCCGTAAACGATGAGTACTAGCTGTCGGAGGTTACCCCCTTCGGTGGCGCAGCTAACGCATTAAGT

ACTCCGCCTGGGGAGTACGCTCGCAAGAGTGAAACTCAAAGGAATTGACGGGGACCCGCACAAGTAGCGGAGCATGTGGT

TTAATTCGAAGCAACGCGAAGAACCTTACCTAAGCTTGACATCCTTTTGACCGATGCCTAATCGCATTTTTCCCTTCGGG

GACAGAAGTGACAGGTGGTGCATGGTTGTCGTCAGCTCGTGTCGTGAGATGTTGGGTTAAGTCCCGCAACGAGCGCAACC

CTTGCCTTTAGTTGCCATCATTAAGTTGGGCACTCTAGAGGGACTGCCAGGGATAACCTGGAGGAAGGTGGGGATGACGT

CAAATCATCATGCCCCTTATGCTTAGGGCTACACACGTGCTACAATGGGTGGTACAGAGGGCAGCGAAGTCGTGAGGCCA

AGCTAATCCCTTAAAGCCATTCTCAGTTCGGATTGTAGGCTGAAACTCGCCTACATGAAGCTGGAGTTACTAGTAATCGC

AGATCAGAATGCTGCGGTGAATGCGTTCCCGGGTCTTGTACACACCGCCCGTCACACCATGGGAGTTGGGGGCGCCCGAA

GCCGGCTAGCTAACCTTTT

>H5_17

GGGTAACCTGCCTCATAGAGGGGAATAGCCTCCCGAAAGGGAGATTAATACCGCATAACATTGCAGTTTCGCATGAAACA

GCAATTAAAGGAGCAATCCGCTATGAGATGGACCCGCGGCGCATTAGCTAGTTGGTAAGGTAATGGCTTACCAAGGCGAC

GATGCGTAGCCGACCTGAGAGGGTGATCGGCCACATTGGGACTGAGACACGGCCCAGACTCCTACGGGAGGCAGCAGTGG

GGAATATTGCACAATGGGGGAAACCCTGATGCAGCAACGCCGCGTGAGTGATGACGGTCTTCGGATTGTAAAGCTCTGTC

TTTGGGGACGATAATGACGGTACCCAAGGAGGAAGCCACGGCTAACTACGTGCCAGCAGCCGCGGTAATACGTAGGTGGC

GAGCGTTGTCCGGATTTACTGGGCGTAAAGGGAGCGTAGGCGGATTTTTAAGTGGGATGTGAAATACCCGGGCTCAACCT

GGGTGCTGCATTCCAAACTGGGAATCTAGAGTGCAGGAGGGGAGAGTGGAATTCCTAGTGTAGCGGTGAAATGCGTAGAG

ATTAGGAAGAACACCAGTGGCGAAGGCGACTCTCTGGACTGTAACTGACGCTGAGGCTCGAAAGCGTGGGGAGCGAACAG

GATTAGATACCCTGGTAGTCCACGCCGTAAACGATGAATACTAGGTGTAGGGGTTTCAACACCTCTGTGCCGCCGCTAAC

GCATTAAGTATTCCGCCTGGGGAGTACGGTCGCAAGATTAAAACTCAAAGGAATTGACGGGGGCCCGCACAAGTAGCGGA

GCATGTGGTTTAATTCGAAGCAACGCGAAGAACCTTACCTAGACTTGACATCCTCTGCATTACCCTTAATCGGGGAAGTT

CCTTCGGGAACAGAGTGACAGGTGGTGCATGGTTGTCGTCAGCTCGTGTCGTGAGATGTTGGGTTAAGTCCCGCAACGAG

CGCAACCCCTATTGTTAGTTGCTACCATTAAGTTGAGCACTCTAGCGAGACTGCCTGGGTTAACCAGGAGGAAGGTGGGG

ATGACGTCAAATCATCATGCCCCTTATGTCTAGGGCTACACACGTGCTACAATGGCAAGTACAGAGAGATGCAATACCGC

GAGGTGGAGCTAAACTTCAAAACTTGTCTCAGTTCGGATTGTAGGCTGAAACTCGCCTACATGAAGCTGGAGTTACTAGT

AATCGCGAATCAGCATGTCGCGGTGAATACGTTCCCGGGCCTTGTACACACCGCCCGTCACACCATGAGAGTTGGCAATA

CCCAAAGTTCGTGAGCTAACGCGTAAGCGAGGCAGCGACCTAAGGTAGGGTCAGCGATTGGGGTGAAGTCG

>H5_19

ATGCGTTTGCTGCGGCACCGAAGCCTCTACGGACCCGACACCTAGTATTCATCGATTACGGGGAGGACTACCAGGGTATC

TAATCCTGTTTGCTCCCCACGCTTTCGTGCCTCAGTGTCAGTTTCAGTCCAGTAAGCCGCCTTCGCCACTGATGTTCCTC

CTAATATCTACGCATTTCACCGCTACACTAGGAATTCCGCTTACCTCTCCTGCACTCCAGTCTGACAGTTTCAAAAGCAG

TCCCAGAGTTAAGCCCTGGGTTTTCACTTCTGACTTGCCATACCACCTACGCACCCTTTACACCCAGTAATTCCGGATAA

CGCTTGCCCCCTACGTATTACCGCGGCTGCTGGCACGTAGTTAGCCGGGGCTTCTTAGTCAGGTACCGTCATTTTCTTCC

CTGCTGATAGAGCTTTACATACCGAGATACTTCTTCACTCACGCGGCGTCGCTGCATCAGGGTTTCCCCCATTGTGCAAT

ATTCCCCACTGCTGCCTCCCGTAGGAGTTTGGGCCGTGTCTCAGTCCCAATGTGGCCGTTCACTCTCTCAAGCCGGCTAC

TGATCGTCGCCTTGGTAGGCCGTTACCCTGCCAACAAGCTAATCAGACGCGGGTCCATCCTGTACCACCGGAGTTTTTAC

CCCTGCACCATGCGGTGCTGTGGACTTATGCGGTATTAGCAGTCATTTCTAACTGTTATCCCCCTGTACAGGGCAGGTTA

C

>H5_27

GGAAGTTGAATTGACTGAGTGGCGGACGGGTGAGTAACGCGTGGGTAACCTGCCTTGTACTGGGGGACAACAGTTAGAAA

TGACTGCTAATACCGCATAAGCGCACAGTATCGCATGATACAGTGTGAAAAACTCCGGTGGTACAAGATGGACCCGCGTC

TGATTAGCTAGTTGGTAAGGTAACGGCTTACCAAGGCGACGATCAGTAGCCGACCTGAGAGGGTGACCGGCCACATTGGG

ACTGAGACACGGCCCAAACTCCTACGGGAGGCAGCAGTGGGGAATATTGCACAATGGGCGAAAGCCTGATGCAGCGACGC

CGCGTGAGTGAAGAAGTATTTCGGTATGTAAAGCTCTATCAGCAGGGAAGAAAATGACGGTACCTGACTAAGAAGCCCCG

GCTAACTACGTGCCAGCAGCCGCGGTAATACGTAGGGGGCAAGCGTTATCCGGATTTACTGGGTGTAAAGGGAGCGTAGA

CGGTAAAGCAAGTCTGAAGTGAAAGCCCGCGGCTCAACTGCGGGACTGCTTTGGAAACTGTTTAACTGGAGTGTCGGAGA

GGTAAGTGGAATTCCTAGTGTAGCGGTGAAATGCGTAGATATTAGGAGGAACACCAGTGGCGAAGGCGACTTACTGGACG

ATAACTGACGTTGAGGCTCGAAAGCGTGGGGAGCAAACAGGATTAGATACCCTGGTAGTCCACGCCGTAAACGATGAATA

CTAGGTGTTGGGGAGCAAAGCTCTTCGGTGCCGTCGCAAACGCAGTAAGTATTCCACCTGGGGAGTACGTTCGCAAGAAT

GAAACTCAAAGGAATTGACGGGGACCCGCACAAGCGGTGGAGCATGTGGTTTAATTCGAAGCAACGCGAAGAACCTTACC

AGGTCTTGACATCGATCCGACGGGGGAGTAACGTCCCCTTCCCTTCGGGGCGGAGAAGACAGGTGGTGCATGGTTGTCGT

CAGCTCGTGTCGTGAGATGTTGGGTTAAGTCCCGCAACGAGCGCAACCCTTATTCTAAGTAGCCAGCGGTTCGGCCGGGA

ACTCTTGGGAGACTGCCAGGGATAACCTGGAGGAAGGTGGGGATGACGTCAAATCATCATGCCCCTTATGATCTGGGCTA

CACACGTGCTACAATGGCGTAAACAAAGAGAAGCAAGACCGCGAGGTGGAGCAAATCTCAAAAATAACGTCTCAGTTCGG

ACTGCAGGCTGCAACTCGCCTGCACGAAGCTGGAATCGCTAGTAATCGCGAATCAGAATGTCGCGGTGAATACGTTCCCG

GGTCTTGTACACACCGCCCGTCACACCATGGGAGTCAGTAACGCCCGAAGTCAGTGACCCAACCGCAAGGAGGGAGCTGC

CGAAGGCGGGACCGATAACTGGGGTGAAGTCGTAACA

>H1_1

ACCGGCGCACGGGTGAGTAACGCGTATCCAACCTGCCCACCACTTGGGGATAACCTTGCGAAAGTAAGACTAATACCCAA

TGATATCTCTAGAAGACATCTGAAAGAGATTAAAGATTTATCGGTGATGGATGGGGATGCGTCTGATTAGCTTGTTGGCG

GGGTAACGGCCCACCAAGGCAACGATCAGTAGGGGTTCTGAGAGGAAGGTCCCCCACATTGGAACTGAGACACGGTCCAA

ACTCCTACGGGAGGCAGCAGTGAGGAATATTGGTCAATGGACGAGAGTCTGAACCAGCCAAGTAGCGTGCAGGAAGACGG

CCCTATGGGTTGTAAACTGCTTTTATAAGGGAATAAAGTGAGTCTCGTGAGACTTTTTGCATGTACCTTATGAATAAGGA

CCGGCTAATTCCGTGCCAGCAGCCGCGGTAATACGGAAGGTCCGGGCGTTATCCGGATTTATTGGGTTTAAAGGGAGCGT

AGGCCGGAGATTAAGCGTGTTGTGAAATGTAGATGCTCAACATCTGAACTGCAGCGCGAACTGGTTTCCTTGAGTACGCA

CAAAGTGGGCGGAATTCGTGGTGTAGCGGTGAAATGCTTAGATATCACGAAGAACTCCGATTGCGAAGGCAGCTCACTGG

AGCGCAACTGACGCTGAAGCTCGAAAGTGCGGGTATCGAACAGGATTAGATACCCTGGTAGTCCGCACGGTAAACGATGG

ATGCCCGCTGTTGGTCTGAATAGGTCAGCGGCCAAGCGAAAGCATTAAGCATCCCACCTGGGGAGTACGCCGGCAACGGT

GAAACTCAAAGGAATTGACGGGGGCCCGCACAAGCGGAGGAACATGTGGTTTAATTCGATGATACGCGAGGAACCTTACC

CGGGCTTGAATTGCAGAGGAAGGATTTGGAGACAATGACGCCCTTCGGGGCCTCTGTGAAGGTGCTGCATGGTTGTCGTC

AGCTCGTGCCGTGAGGTGTCGGCTTAAGTGCCATAACGAGCGCAACCCCTCTCCTTAGTTGCCATCAGGTTAAGCTGGGC

ACTCTGGGGACACTGCCACCGTAAGGTGTGAGGAAGGTGGGGATGACGTCAAATCAGCACGGCCCTTACGTCCGGGGCTA

CACACGTGTTACAATGGCAGGTACAGAGAGACGGTCCCTTGCAAAATGGATCAAATCCTTAAAGCCTGTCTCAGTTCGGA

CTGGGGTCTGCAACCCGACCCCACGAAGCTGGATTCGCTAGTAATCGCGCATCAGCCATGGCGCGGTGAATACGTTCCCG

GGCCTTGTACACACCGCCCGTCAAGCCATGAAAGCCGGGGGCGCCTAAAGTCCGTGACCGTAAGGAGCGGCC

>H5_37

CTGCTTTGATGAAGTTTTCGGATGGATTTAAAACAGCTTAGTGGCGGACGGGTGAGTAACGCGTGGGTAACCTGCCTCAC

ACTGGGGGATAACAGTTAGAAATAGCTGCTAATACCGCATAAGCGCACGGTTCCGCATGGAACAGTGTGAAAAACTCCGG

TGGTGTGAGATGGACCCGCGTCTGATTAGCCAGTTGGCGGGGTAACGGCCCACCAAAGCGACGATCAGTAGCCGGCCTGA

GAGGGTGAACGGCCACATTGGGACTGAGACACGGCCCAAACTCCTACGGGAGGCAGCAGTGGGGAATATTGCACAATGGG

GGAAACCCTGATGCAGCGACGCCGCGTGAGTGAAGAAGTATTTCGGTATGTAAAGCTCTATCAGCAGGGAAGAAAGTGAC

GGTACCTGAATAAGAAGCCCCGGCTAACTACGTGCCAGCAGCCGCGGTAATACGTAGGGGGCAAGCGTTATCCGGATTTA

CTGGGTGTAAAGGGAGCGTAGACGGCAAGGCAAGTCTGAAGTGAAAGCCCGGTGCTTAACGCCGGGACTGCTTTGGAAAC

TGTTTAGCTGGAGTGCCGGAGAGGTAAGCGGAATTCCTAGTGTAGCGGTGAAATGCGTAGATATTAGGAAGAACACCAGT

GGCGAAGGCGGCTTACTGGACGGTAACTGACGTTGAGGCTCGAAAGCGTGGGGAGCAAACAGGATTAGATACCCTGGTAG

TCCACGCCGTAAACGATGATTGCTAGGTGTAGGTGGGTATGGACCCATCGGTGCCGCAGCTAACGCAATAAGCAATCCAC

CTGGGGAGTACGTTCGCAAGAATGAAACTCAAAGGAATTGACGGGGACCCGCACAAGCGGTGGAGCATGTGGTTTAATTC

GAAGCAACGCGAAGAACCTTACCAGGTCTTGACATCCCGATGAAAAACCCGTAACGGGGTTCCCTCTTCGGAGCATCGGA

GACAGGTGGTGCATGGTTGTCGTCAGCTCGTGTCGTGAGATGTTGGGTTAAGTCCCGCAACGAGCGCAACCCTTATTCTT

AGTAGCCAGCAGGTAAGGCTGGGCACTCTAAGGAGACTGCCGGGGATAACCCGGAGGAAGGTGGGGATGACGTCAAATCA

TCATGCCCCTTATGATCTGGGCTACACACGTGCTACAATGGCGTAACAAAGGGAAGCGAGCCTGCGAGGGTGAGCAAATC

CCAAAAATAACGTCCCAGTTCGGACTGTAGTCTGCAACCCGACTACACGAAGCTGGAATCGCTAGTAATCGCGAATCAGA

ATGTCGCGGTGAATACGTTCCCGGGTCTTGTACACACCGCCCGTCACACCATGGGAGTCGGAAATGCCCGAAGTCTGTGA

CTCAACCGCAAGGAGAGAGCAGCCGAAGGCAGGTCTGATAACTGGGGTGAAGTCGT

>H3_4

GCAGTCGACGGAACTGTTTTGAAGATTTCTTCGGAATGAATTTGATTTAGTTTACTGGCGGACGGGTGAGTAACGCGTGA

GTAACCTGCCTTCAAGAGGGGGATAACATTCTGAAAAGGATGCTAATACCGCATGACATATCGGAACCACATGGTTTTGA

TATCAAAGATTTTATCGCTTGAAGATGGACTCGCGTCCGATTAGTTAGTTGGTGAGGTAACGGCTCACCAAGACCGCGAT

CGGTAGCCGGACTGAGAGGTTGAACGGCCACATTGGGACTGAGACACGGCCCAGACTCCTACGGGAGGCAGCAGTGGGGG

ATATTGCGCAATGGGGGCAACCCTGACGCAGCAACGCCGCGTGAAGGATGAAGGTTTTCGGATTGTAAACTTCTTTTATT

AAGGACGAAAAATGACGGTACTTAATGAATAAGCTCCGGCTAACTACGTGCCAGCAGCCGCGGTAATACGTAGGGAGCAA

GCGTTGTCCGGATTTACTGGGTGTAAAGGGTGCGTAGGCGGCTTTGCAAGTCAGATGTGAAATCTATGGGCTCAACCCAT

AAACTGCATTTGAAACTGTAGAGCTTGAGTGAAGTAGAGGCAGGCGGAATTCCCCGTGTAGCGGTGAAATGCGTAGAGAT

GGGGAGGAACACCAGTGGCGAAGGCGGCCTGCTGGGCTTTAACTGACGCTGAGGCACGAAAGCGTGGGTAGCAAACAGGA

TTAGATACCCTGGTAGTCCACGCTGTAAACGATGATTACTAGGTGTGGGGGGTCTGACCCCTTCCGTGCCGGAGTTAACA

CAATAAGTAATCCACCTGGGGAGTACGGCCGCAAGGTTGAAACTCAAAGGAATTGACGGGGGCCCGCACAAGCAGTGGAG

TATGTGGTTTAATTCGAAGCAACGCGAAGAACCTTACCAGGTCTTGACATCCAACTAACGAAGTAGAGATACATTAGGTG

CCCTTCGGGGAAAGTTGAGACAGGTGGTGCATGGTTGTCGTCAGCTCGTGTCGTGAGATGTTGGGTTAAGTCCCGCAACG

AGCGCAACCCTTGCTATTAGTTGCTACGCAAGAGCACTCTAATAGGACTGCCGTTGACAAAACGGAGGAAGGTGGGGACG

ACGTCAAATCATCATGCCCCTTATGACCTGGGCTACACACGTACTACAATGGATGTTAACAGAGGGAAGCAAGACAGCGA

TGTGGAGCAAACCCCTAAAAACATTCTCAGTTCAGATTGCAGGCTGCAACCCGCCTGCATGAAGATGGAATTGCTAGTAA

TCGCGGATCAGCATGCCGCGGTGAATACGTTCCCGGGCCTTGTACACACCGCCCGTCACACCATGGGAGCCGGTAATACC

CGAAGTCAGTAGTCCAACCTCGTGAGGACGCTGCCGAAGGTAGGATTGGCGACTGGGGTGAAGTCGTAACAAGGTAACCG

>H5_51

>H5_51

ACTGCGGCACTGAAGGGGTCAAACCTCCACACCTAGTAATCATCGTTTACGGTGTGGACTACCAGGGTATCTAATCCTGT

TTGCTACCCACACTTTCGAGCCTCAGCGTCAGTTGGTGCCCAGTAGGCCGCCTTCGCCACTGGTGTTCCTCCCGATATCT

ACGCATTCCACCGCTACACCGGGAATTCCGCCTACCTCTGCACTACTCAAGAAAAACAGTTTTGAAAGCAGTTTATGGGT

TGAGCCCATAGATTTCACTTCCGACTTGTCTTCCCGCCTGCGCTCCCTTTACACCCAGTAATTCCGGACAACGCTTGTGA

CCTACGTTTTACCGCGGCTGCTGGCACGTAGTTAGCCGTAACTTCCTTGTTGGGTACCGTCATTATCTTCCCCACAACAG

GAGTTTACAATCCGAAGACCTTCTTCCTCCACGCGGCGTCGCTGCATCAGGGTTTCCCCCATTGTGCAATATTCCCCACT

GCTGCCTCCCGTAGGAGTCTGGGCCGTGTCTCAGTCCCAATGTGGCCGTTCAACCTCTCAGTCCGGCTACCGATCGTCGC

CTTGGTGAGCCGTTACCTCACCAACTAGCTAATCGGACGCGAGGCCATCTCAAAGCGGAT

>H5_55

AGTCGACGGGGTGCTCATGACGGAGGATTCGTCCAATGGATTGAGTTACCTAGTGGCGGACGGGTGAGTAACGCGTGAGG

AACCTGCCTTGGAGAGGGGGATAACACTCCGAAAGGAGTGCTAATACCGCATGATGCAGTTGGGTCGCATGGCTCTGACT

GCCAAAGATTTATCGCTCTGAGATGGCCTCGCGTCTGATTAGCTAGTAGGCGGGGTAACGGCCCACCTAGGCGACGATCA

GTAGCCGGACTGAGAGGTTGACCGGCCACATTGGGACTGAGACACGGCCCAGACTCCTACGGGAGGCAGCAGTGGGGAAT

ATTGGGCAATGGGCGCAAGCCTGACCCAGCAACGCCGCGTGAAGGAAGAAGGCTTTCGGGTTGTAAACTTCTTTTGTCGG

GGACGAAACAAATGACGGTACCTGACGAATAAGCCACGGCTAACTACGTGCCAGCAGCCGCGGTAATACGTAGGTGGCAA

GCGTTATCCGGATTTACTGGGTGTAAAGGGCGTGTAGGCGGGATTGCAAGTCAGATGTGAAAACTGGGGGCTCAACCTCC

AGCCTGCATTTGAAACTGTAGTTCTTGAGTGCTGGAGAGGCAATCGGAATTCCGTGTGTAGCGGTGAAATGCGTAGATAT

ACGGAGGAACACCAGTGGCGAAGGCGGATTGCTGGACAGTAACTGACGCTGAGGCGCGAAAGCGTGGGGAGCAAACAGGA

TTAGATACCCTGGTAGTCCACGCCGTAAACGATGGATACTAGGTGTGGGGGGTCTGACCCCCTCCGTGCCGCAGTAACAC

AATAAGTATCCCACCTGGGGAGTACGATCGCAAGGTTGAAACTCAAAGGAATTGACGGGGGCCCGCACAAGCGGTGGAGT

ATGTGGTTTAATTCGAAGCAACGCGAAGAACCTTACCAGGGCTTGACATCCCACTAACGAAGCAGAGATGCGTTAGGTGC

CCTTCGGGGAAAGTGGAGACAGGTGGTGCATGGTTGTCGTCAGCTCGTGTCGTGAGATGTTGGGTTAAGTCCCGCAACGA

GCGCAACCCTTATTGTTAGTTGCTACGCAAGAGCACTCTAGCGAGACTGCCGTTGACAAAACGGAGGAAGGTGGGGACGA

CGTCAAATCATCATGCCCCTTATGTCCTGGGCCACACACGTACTACAATGGTGGTTAACAGAGGGAGGCAATACCGCGAG

GTGGAGCAAATCCCTAAAAGCCATCCCAGTTCGGATTGCAGGCTGAAACCCGCCTGTATGAAGTTGGAATCGCTAGTAAT

CGCGGATCAGCATGCCGCGGTGAATACGTTCCCGGGCCTTGTACACACCGCCCGTCACACCATGAGAGTCGGGAACACCC

GAAGTCCGTAGCCTAACCGCAAGGAGGGCGCGGCCGAAGGTGGGTTCGATAATTGGGGTGAAGTCGTAAC

>H5_58

AGTCGACGGACGAGGAGGAGCTTGCTTCTCCGAGTTAGTGGCGGACGGGTGAGTAACACGTGAGCAACCTACCCTTGAGA

GGGGGATAGCTTCTGGAAACGGATGGTAATACCCCATAACATATATTTTAGGCATCTAAGATATATCAAAGAAATTCGCT

CAAGGATGGGCTCGCGTCTGATTAGATAGTTGGTGAGGTAACGGCCCACCAAGTCGACGATCAGTAGCCGGACTGAGAGG

TTGAACGGCCACATTGGGACTGAGACACGGCCCAGACTCCTACGGGAGGCAGCAGTGGGGAATATTGCACAATGGGGGGA

ACCCTGATGCAGCGATGCCGCGTGGAGGAAGAAGGTTTTCGGATTGTAAACTCCTTTTAACAGGGACGATAATGACGGTA

CCTGAAGAAAAAGCTCCGGCTAACTACGTGCCAGCAGCCGCGGTAATACGTAGGGAGCGAGCGTTGTCCGGAATTACTGG

GTGTAAAGGGAGCGTAGGCGGGACGGTAAGTCAGGTGTGAAATATACGTGCTCAACATGTAGACTGCACTTGAAACTGCT

GTTCTTGAGTGAAGTAGAGGTAAGCGGAATTCCTAGTGTAGCGGTGAAATGCGTAGATATTAGGAGGAACATCGGTGGCG

AAGGCGGCTTACTGGGCTTTTACTGACGCTGAGGCTCGAAAGCGTGGGGAGCAAACAGGATTAGATACCCTGGTAGTCCA

CGCTGTAAACGATGATTACTAGGTGTGGGGGGACTGACCCCTTCCGTGCCGCAGTTAACACAATAAGTAATCCACCTGGG

GAGTACGACCGCAAGGTTGAAACTCAAAGGAATTGACGGGGGCCCGCACAAGCAGTGGAGTATGTGGTTTAATTCGAAGC

AACGCGAAGAACCTTACCAGGTCTTGACATCGTATGCATAGTCTAGAGATAGATGAAATCCCTTCGGGGACATATAGACA

GGTGGTGCATGGTTGTCGTCAGCTCGTGTCGTGAGATGTTGGGTTAAGTCCCGCAACGAGCGCAACCCTTACCTTTAGTT

GCTACGCAAGAGCACTCTAGAGGGACTGCCGTTGACAAAACGGAGGAAGGTGGGGATGACGTCAAATCATCATGCCCCTT

ATGACCTGGGCTACACACGTACTACAATGGCAATTAACAGAGGGAAGCAAAACAGCGATGTGGAGCAAATCCCGAAAAAT

TGTCCCAGTTCAGATTGCAGGCTGCAACTCGCCTGCATGAAGTCGGAATTGCTAGTAATCGCAGATCAGAATGCTGCGGT

GAATACGTTCCCGGGCCTTGTACACACCGCCCGTCACACCATGGGAGTCGGTAACACCCGAAGCCTGTAGTCTAACCTTA

TAGGAGGACGCAGTCGAAGGTGGGATTGATGACTGGGGTGAAGTCGT

>H5_66

GGATGAGGAAATGCTTCGGCATGGAGACATCCGATCTAGTGGCGGACGGGTGAGTAACGCGTGAGCAACCTGTCCTGCAC

AGGGGGATAACACTGAGAAATCAGTGCTAATACCGCATGAGACCACAGTATCACATGGTACAGGGGTCAAAGGAGAAATC

CGGTGCAGGGTGGGCTCGCGTCCCATTAGCTAGTTGGTAGGGTAAAGGCCTACCAAGGCGACGATGGGTAGCCGGACTGA

GAGGTTGGCCGGCCACACTGGGACTGAGACACGGCCCAGACTCCTACGGGAGGCAGCAGTGGGGAATATTGGGCAATGGG

CGAAAGCCTGACCCAGCAACGCCGCGTGAAGGAAGAAGGTCTTTGGATTGTAAACTTTTGTCCTATGGGAAGAAGGAAGT

GACGGTACCATGGGAGGAAGCCCCGGCTAACTACGTGCCAGCAGCCGCGGTAATACGTAGGGGGCGAGCGTTGTCCGGAA

TTACTGGGCGTAAAGGGCGCGCAGGCGGCCGATCAAGTTAGATGTGAAATACCCGGGCTTAACCTGGGAACTGCATTTAA

AACTGGTTGGCTAGGAGTGCAGGAGAGGGAAGCGGAATTCCTAGTGTAGCGGTGAAATGCGTAGATATTAGGAGGAACAC

CAGTGGCGAAGGCGGCTTTCTGGACTGTAACTGACGCTGAGGCGCGAAAGCGTGGGGAGCGAACAGGATTAGATACCCTG

GTAGTCCACGCTGTAAACGATGAATACTAGGTGTAGGGGGTATCGACCCCCCCTGTGCCGGAGCAAACGCAATAAGTATT

CCGCCTGGGGAGTACGGCCGCAAGGTTGAAACTCAAAGGAATTGACGGGGGCCCGCACAAGCAGCGGAGCATGTGGTTTA

ATTCGAAGCAACGCGAAGAACCTTACCAGGTCTTGACATCCCTCGAAGTGCATAGAGATATGTACGTCCTTCGGGACGAG

GAGACAGGTGGTGCATGGTTGTCGTCAGCTCGTGTCGTGAGATGTTGGGTTAAGTCCCGCAACGAGCGCAACCCCTACAG

TTAGTTACCAGCGGGTAAAGCCGGGGACTCTAACAGGACTGCCGTGGATAACACGGAGGAAGGTGGGGACGACGTCAAAT

CATCATGCTCCTTATGACCTGGGCTACACACGTGCTACAATGGCCGGTACAAAGAGAAGCGAGACCGTAAGGTGGAGCGG

ATCTCAAAAAACCGGTCCCAGTTCGGATTGTGGGCTGCAACCCGCCCACATGAAGTTGGAGTTGCTAGTAATCGCGAATC

AGCATGTCGCGGTGAATGCGTTCCCGGGCCTTGTACACACCGCCCGTCACACCATGGGAGTTGGGAGCGCCCGAAGTCGT

TGAGGTAACCCGCAAGGGAGCCAGGCGCCGAAGGTGAGACCGATAACTGGGGTGAAGTCGT

>H6_13

AGTCGAGCGAACCACTTCGGTGGTGAGCGGCGAACGGGTGAGTAACACGTAGGTGATCTGCCCATCAGACGGGGACAACG

ATTGGAAACGATCGCTAATACCGGATAGGACGAAAGTTTAAAGGTGCTTCTGGCACCGCTGATGGATGAGCCTGCGGCGC

ATTAGCTAGTTGGTAGGGTAAAGGCCTACCAAGGCGACGATGCGTAGCCGACCTGAGAGGGTGAACGGCCACACTGGGAC

TGAGACACGGCCCAGACTCCTACGGGAGGCAGCAGTAGGGAATCTTCGGCAATGGGCGAAAGCCTGACCGAGCAACGCCG

CGTGAATGATGAAGGCCTTCGGGTTGTAAAATTCTGTTATAAGGGAAGAACGACTTTAGTAGGAAATGGCTAGAGTGTGA

CGGTACCTTATGAGAAAGCCACGGCTAACTACGTGCCAGCAGCCGCGGTAATACGTAGGTGGCGAGCGTTATCCGGAATT

ATTGGGCGTAAAGAGCGCGCAGGTGGTTGATTAAGTCTGATGTGAAAGCCCACGGCTTAACCGTGGAGGGTCATTGGAAA

CTGGTCGACTTGAGTGCAGAAGAGGGAAGTGGAATTCCATGTGTAGCGGTGAAATGCGTAGAGATATGGAGGAACACCAG

TGGCGAAGGCGGCTTCCTGGTCTGTAACTGACACTGAGGCGCGAAAGCGTGGGGAGCAAACAGGATTAGATACCCTGGTA

GTCCACGCCGTAAACGATGAGTGCTAAGTGTTGGGGGTCGAACCTCAGTGCTGAAGTTAACGCATTAAGCACTCCGCCTG

GGGAGTACGGTCGCAAGACTGAAACTCAAAGGAATTGACGGGGACCCGCACAAGCGGTGGAGCATGTGGTTTAATTCGAA

GCAACGCGAAGAACCTTACCAGGTCTTGACATACCATTGACCGTTCTAGAGATAGGATTTTCCCTTCGGGGACAATGGAT

ACAGGTGGTGCATGGTTGTCGTCAGCTCGTGTCGTGAGATGTTGGGTTAAGTCCCGCAACGAGCGCAACCCCTGTCGTTA

GTTGCCAGCATTCAGTTGGGGACTCTAACGAGACTGCCAGTGACAAACTGGAGGAAGGTGGGGATGACGTCAAATCATCA

TGCCCCTTATGACCTGGGCTACACACGTGCTACAATGGTTGGTACAAAGAGAAGCGAAGCGGTGACGTGGAGCAAACCTC

ATAAAGCCAATCTCAGTTCGGATTGTAGGCTGCAACTCGCCTACATGAAGTTGGAATCGCTAGTAATCGCGAATCAGAAT

GTCGCGGTGAATACGTTCCCGGGTCTTGTACACACCGCCCGTCACACCACGAGAGTTTACAACACCCGAAGTCAGTGGCC

TAACCGCAAGGAGGGAGCTGCCTAAGGTGGGGTAGATGATTGGGGTGAAGTCGTAACAGGGTAACC

>H6_14

GAGTAATACATAAGTAACCTGGCATCTACAGGGGGATAACTGATGGAAACGTCAGCTAAGACCGCATAGGTGTAGAGATC

GCATGAACTCTATATGAAAAGTGCTACGGGACTGGTAGATGATGGACTTATGGCGCATTAGCTGGTTGGTAGGGTAACGG

CCTACCAAGGCGACGATGCGTAGCCGACCTGAGAGGGTGACCGGCCACACTGGGACTGAGACACGGCCCAGACTCCTACG

GGAGGCAGCAGTAGGGAATTTTCGGCAATGGGGGAAACCCTGACCGAGCAACGCCGCGTGAAGGAAGAAGTAATTCGTTA

TGTAAACTTCTGTCATAGAGGAAGAACGGTGGATATAGGGAATGATATCCAAGTGACGGTACTCTATAAGAAAGCCACGG

CTAACTACGTGCCAGCAGCCGCGGTAATACGTAGGTGGCGAGCGTTATCCGGAATTATTGGGCGTAAAGAGGGAGCAGGC

GGCACTAAGGGTCTGTGGTGAAAGATCGAAGCTTAACTTCGGTAAGCCATGGAAACCGTAGAGCTAGAGTGTGTGAGAGG

ATCGTGGAATTCCATGTGTAGCGGTGAAATGCGTAGATATATGGAGGAACACCAGTGGCGAAGGCGACGATCTGGCGCAT

AACTGACGCTCAGTCCCGAAAGCGTGGGGAGCAAATAGGATTAGATACCCTAGTAGTCCACGCCGTAAACGATGAGTACT

AAGTGTTGGGTGTCAAAGCTCAGTGCTGCAGTTAACGCAATAAGTACTCCGCCTGAGTAGTACGTTCGCAAGAATGAAAC

TCAAAGGAATTGACGGGGGCCCGCACAAGCGGTGGAGCATGTGGTTTAATTCGAAGCAACGCGAAGAACCTTACCAGGTC

TTGACATCGATCTAAAGGCTCCAGAGATGGAGAGATAGCTATAGAGAAGACAGGTGGTGCATGGTTGTCGTCAGCTCGTG

TCGTGAGATGTTGGGTTAAGTCCCGCAACGAGCGCAACCCCTGTTGCCAGTTGCCAGCATTAAGTTGGGGACTCTGGCGA

GACTGCCGGTGACAAGCCGGAGGAAGGCGGGGATGACGTCAAATCATCATGCCCCTTATGACCTGGGCTACACACGTGCT

ACAATGGACAGAGCAGAGGGAAGCGAAGCCGCGAGGTGGAGCGAAACCCATAAAACTGTTCTCAGTTCGGACTGCAGTCT

GCAACTCGACTGCACGAAGATGGAATCGCTAGTAATCGCGAATCAGCATGTCGCGGTGAATACGTTCTCGGGCCTTGTAC

ACACCGCCCGTCACACCATGAGAGTCGGTAACACCCGAAGCCGGTGGCCTAACCGCAAGGAAGGAGCTGTCTAAGGTGGG

ACTGATGATTGGGGTGAAGTCGT

>H6_15

CGGTTACCTTGTTACGACTTCACCCCAGTCATTGGTTTCACCTTCGACGGCCGCTTCCAAAAGGTTAGCTAACCGGCTTC

GGGCGCCCCCAACTCCCATGGTGTGACGGGCGGTGTGTACAAGACCCGGGAACGCATTCACCGCAGCATTCTGATCTGCG

ATTACTAGTAACTCCAGCTTCATGTAGGCGAGTTTCAGCCTACAATCCGAACTGAGAATGGCTTTAAGGGATTAGCTCCA

CCTCACGGTTTGGCAACCCTCTGTACCACCCATTGTAGCACGTGTGTAGCCCTAAGCATAAGGGGCATGATGATTTGACG

TCATCCCCACCTTCCTCCGAGTTATCCTCGGCAGTCCCTCTAGAGTGCCCAACTTAATGCTGGCAACTAAAGGCAAGGGT

TGCGCTCGTTGCGGGACTTAACCCAACATCTCACGACACGAGCTGACGACAACCATGCACCACCTGTCACCACTGTCCCC

GAAGGGAAATCTCCGATTAGGGAGAGGTCAATGGGATGTCAAGCTTAGGTAAGGTTCTTCGCGTTGCTTCGAATTAAACC

ACATGCTCCGCTACTTGTGCGGGTCCCCGTCAATTCCTTTGAGTTTCACTCTTGCGAGCGTACTTCCCAGGCGGAGTACT

TAATGCGTTAGCTGCGGCACCGAGGGGGGTAACCCCCGACACCTAGTACTCATCGTTTACGGCGTGGACTACCAGGGTAT

CTAATCCTGTTTGCTCCCCACGCTTTCGTGCCTCAGCGTCAGTTACAGTCCAGAGAGCCGCCTTCGCTACTGGTGTTCCT

CCTAATATCTACGCATTTCACCGCTACACTAGGAATTCCACTCTCCTCTCCTGCACTCAAGTCCTACAGTTCCAAAAGCT

TACTACGGTTGAGCCGTAGCCTTTCACTTCTGGCTTGAAAGACCGCCTACGCACCCTTTACGCCCAGTAATTCCGGATAA

CGCTAGCCCCCTACGTATTACCGCGGCTGCTGGCACGTAGTTAGCCGGGGCTTCCTCCTCAAGTACCGTCATTATCTTCC

TTGAGGACAGAGCTTTACGACCCGAAGGCCTTCATCGCTCACGCGGCGTTGCTGCATCAGGCTTTCGCCCATTGTGCAAT

ATTCCCCACTGCTGCCTCCCGTAGGAGTCTGGACCGTGTCTCAGTTCCAGTGTGGCCGATCACCCTCTCAGGTCGGCTAC

TGATCGTCGCCTTGGTAAGCCGTTACCTTACCAACTAGCTAATCAGACGCGGGTCCATCCTGTACCGCAAAAGCTTTGAT

ATTTTTATCATGCAATAAAAATATATCATCTCGTATTAGCATACCTTTCGGTATGTTATCCGTGTGTACAGGGCAGGTTA

CCCACGCGTTACTCACCCGTCCGCCGCTCTTCTCCGAAGAGAATCGCTCGACTTGCATGTGTTAGGCACGCCGCCAGCGT

TCATCCTGAGC

>H6_30

AGTCGAACGAAGCACTTATCTTTGATTCTTCGGATGAAGATGTTTGTGACTGAGTGGCGGACGGGTGAGTAACGCGTGGG

TAACCTGCCTCATACAGGGGGATAACAGTTAGAAATGACTGCTAATACCGCATAAGACCACGGAGCCGCATGGCTCAGTG

GGAAAAACTCCGGTGGTATGAGATGGACCCGCGTCTGATTAGGTAGTTGGTGGGGTAACGGCCTACCAAGCCAACGATCA

GTAGCCGACCTGAGAGGGTGACCGGCCACATTGGGACTGAGACACGGCCCAAACTCCTACGGGAGGCAGCAGTGGGGAAT

ATTGCACAATGGGGGAAACCCTGATGCAGCGACGCCGCGTGAGCGAAGAAGTATTTCGGTATGTAAAGCTCTATCAGCAG

GGAAGAAAATGACGGTACCTGACTAAGAAGCACCGGCTAAATACGTGCCAGCAGCCGCGGTAATACGTATGGTGCAAGCG

TTATCCGGATTTACTGGGTGTAAAGGGAGCGTAGACGGCTGTGTAAGTCTGAAGTGAAAGCCCGGGGCTCAACCCCGGGA

CTGCTTTGGAAACTATGCAGCTAGAGTGTCGGAGAGGTAAGTGGAATTCCCAGTGTAGCGGTGAAATGCGTAGATATTGG

GAGGAACACCAGTGGCGAAGGCGGCTTACTGGACGATGACTGACGTTGAGGCTCGAAAGCGTGGGGAGCAAACAGGATTA

GATACCCTGGTAGTCCACGCCGTAAACGATGACTACTAGGTGTCGGGGAGCAAAGCTCTTCGGTGCCGCAGCAAACGCAA

TAAGTAGTCCACCTGGGGAGTACGTTCGCAAGAATGAAACTCAAAGGAATTGACGGGGACCCGCACAAGCGGTGGAGCAT

GTGGTTTAATTCGAAGCAACGCGAAGAACCTTACCTGCTCTTGACATCCCGGTGACCGGCGTGTAATGACGCCTTTTCTT

CGGAACACCGGTGACAGGTGGTGCATGGTTGTCGTCAGCTCGTGTCGTGAGATGTTGGGTTAAGTCCCGCAACGAGCGCA

ACCCTTATCTTCAGTAGCCAGCATTTTGGATGGGCACTCTGGAGAGACTGCCAGGGATAACCTGGAGGAAGGTGGGGATG

ACGTCAAATCATCATGCCCCTTATGAGCAGGGCTACACACGTGCTACAATGGCGTAAACAAAGGGAAGCGAGCCTGCGAG

GGTAAGCAAATCTCAAAAATAACGTCTCAGTTCGGATTGTAGTCTGCAACTCGACTACATGAAGCTGGAATCGCTAGTAA

TCGCGAATCAGCATGTCGCGGTGAATACGTTCCCGGGTCTTGTACACACCGCCCGTCACACCATGGGAGTTGGTAACGCC

CGAAGTCAGTGACCCAACCGTAAGGAGGGAGCTGCCGAAGGTGGGACCGATAACTGGGGTGAAGTCGTAC

>H6_41

AGTCGAACGAGAGAAGAGAAGCTTGCTTTTCTGATCTAGTGGCGGACGGGTGAGTAACACGTGAGCAATCTGCCTTTCAG

AGGGGGATACCGATTGGAAACGATCGTTAATACCGCATAACATAATTGAACCGCATGATTTGATTATCAAAGATTTATCG

CTGAAAGATGAGCTCGCGTCTGATTAGCTAGTTGGTAAGGTAACGGCTTACCAAGGCGACGATCAGTAGCCGGACTGAGA

GGTTGATCGGCCACATTGGGACTGAGACACGGCCCAGACTCCTACGGGAGGCAGCAGTGGGGAATATTGCACAATGGAGG

AAACTCTGATGCAGCGATGCCGCGTGAGGGAAGAAGGTTTTAGGATTGTAAACCTCTGTCTTCAGGGACGAAAAAAAAGA

CGGTACCTGAGGAGGAAGCTCCGGCTAACTACGTGCCAGCAGCCGCGGTAATACGTAGGGAGCGAGCGTTGTCCGGAATT

ACTGGGTGTAAAGGGAGCGTAGGCGGGATCGCAAGTCAGATGTGAAAACTATGGGCTTAACCCATAAACTGCATTTGAAA

CTGTGGTTCTTGAGTGAAGTAGAGGTAAGCGGAATTCCTAGTGTAGCGGTGAAATGCGTAGATATTAGGAGGAACATCAG

TGGCGAAGGCGGCTTACTGGGCTTTAACTGACGCTGAGGCTCGAAAGCGTGGGGAGCAAACAGGATTAGATACCCTGGTA

GTCCACGCCGTAAACGATGATTACTAGGTGTGGGGGGACTGACCCCTTCCGTGCCGCAGCAAACGCAATAAGTAATCCAC

CTGGGGAGTACGACCGCAAGGTTGAAACTCAAAGGAATTGACGGGGGCCCGCACAAGCAGTGGAGTATGTGGATTAATTC

GAAGCAACGCGAAGAACCTTACCAGGTCTTGACATCGTATGCATAGCTCAGAGATGAGTGAAATCTCTTCGGAGACATAT

AGACAGGTGGTGCATGGTTGTCGTCAGCTCGTGTCGTGAGATGTTGGGTTAAGTCCCGCAACGAGCGCAACCCTTACTGT

TAGTTGCTACGCAAGAGCACTCTAGCAGGACTGCCGTTGACAAAACGGAGGAAGGTGGGGATGACGTCAAATCATCATGC

CCCTTATGACCTGGGCCTCACACGTACTACAATGGCTGTCAACAGAGGGATGCAAAGCCGCGAGGTGGAGCGAACCCCTA

AAAGCAGTCTTAGTTCGGATTGTAGGCTGCAACCCGCCTACATGAAGTCGGAATTGCTAGTAATCGCAGATCAGCATGCT

GCGGTGAATACGTTCCCGGGCCTTGTACACACCGCCCGTCACGCCATGGGAGTCGGTAACACCCGAAGCCTGTAGTCTAA

CCGCAAGGAGGACGCAGTCGAAGGTGGGATTGATGACTGGGGTGAAGTCGTAACAAGGTAACCG

>H1_10

TACATAGGTAACCTGCGCCTGTGCGGGGGATAACAGGAGGAAACTCCTGCTAATACCGCATAGCCATGAGCACCGCATGG

AGCTCATGCCAAATATCCTTTATGGGATAGCGCAGGGATGGACCTATGGCGCATTAGCTTGTTGGCGGGGCAACGGCCCA

CCAAGGCAACGATGCGTAGCCGGCCTGAGAGGGCGGACGGCCACATTGCGACTGAGACACGGCCCAGACTCCTACGGGAG

GCAGCAGTACGGAATTTTCGGCAATGGGGGAAACCCTGATCGAGCAACGCCGCGTGAGCGAAGAAGGCCTTCGGGTCGTA

AAGCTCTGTTGTAAAGGAAGAACGTCGCATGCAGGAAATGGTGTGCGAGTGACGGTACTTTACCAGAAAGCCACGGCTAA

CTACGTGCCAGCAGCCGCGGTAATACGTAGGTGGCGAGCGTTATCCGGAATCATTGGGCGTAAAGAGGGAGCAGGCGGCC

GCAAGGGTCTGTGGTGAAAGACCGAAGCTAAACTTCGGTGAGCCATGGAAACCGGGCGGCTAGAGTGCGGAAGAGGATCG

TGGAATTCCATGTGTAGCGGTGAAATGCGTAGATATATGGAGGAACACCAGTGGCGAAGGCGACGGTCTGGGCCGCAACT

GACGCTCATTCCCGAAAGCGTGGGGAGCAAATAGGATTAGATACCCTAGTAGTCCACGCCGTAAACGATGGTCACTAAGT

GTCGGGGGTCAAACCCCCGGT

>H3_3

AGTCGACGAAGCTTTGAGGAGCTTGCTTTTTAAGCTTAGTGGCGGACGGGTGAGTAACGCGTGAGCAACCTGCCTCTCAG

AGGGGAATAACGTTTTGAAAAGAACGCTAATACCGCATAACATATCGGAACCGCATGATTCTGATATCAAAGGAGCAATC

CGCTGAGAGATGGGCTCGCGTCCGATTAGTTAGTTGGTGAGGTAACGGCTCACCAAGACTACGATCGGTAGCCGGACTGA

GAGGTTGATCGGCCACATTGGGACTGAGACACGGCCCAGACTCCTACGGGAGGCAGCAGTGGGGGATATTGCGCAATGGG

GGAAACCCTGACGCAGCAACGCCGCGTGAAGGAAGAAGGTCTTCGGATTGTAAACTTCTTTTGTCAGGGACGAAGAAAGT

GACGGTACCTGACGAATAAGCTCCGGCTAACTACGTGCCAGCAGCCGCGGTAATACGTAGGGAGCGAGCGTTGTCCGGAT

TTACTGGGTGTAAAGGGTGCGTAGGCGGCCGAGCAAGTCAGTTGTGAAAACTATGGGCTTAACCCATAACGTGCAATTGA

AACTGTCCGGCTTGAGTGAAGTAGAGGTAGGCGGAATTCCCGGTGTAGCGGTGAAATGCGTAGAGATCGGGAGGAACACC

AGTGGCGAAGGCGGCCTACTGGGCTTTAACTGACGCTGAGGCACGAAAGCATGGGTAGCAAACAGGATTAGATACCCTGG

TAGTCCATGCCGTAAACGATGATTACTAGGTGTGGGGGGACTGACCCCTTCCGTGCCGCAGTTAACACAATAAGTAATCC

ACCTGGGGAGTACGGCCGCAAGGTTGAAACTCAAAGGAATTGACGGGGGCCCGCACAAGCAGTGGAGTATGTGGTTTAAT

TCGAAGCAACGCGAAGAACCTTACCAGGTCTTGACATCCTGAGAATCCTTAAGAGATTAGGGAGTGCCTTCGGGAACTCA

GAGACAGGTGGTGCATGGTTGTCGTCAGCTCGTGTCGTGAGATGTTGGGTTAAGTCCCGCAACGAGCGCAACCCTTGCTA

TTAGTTGCTACGCAAGAGCACTCTAATAGGACTGCCGTTGACAAAACGGAGGAAGGTGGGGACGACGTCAAATCATCATG

CCCCTTATGACCTGGGCTACACACGTACTACAATGGCCATTAACAGAGGGAAGCAAAACCGCGAGGCAGAGCAAACCCCT

AAAAATGGTCCCAGTTCGGATTGTAGGCTGCAACCCGCCTACATGAAGTTGGAATTGCTAGTAATCGCGGATCAGCATGC

CGCGGTGAATACGTTCCCGGGCCTTGTACACACCGCCCGTCACACCATGGGAGCCGGTAATACCCGAAGTCAGTAGTCTA

ACAGCAATGAGGACGCTGCCGAAGGTAGGATTGGCGACTGGGGTGAAGTCGTAACAAGGTAACCG

>H5_11

GCTTGCTTCCAAGAGACTTAGTGGCGAACGGGTGAGTAACACGTAGGTAACCTGCCCATGTGCCCGGGATAACTGCTGGA

AACGGTAGCTAAAACCGGATAGGTATGAGGGAGGCATCTTCCTCATATTAAAGCACCTTCGGGTGTGAACATGGATGGAC

CTGCGGCGCATTAGCTGGTTGGTGAGGTAACGGCCCACCAAGGCGATGATGCGTAGCCGACCTGAGAGGGTGAACGGCCA

CATTGGGACTGAGACACGGCCCAAACTCCTACGGGAGGCAGCAGTAGGGAATTTTCGTCAATGGGGGGAACCCTGAACGA

GCAATGCCGCGTGTGTAAAGAAGGTCTTCGGATCGTAAAGCACTGTTGTAAGTGAAGAACGCCACATAGAGGAAATGCTA

TGTGGGTGACGGTAGCTTACCAGAAAGCCACGGCTAACTACGTGCCAGCAGCCGCGGTAATACGTAGGTGGCAAGCGTTA

TCCGGAATCATTGGGCGTAAAGGGTGCGTAGGTGGCACGATAAGTCTGAAGTAAAAGGCAACAGCTCAACTGTTGTATGC

TTTGGAAACTGTCGAGCTAGAGTGCAGAAGAGGGCGATGGAATTCCATGTGTAGCGGTAAAATGCGTAGATATATGGAGG

AACACCAGTGGCGAAGGCGGTCGCCTGGTCTGTAACTGACACTGATGCACGAAAGCGTGGGGAGCAAATAGGATTAGATA

CCCTAGTAGTCCACGCCGTAAACGATGAGAACTAAGTGTTGGAGAGATTCAGTGCTGCAGTTAACGCAATAAGTTCTCCG

CCTGGGGAGTATGCACGCAAGTGTGAAACTCAAAGGAATTGACGGGGGCCCGCACAAGCGGTGGAGTATGTGGTTTAATT

CGAAGCAACGCGAAGAACCTTACCAGGCCTTGACATGGATATAAATGTTCTAGAGATAGAAAGATAGCTATATATCACAC

AGGTGGTGCATGGTTGTCGTCAGCTCGTGTCGTGAGATGTTGGGTTAAGTCCCGCAACGAGCGCAACCCTTGTCTTCTGT

TACCAGCATTAAGTTGGGGACTCAGGAGAGACTGCCGGTGACAAACCGGAGGAAGGTGGGGATGACGTCAAATCATCATG

CCCCTTATGGCCTGGGCTACACACGTACTACAATGGCGCCTACAAAGAGCAGCGACACCGCGAGGTGGAGCGAATCTCAT

AAAGGGCGTCTCAGTTCGGATTGAAGTCTGCAACTCGACTTCATGAAGTCGGAATCGCTAGTAATCGCAGATCAGCATGC

TGCGGTGAATACGTTCTCGGGCCTTGTACACACCGCCCGTCAAACCATGGGAGTTGGTAATACCCGAAGCCGGTGGCATA

ACCGCAAGGAGTGAGC

>H5_45

TTCTCGACTTAGTGGCGGACGGGTGAGTAACGCGTGAGCAATCTGCCTTTAAGAGGGGGATAACAGTCGGAAACGGCTGC

TAATACCGCATAAAGCATTAAATTCGCATGTTTTTGATGCCAAAGGAGCAATCCGCTTTTAGATGAGCTCGCGTCTGATT

AGCTAGTTGGCGGGGTAACGGCCCACCAAGGCGACGATCAGTAGCCGGACTGAGAGGTTGAACGGCCACATTGGGACTGA

GACACGGCCCAGACTCCTACGGGAGGCAGCAGTGGGGAATATTGCGCAATGGGGGAAACCCTGACGCAGCAACGCCGCGT

GATTGAAGAAGGCCTTCGGGTTGTAAAGATCTTTAATCAGGGACGAAATAAATGACGGTACCTGAAGAATAAGCTCCGGC

TAACTACGTGCCAGCAGCCGCGGTAATACGTAGGGAGCAAGCGTTATCCGGATTTACTGGGTGTAAAGGGCGCGCAGGCG

GGCCGGTAAGTTGGAAGTGAAATCTATGGGCTTAACCCATAAACTGCTTTCAAAACTGCTGGTCTTGAGTGATGGAGAGG

CAGGCGGAATTCCGTGTGTAGCGGTGAAATGCGTAGATATACGGAGGAACACCAGTGGCGAAGGCGGCCTGCTGGACATT

AACTGACGCTGAGGCGCGAAAGCGTGGGGAGCAAACAGGATTAGATACCCTGGTAGTCCACGCCGTAAACGATGGATACT

AGGTGTGGGAGGTATTGACCCCTTCCGTGCCGCAGTTAACACAATAAGTATCCCACCTGGGGAGTACGGCCGCAAGGTTG

AAACTCAAAGGAATTGACGGGGGCCCGCACAAGCAGTGGAGTATGTGGTTTAATTCGAAGCAACGCGAAGAACCTTACCA

GGTCTTGACATCCCGATGACCGTCCTAGAGATAGGGCTTTTCTTCGGAACATCGGTGACAGGTGGTGCATGGTTGTCGTC

AGCTCGTGTCGTGAGATGTTGGGTTAAGTCCCGCAACGAGCGCAACCCTTACGGTTAGTTGATACGCAAGATCACTCTAG

CCGGACTGCCGTTGACAAAACGGAGGAAGGTGGGGACGACGTCAAATCATCATGCCCCTTATGACCTGGGCTACACACGT

ACTACAATGGCAGTCATACAGAGGGAAGCAAAACCGCGAGGTGGAGCAAATCCCTAAAAGCTGTCCCAGTTCAGATTGCA

GGCTGCAACCCGCCTGCATGAAGTCGGAATTGCTAGTAATCGCGGATCAGCATGCCGCGGTGAATACGTTCCCGGGCCTT

GTACACACCGCCCGTCACACCATGAGAGCCGTCAATACCCGAAGTCCGTAGCCTAACCGTAAGGAAGGCGCGGCCGAAGG

TAGGGGTGGTAATTAAGGTGAAGT
